# Supplementary material for: TwinCons: Conservation score for uncovering deep sequence similarity and divergence
Source: PLoS Comput Biol. 2021 Oct 29;17(10):e1009541. doi: 10.1371/journal.pcbi.1009541 (PMC8580257; doi:10.1371/journal.pcbi.1009541)
Supplement: S1 Appendix — Fig A. ROC curves for classifiers with different parameters built from the BaliBASE dataset, tested against the rProt dataset. Parameters shown here are segment boundaries (length threshold), TWC intensity for detection of positive positions (intensity threshold), and what percentage gaps should be used for removal of alignment columns (gap threshold). Each subplot represents different combination of the intensity and length thresholds. Colored lines within subplots represent different gap thresholds. Cutting only alignment positions with more than 80–90% gaps produce better distinction between true positive and true negatives. Complete data including all tested parameters and datasets is available in S2 Dataset. Fig B. ROC curves of training classifiers with different penalties and gamma parameters. The first four subplots (A-D) test different penalties and the last four test different gamma values (E-H). Each subplot represents a different dataset that was used for training and testing. (A) and (E) are PROSITE, (B) and (F) are BaliBASE, (C) and (G) are INDELible, (D) and (H) are rProtein dataset. For testing each dataset was split in 3 folds. Each fold produces an ROC curve, we plot the mean of the three results as single curve and plot the standard deviation of the true positive rate as a shaded region around it. Complete data is available in S3 Dataset. Fig C. ROC curves generated from HHalign alignments from the four datasets: BaliBASE, rProtein, INDELible, and PROSITE. Colored lines within subplots represent different gap thresholds used for column exclusion. Fig D. Comparison of structural mapping between Zebra2 and TwinCons. A) Zebra2 results and B) TwinCons results from sequence alignment for uL2 between archaeal and bacterial sequences mapped on the E. coli uL2 structure from PDB 4V9D [22]. C) Zebra2 results and D) TwinCons results from the same sequence alignment mapped on the P. furiosus uL2 structure from PDB 4V6U [36]. In panels A) and C) red indicates [file pcbi.1009541.s001.pdf]

Appendix for the article:

# TwinCons: conservation score for uncovering deep sequence similarity and divergence

Petar I. Penev[1,2], Claudia Alvarez-Carreño[1,3], Eric Smith[1,4,5,6,7], Anton S. Petrov[1,3]*, and Loren Dean Williams[1,2,3]*

[1]NASA Center for the Origin of Life, Georgia Institute of Technology, Atlanta, Georgia, USA
[2]School of Biological Sciences, Georgia Institute of Technology, Atlanta, Georgia, USA
[3]School of Chemistry and Biochemistry, Georgia Institute of Technology, Atlanta, Georgia, USA
[4]Earth-Life Science Institute, Tokyo Institute of Technology, Meguro-ku, Tokyo, Japan
[5]Santa Fe Institute, Santa Fe, New Mexico, USA
[6]Department of Physics, The University of Wisconsin-Madison, Madison, Wisconsin, USA
[7]Ronin Institute, Montclair, New Jersey, USA

* loren.williams@chemistry.gatech.edu (LDW); * anton.petrov@biology.gatech.edu (ASP)

**This PDF file includes:**

Supplementary text
Figures A to L
Tables A to E
Legends for Datasets S1 to S10
Supplementary Information References

**Other supplementary materials for this manuscript include the following:**

Datasets S1 to S10

## The likelihood model underlying TwinCons scoring

The TwinCons score is a log-likelihood for paired sets of aligned residues, sampled from the leaves of a branching process generated by the chosen substitution matrix.

Denote by $N$and $N'$the number of sequences in the first and second groups; these are then the numbers of residues in the two groups at each position.

Let $i$ or $j$ label the possible residues (amino acids or nucleotides) and denote the counts of residues in the two groups by vectors $n \equiv [n_i]$ and $n' \equiv [n'_j]$, respectively. Then $\sum_i n_i = N$, $\sum_j n'_j = N'$.

## Extending pairwise scoring to pairs of sets

A pairwise alignment score may be assigned as a log-likelihood to observe any pair of residues $(i, j)$at a single position. The standard measure of significance assigned to pairs under a hypothesis about their separation from a common ancestor is the logarithm of the pair frequency sampled from the leaves of a branching process of the hypothesized depth, with empirically calibrated transition frequencies. The log-likelihood for residues at a single position is measured not in absolute terms, but relative to the log of the product of marginal probabilities representing the background frequencies under the same generating process at uncorrelated residues. These differences of log-likelihood measures are given in standard form as substitution matrices [1, 2].

We wish to extend this score to pairs of sets with no other assumptions of structure within the sets. Because aligned residues both within each group and between groups are considered conditionally independent given the branching process, they are treated as independent samples from the leaves of the process. The joint likelihoods for sets of samples are therefore products. The log-likelihoods that naturally extend the single-pair score to sets of pairs, with the only new conditions being the way the user has grouped the data, are naturally bilinear functions of the frequencies of residues in the two groups. Whereas, for amino acid substitution in proteins, a standard form exists for PAM and BLOSUM matrices as log-likelihood ratios with known branching processes, for nucleotides the time depth, overall magnitude of the scoring matrix, and baseline substitution will need to be standardized if these measures are to be compared across datasets, or between nucleic acids and proteins.

We construct a group-level joint probability as a uniformly weighted probability over all pairs of the residue from one aligned sequence in one group and the residue from one aligned sequence in the other. For a pair of residue types $(i, j)$in the first and second groups respectively, the number of pairs with those residues becomes $n_i n_j$. Thus, we wish to assign a probability to the two aligned groups that is proportional to the probability to independently sample $NN'$random pairs of residues, obtaining for each pair of residue types $(i, j)$a fraction $\frac{n_i n_j}{NN'}$. The notion of independence and uniform weighting is thus defined in terms of a product measure on pairs.

## Substitution matrix, consistent distribution, and log-likelihood

Let $s \equiv [s_{ij}]$ denote the substitution matrix for the branching process. It is defined as in

[1] from the log-likelihood of pair probabilities $q \equiv [q_{ij}]$ by

$$\lambda s_{ij} \quad = \log\left(\frac{q_{ij}}{p_i p'_j}\right), \tag{1}$$

where the self-consistent marginals $p$and $p'$, known as *target frequencies*, are computed from $q$ as

$$p_i \quad = \sum_j q_{ij} \qquad p'_j \quad = \sum_i q_{ij}\,. \tag{2}$$

$\lambda$ is a scale factor relating a convenient base for information units to the log-likelihood ratio. The likelihood to independently sample a collection $\{n_i n_j\}_{(i,j)}$of pairs summing to $NN'$from a branching process in which the pair probabilities on leaves are given by $q_{ij}$is the multinomial distribution

$$l(n,n') \quad = \binom{NN'}{nn'} \prod_{i,j} q_{ij}^{n_i n'_j}\,. \tag{3}$$

The multinomial coefficient in Eq. (3), treating all indices $(i,j)$as independent, is given by

$$\binom{NN'}{nn'} \quad \equiv \frac{(NN')!}{\prod_{i,j}(n_i n'_j)!}\,. \tag{4}$$

In Stirling's approximation, the log-likelihood per sample is given by

$$\begin{aligned} \frac{\log l(n,n')}{NN'} &= \sum_{i,j} \frac{n_i}{N}\frac{n'_j}{N'} \log q_{ij} \\ &- \sum_i \frac{n_i}{N} \log\left(\frac{n_i}{N}\right) - \sum_j \frac{n'_j}{N'} \log\left(\frac{n'_j}{N'}\right) \\ &= \sum_{i,j} \frac{n_i}{N}\frac{n'_j}{N'} \log\left(\frac{q_{ij}}{p_i p'_j}\right) \\ &- \sum_i \frac{n_i}{N} \log\left(\frac{n_i}{p_i N}\right) - \sum_j \frac{n'_j}{N'} \log\left(\frac{n'_j}{p'_j N'}\right) \end{aligned} \tag{5}$$

We recognize the Kullback-Leibler divergence,

$$D(\frac{\mathrm{n}}{N}\,||p) \quad = \sum_i \frac{n_i}{N} \log\left(\frac{n_i}{p_i N}\right) \tag{6}$$

as a negative log-likelihood to draw the sample $\{n_i\}_i$ from the marginals of a branching process

for which the pair distribution is $q_{ij}$ (and likewise for $n_j$and $p'_j$). It measures the excess information within groups about the mismatch to the branching process used as a null model. The within-group sample probability can be maximized (Kullback-Leibler divergence forced to zero) by adjusting target probabilities $p$and $p'$to probabilities $P$and $P'$that match the residue frequencies in the aligned samples, as in [3], while maximally retaining the information in pair correlations. Alternatively, these marginal divergences may be used as-is to quantify non-representativeness within groups of the null model.

Combining the definition (1) with the notation (6), we may write the TwinCons score in terms of these log-likelihood factors as

$$\lambda \sum_{i,j} \frac{n_i}{N} \frac{n'_j}{N'} \log s_{ij} = \frac{\log l(n,n')}{NN'} + D(\tfrac{n}{N}||p) \ + D(\tfrac{n}{N}||p') \tag{7}$$

## Other sampling interpretations with the same log-likelihood value

The likelihood (3), which treats each pair-label $(i,j)$as an independent set of samples, does not explicitly account for the property that the sample numbers $\{n_i n_j\}_{(i,j)}$are *constructed* to have a product form, and are thus not all independent. The variables that are independent are only the two sets $\{n_i\}_i$, $\{n_j\}_j$, summing to $N + N'$.

The uniformly-weighted group-level probability on only the independent sample values, which corresponds to TwinCons, assigns to each aligned sequence in the first group with residue $i$a probability $\left(\prod_j q_{ij}\right)^{\frac{1}{N'}}$ – the geometric mean of pair probabilities for all pairs it may form in the second group – and to each aligned sequence in the second group with residue $j$a probability $\left(\prod_i q_{ij}\right)^{\frac{1}{N}}$.

The resulting likelihood conditioned on groupings may be written

$$\begin{aligned} l(n,n' \mid \text{grps}) &= \binom{N}{n}\binom{N'}{n'} \prod_{i,j} q_{ij}^{\,n_i\left(\frac{n'_j}{N'}\right)+\left(\frac{n_i}{N}\right)n'_j} \\ &= \binom{N}{n}\binom{N'}{n'} \prod_{i,j} q_{ij}^{\frac{(N+N')n_i n'_j}{NN'}} . \end{aligned} \tag{8}$$

The multinomial distributions in Eq. (8) now count only the partition of independent samples of aligned residues within each group:

$$\binom{N}{n} \equiv \frac{N!}{\prod_i n_i!} \quad \binom{N'}{n'} \equiv \frac{N'!}{\prod_j n_i'!}. \qquad (9)$$

The likelihood (8) sums to unity over all independent partitions $n$of $N$and $n'$of $N'$.

Stirling's approximation then gives that

$$\frac{\log l\big(n, n'|\text{grps}\big)}{N+N'} = \frac{\log l(n,n')}{NN'} \qquad (10)$$

confirming equivalence of the models Eq. 3 and Eq. 8.

## TwinCons offset from the mean of its expected distribution

The log of the sample likelihood from the leaves of the branching process is a random variable. Under independent pair samples from the branching process generated by $s$, the expectation of $\lambda s$ is the relative entropy

$$\lambda \sum_{i,j} q_{ij} s_{ij} = \sum_{i,j} q_{ij} \log\left(\frac{q_{ij}}{p_i p_j'}\right) = D(q||pp'), \qquad (11)$$

the Kullback-Leibler divergence of $q$from the product of its marginals, or the mutual information in one marginal about the other.

The pair frequencies produced from two groups of aligned residues are, by construction, the products of their marginals. Therefore, the closest approximation they can give to the mean under the full branching process is the dual Kullback-Leibler divergence

$$D(pp'||q) = \sum_{i,j} p_i p_j' \log\left(\frac{p_i p_j'}{q_{ij}}\right). \qquad (12)$$

If $q$is not too far from the product of its marginals, then the two divergences (11) and (12) will be similar in magnitude.

We display TwinCons scores offset from the divergence (12) for consistency with the constraints on the input data. If the user chooses to adjust $p$and $p'$to distributions $P$and $P'$matching the sample frequencies, the offset is exactly a subtraction of the sample frequency at each position from its expectation in the data.

## *Generating alignments from random sequences*

We used INDELible [4] to generate a set of alignments with related (true positive, TP) and unrelated (true negative, TN) groups. First, we generated 22 alignments from randomized sequence seeds of length 250 following the LG substitution model. Each alignment followed a random tree with 120 leaves. The control file with parameters for INDELible, used for tree and sequence generation is available in the Supplementary Material (Supp. Dataset 8). Every sequence within an alignment originates from the same initial random sequence, so these alignments were used as a TP set by splitting the 120 sequences in two groups of 60. From the original 22 alignments 20 were selected and iteratively combined with mafft-profile [5, 6], resulting in 190 TN alignments where the two groups are not related since they originate from different random sequences. The random sequence generation algorithm did not produce a satisfyingly different initial random sequences for 11 combinations of TN alignments, these alignments were removed from further analysis.

## Generating alignments from biological sequences

*Ribosomal protein dataset*

We used highly curated alignments of rProteins to generate an additional set of TP and TN composite alignments. Sequences for these alignments were gathered for 67 bacterial and 55 archaeal species from the Sparse and Efficient Representation of the Extant Biology database (SEREB)[7]. TP composite alignments were created using the group separation between archaeal and bacterial sequences within a single rProtein alignment. TN composite alignments were generated from small subunit rProteins combined with the program mafft-profile [5, 6]. We used small subunit rProteins since they generally lack extended unstructured regions and do not share structural folds.

*BaliBASE dataset*

We also generated a set of TP and TN alignments from the BAliBASE multiple alignment suite [8]. The BaliBASE database of alignments has different structure-based reference alignments used for alignment algorithm evaluation. We used alignments from reference 3 where sequences are part of groups with less 20% identities between any two sequences across groups. Each one of the 30 alignments in this reference set can have more than two groups. We manually truncated the alignments to contain only two groups, this generated our TP set. To ensure gaps between TN composite alignments are preserved we excluded alignments with less than 20 sequences in a group. We iteratively combined the alignment groups that contain more than 20 sequences, ensuring no groups with similar structural folds or functions are in one TN composite alignment. Combinations of alignments that were excluded are available in Supp. Dataset 1. This produced 141 TN composite alignments and 38 TP composite alignments.

*PROSITE dataset*

Additionally, we used the PROSITE database of protein patterns and profiles [9, 10] to

generate a set of TP and TN composite alignments. The PROSITE database has 1826 documentation entries in the 2019 February release. Each documentation entry describes single or multiple motifs that have an alignment associated with it. We generated TP composite alignments by pairwise combination of motif alignments from the same documentation entry. TN composite alignments were generated by combining motif alignments from different documentation entries. To ensure that groups within our merged alignments where of comparable size, we removed PROSITE motif alignments with less than 20 sequences and more than 100 sequences. Furthermore, we filtered out PROSITE motif alignments with length less than 50 residues and more than 500 residues. This generated 120 TP composite alignments and 36,856 TN composite alignments.

## SVM classifier formula and features

The optimization formula for the SVM classifier with a 'rbf' kernel, used throughout the manuscript is:

$$\exp\left(-\gamma\|x - x'\|^2\right) \quad (13),$$

where $\gamma$ is the influence of each training example, our parametrization sets gamma to 0.5.

The minimum and maximum values used to normalize each testing segment are:

- Minimum weight: 5.425
- Maximum weight: 52.092
- Minimum length coverage: 0.005
- Maximum length coverage: 0.338

Weight is the sum of TwinCons scores for a given segment, length coverage is the percentage of the alignment positions a given segment covers.

**Signature and conservation thresholds.**

To determine a threshold for signature positions in TwinCons results from composite alignments we used a k-means clustering algorithm. By separating all TwinCons scores from a given alignment in k number of groups, we determine the thresholds that separate these groups. These thresholds can be used to determine highly conserved or signature scores for the given alignment. TwinCons scores depend on the matrix being used. Therefore, protein and rRNA scores will differ. For that reason, signature thresholds are determined for a particular alignment or groups of alignments, using the same matrix. We applied the same algorithm to rRNA and protein results, using 5 k-clusters, ensuring that even complex TwinCons score distributions have sufficient peak distinction. S7 Dataset holds TwinCons results from protein and RNA alignments with their conservation and signature thresholds.

**rRNA signature and conservation thresholds**

Distribution of TwinCons scores from different rRNA parts of the ribosome were inspected to check whether limiting input data would affect scores. The distributions produced

from composite alignments of the entire rRNA (23S+16S+5S) show three peaks. A small peak around the minimal possible value (-2.25), another peak around TwinCons score of zero, and third peak around the maximal possible value (6.75) (Fig I). Distributions from subsets of 23S or 16S show the same peaks, with lower intensity. To determine the boundary for signature and conserved nucleotides we used increasing number of k-clusters (starting from 3) with the scipy python library [11] until each distribution peak was placed in separate group (Fig I). The lowest number of k-clusters that produced clear distinction was 5. Different subsets of rRNA produced different thresholds (Table B). The subset including only 16S rRNA produced the most conservative threshold of -0.75 and the lowest standard deviation from 100 runs. Therefore, we selected -0.75 as threshold for signatures and 5.55 as threshold for conserved nucleotides.

**Protein signature and conservation thresholds**

Distribution of TwinCons score from protein alignments generally take the Gaussian form. Therefore, using 3 k-clusters would be sufficient to identify thresholds for signature and conserved amino acids. We elected to use 5 k-clusters to be consistent with the rRNA methodology.

**Caspase and metacaspase analysis.**

To study differences between caspases and metacaspases, we selected a subset of 14 metazoan Caspase-9 and 11 non-metazoan eukaryotic metacaspase sequences. We generated a structurally guided MSA for this subset of sequences. Both TwinCons and Zebra2 was computed for the same MSA and mapped on available 3D structures of caspases (PDB ID: 1JXQ [12]) and metacaspases (PDB ID: 4F6O [13]). TwinCons was calculated using structure inferred substitution matrices [14] and the blosum62 matrix (Fig H). The same methodology as the one used for signature threshold selection in rRNA was used for the caspase composite alignment. The signature threshold selected for TwinCons calculated with structure inferred matrices for the caspase-metacaspase alignment was -0.9.

## *Supplementary Results*

**TwinCons detects sequence similarity between P-loop domains of EF-Tu and Initiation factor 2.**

First, we tested the ability of the automated TwinCons search algorithm to detect regions of high sequence similarity between the catalytic GTPase domains of two translation factors: initiation factor 2 (IF2 in bacteria; aIF5 in Archaea; PDB ID: 5YT0 [15]) and elongation factor thermo unstable (EF-Tu; PDB ID: 1EFC [16]). These domains are known to be paralogous; their duplication predates the last universal common ancestor [17, 18]. TwinCons detects 2 significant segments between archaeal sequences of aIF5 and bacterial sequences of EF-Tu (Fig 4, green circles) (Table 2). Highly significant segments detected by TwinCons cover the binding site for GTP (Fig J). HHalign finds strong homology between the entire structural core of the aIF5 and EF-Tu P-loop domain (E-value $3.6e^{-16}$). HHalign detects larger region as significant, totaling 233

residues, compared to the 70 residues in the two TwinCons segments that cover only the catalytic center of GTPase (Table 2).

**TwinCons detects sequence similarity between ribosomal proteins and RNA polymerase subunits.**

Archaeal RNAP (aRNAP) have additional protein chains when compared with bacterial RNAP [19, 20]. One of these additional proteins is Subunit E (Rpb7 in eukaryotes, referred here as aRNAP7). This subunit, positioned on the periphery of aRNAP (PDB ID: 4V8S [21]), shares an OB-fold structural topology with bacterial rProtein bS1 (PDB ID: 4V9D [22]). rProtein bS1 is formed by 6 OB-fold β-barrels labeled D1 through D6 [23, 24] and aRNAP7 is comprised of an N-terminal truncated RNP motif and a C-terminal OB-fold of the S1 motif [25, 26]. The S1 motif is commonly found in nucleic acid binding proteins [27], such as initiation factors [28-30], RNA helicases [31] and ribonuclease E [32]. We checked, if TwinCons are able to discover sequence similarity between bS1 and aRNAP7.

TwinCons detects a significant segment that covers the first three β-strands of the β-barrel in aRNAP7 (Fig K) and part of D3 from bS1. We were unable to map the TwinCons score on bS1 because there are no modelled structures of D3 for bS1. HHalign also detects a significant hit (Table 2) for the same region. Notably both D3 of bS1 and the β-barrel of aRNAP7 have roles in binding mRNA [24, 26, 33-35].

**TwinCons detects differences in sequence similarity between rProteins that have migrated on the ribosomal surface.**

Using a few non-trivial examples of possible ancestral relationship between 3 groups of ribosomal proteins, we further explore the predictions of the automated TwinCons search. The archaeal rProteins aL8, aL30, and eS12 exhibit promiscuous binding on the ribosomal surface [36] and share structural similarity together with eukaryotic rProteins [36]. This makes rProteins aL8, aL30, and eS12 prime candidates to detect sequence similarity. These rProteins belong to the α+β three layers topology and L7Ae family of folds [37, 38]. Sequence similarity between single representatives of aL8 and aL30 has already been reported [39] and their homology has been verified through shared gene clusters [40]. We used TwinCons and HHalign to search for sequence segments with significant similarity scores within composite alignments of aL8-aL30, aL8-eS12, and aL30-eS12.

TwinCons detects one significant segment in the aL8-eS12, aL8-aL30, aL30-eS12, composite alignments. (Fig 4, red, light green, and salmon circles; Table 2). The composite alignment of aL8 with eS12 has a longer segment, compared the segments for aL8-aL30 and aL30-eS12 (Table 2). The aL8-eS12 segment is also at a greater distance from the decision boundary than the segments from the other two composite alignments (Table 2).

The length of significant segments, identified with TwinCons, can be used as a proxy for similarity between the sequences of aL8, aL30, and eS12. Most similar are rProteins in the aL8-eS12 composite alignment with a segment with length of 63, next is the composite alignment of aL8-aL30 with 44, finally the aL30-eS12 composite alignment produces a segment with length of 40 (Table 2). HHalign results agree with these TwinCons calculations (Table 2). These results suggest that the eukaryotic specific rProtein eS12 was more likely formed through the duplication of aL8 and not aL30.

**Sequence similarity between rProteins with shared location bL34 and aL37.**

TwinCons detects sequence similarity between bL34 and aL37, a pair of rProteins that share structural location but exhibit different 2D and 3D structures. bL34 and aL37 are short (< 60 aa) rProteins, buried deep within the large subunit rRNA. bL34 is comprised of two short α-helices, while aL37 has a Zn-binding Rubredoxin-like topology with many loops and two short β-strands (Fig L). TwinCons detects a single segment with significant sequence similarity between the two groups (Fig 4, violet circle), pointing to a possible common ancestry. The segment covers an N-terminal loop in aL37 that has near α-helical conformation and an N-terminal α-helix within bL34 (Fig L). The segment length is 12 residues in aL37 and 13 residues in bL34 (Table 2). HHalign did not detect significant similarity between the two sequence groups (Table 2). The difference between TwinCons and HHalign results might stem from the short length of the studied proteins.

## Supplementary Figures

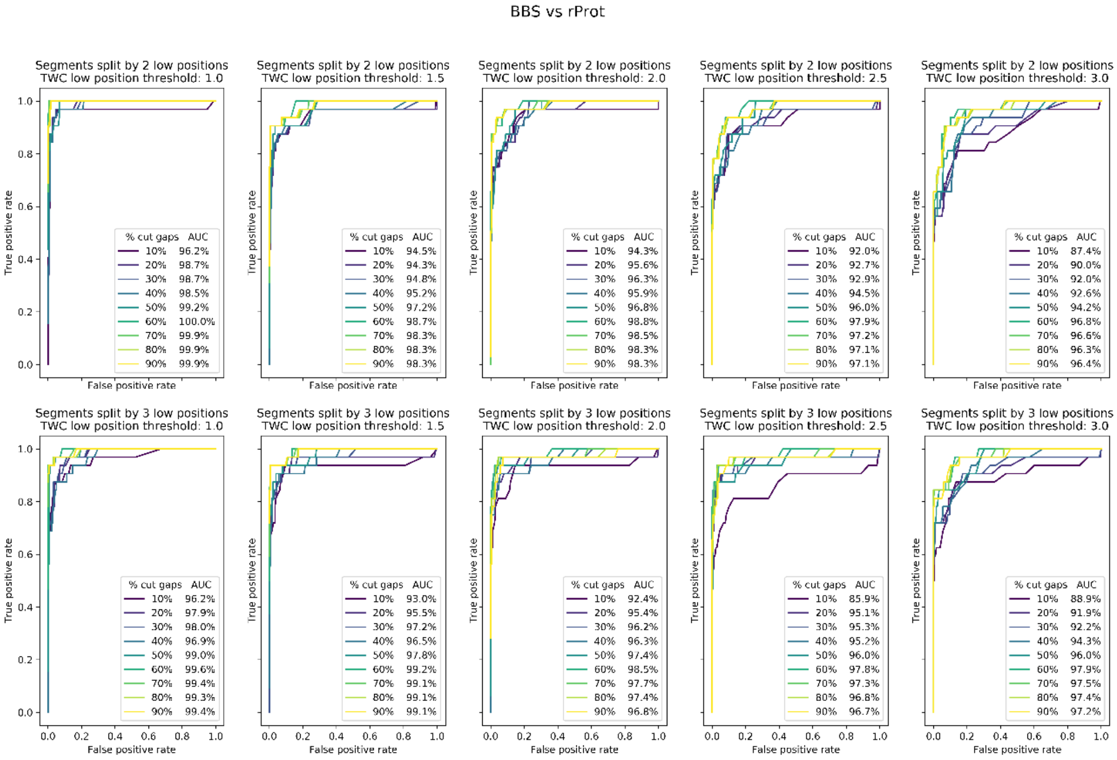


**Fig A.** ROC curves for classifiers with different parameters built from the BaliBASE dataset, tested against the rProt dataset. Parameters shown here are segment boundaries (length threshold), TWC intensity for detection of positive positions (intensity threshold), and what percentage gaps should be used for removal of alignment columns (gap threshold). Each subplot represents different combination of the intensity and length thresholds. Colored lines within subplots represent different gap thresholds. Cutting only alignment positions with more than 80-90% gaps produce better distinction between true positive and true negatives. Complete data including all tested parameters and datasets is available in S2 Dataset.

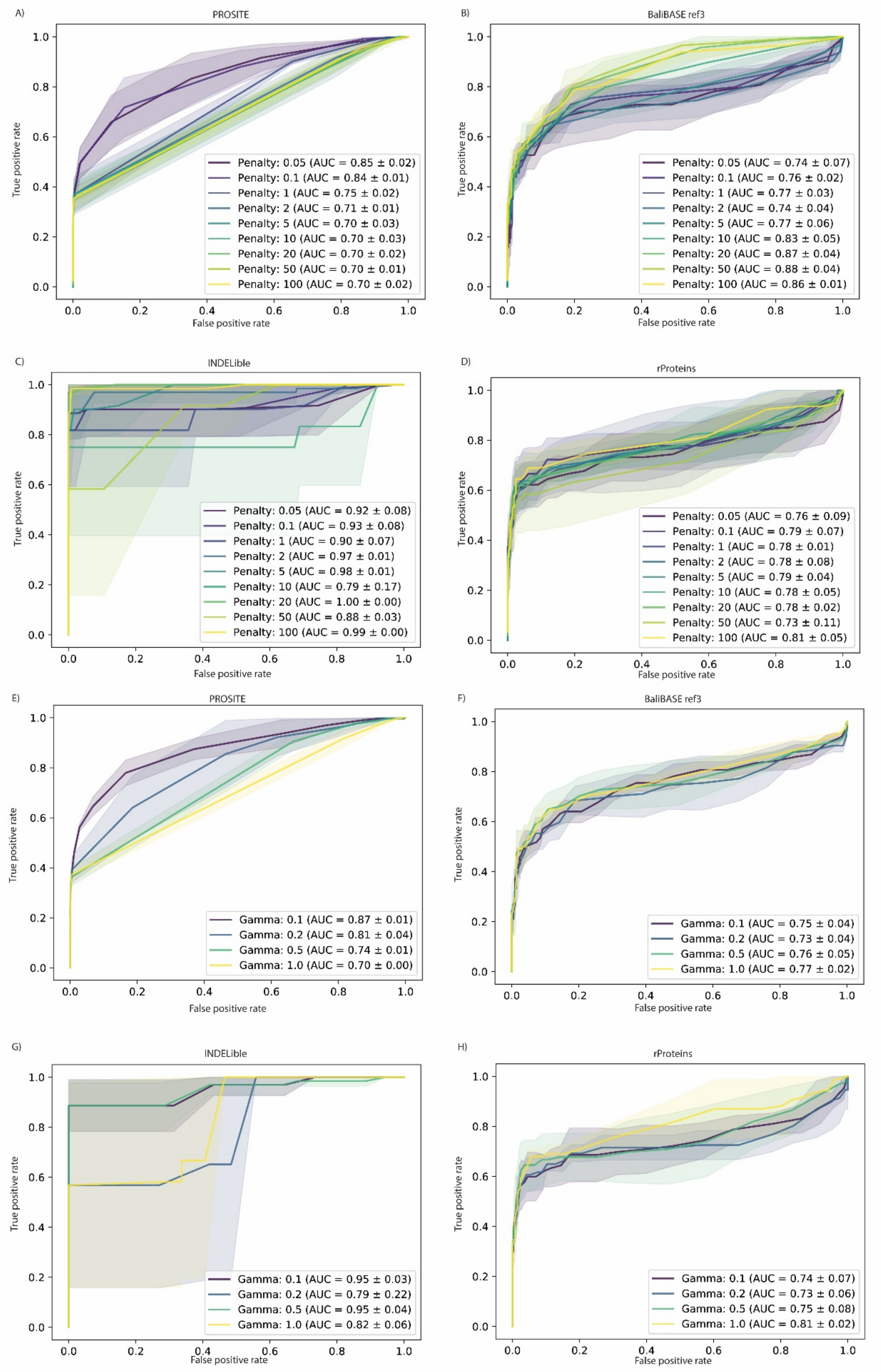
A)
PROSITE
True positive rate
False positive rate
Penalty: 0.05 (AUC = 0.85 ± 0.02)
Penalty: 0.1 (AUC = 0.84 ± 0.01)
Penalty: 1 (AUC = 0.75 ± 0.02)
Penalty: 2 (AUC = 0.71 ± 0.01)
Penalty: 5 (AUC = 0.70 ± 0.03)
Penalty: 10 (AUC = 0.70 ± 0.03)
Penalty: 20 (AUC = 0.70 ± 0.02)
Penalty: 50 (AUC = 0.70 ± 0.01)
Penalty: 100 (AUC = 0.70 ± 0.02)
B)
BaliBASE ref3
True positive rate
False positive rate
Penalty: 0.05 (AUC = 0.74 ± 0.07)
Penalty: 0.1 (AUC = 0.76 ± 0.02)
Penalty: 1 (AUC = 0.77 ± 0.03)
Penalty: 2 (AUC = 0.74 ± 0.04)
Penalty: 5 (AUC = 0.77 ± 0.06)
Penalty: 10 (AUC = 0.83 ± 0.05)
Penalty: 20 (AUC = 0.87 ± 0.04)
Penalty: 50 (AUC = 0.88 ± 0.04)
Penalty: 100 (AUC = 0.86 ± 0.01)
C)
INDELible
True positive rate
False positive rate
Penalty: 0.05 (AUC = 0.92 ± 0.08)
Penalty: 0.1 (AUC = 0.93 ± 0.08)
Penalty: 1 (AUC = 0.90 ± 0.07)
Penalty: 2 (AUC = 0.97 ± 0.01)
Penalty: 5 (AUC = 0.98 ± 0.01)
Penalty: 10 (AUC = 0.79 ± 0.17)
Penalty: 20 (AUC = 1.00 ± 0.00)
Penalty: 50 (AUC = 0.88 ± 0.03)
Penalty: 100 (AUC = 0.99 ± 0.00)
D)
rProteins
True positive rate
False positive rate
Penalty: 0.05 (AUC = 0.76 ± 0.09)
Penalty: 0.1 (AUC = 0.79 ± 0.07)
Penalty: 1 (AUC = 0.78 ± 0.01)
Penalty: 2 (AUC = 0.78 ± 0.08)
Penalty: 5 (AUC = 0.79 ± 0.04)
Penalty: 10 (AUC = 0.78 ± 0.05)
Penalty: 20 (AUC = 0.78 ± 0.02)
Penalty: 50 (AUC = 0.73 ± 0.11)
Penalty: 100 (AUC = 0.81 ± 0.05)
E)
PROSITE
True positive rate
False positive rate
Gamma: 0.1 (AUC = 0.87 ± 0.01)
Gamma: 0.2 (AUC = 0.81 ± 0.04)
Gamma: 0.5 (AUC = 0.74 ± 0.01)
Gamma: 1.0 (AUC = 0.70 ± 0.00)
F)
BaliBASE ref3
True positive rate
False positive rate
Gamma: 0.1 (AUC = 0.75 ± 0.04)
Gamma: 0.2 (AUC = 0.73 ± 0.04)
Gamma: 0.5 (AUC = 0.76 ± 0.05)
Gamma: 1.0 (AUC = 0.77 ± 0.02)
G)
INDELible
True positive rate
False positive rate
Gamma: 0.1 (AUC = 0.95 ± 0.03)
Gamma: 0.2 (AUC = 0.79 ± 0.22)
Gamma: 0.5 (AUC = 0.95 ± 0.04)
Gamma: 1.0 (AUC = 0.82 ± 0.06)
H)
rProteins
True positive rate
False positive rate
Gamma: 0.1 (AUC = 0.74 ± 0.07)
Gamma: 0.2 (AUC = 0.73 ± 0.06)
Gamma: 0.5 (AUC = 0.75 ± 0.08)
Gamma: 1.0 (AUC = 0.81 ± 0.02)

**Fig B**. ROC curves of training classifiers with different penalties and gamma parameters. The first four subplots (A-D) test different penalties and the last four test different gamma values (E-H). Each subplot represents a different dataset that was used for training and testing. (A) and (E) are PROSITE, (B) and (F) are BaliBASE, (C) and (G) are INDELible, (D) and (H) are rProtein dataset. For testing each dataset was split in 3 folds. Each fold produces an ROC curve, we plot the mean of the three results as single curve and plot the standard deviation of the true positive rate as a shaded region around it. Complete data is available in S3Dataset.

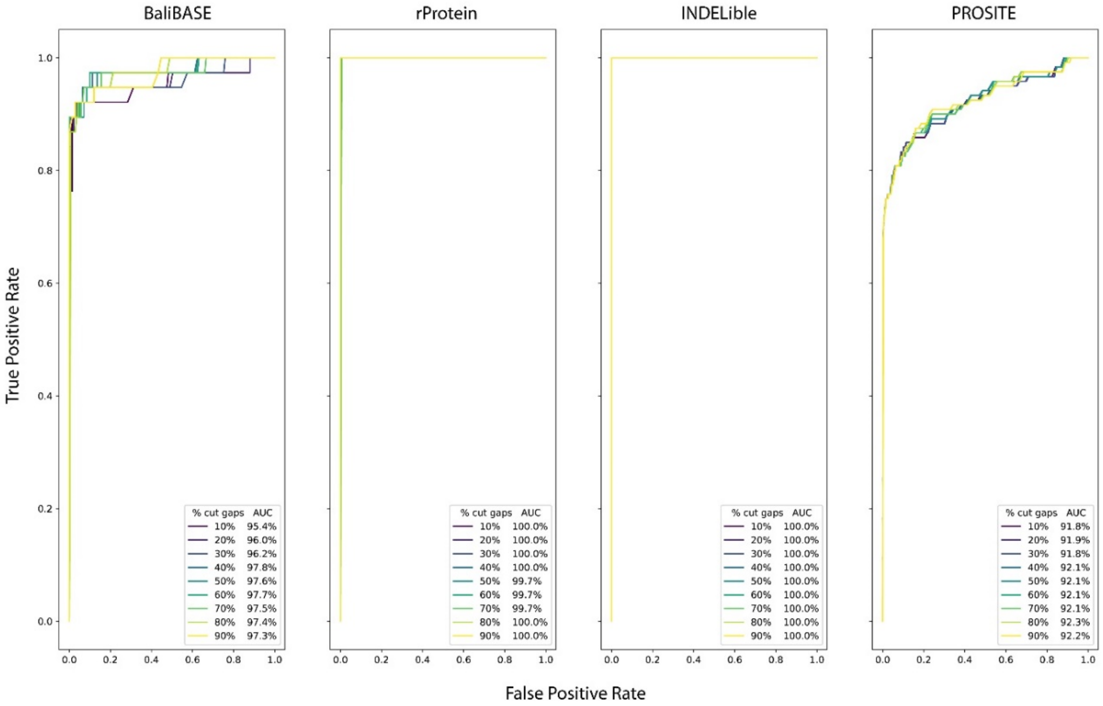


**Fig C**. ROC curves generated from HHalign alignments from the four datasets: BaliBASE, rProtein, INDELible, and PROSITE. Colored lines within subplots represent different gap thresholds used for column exclusion.

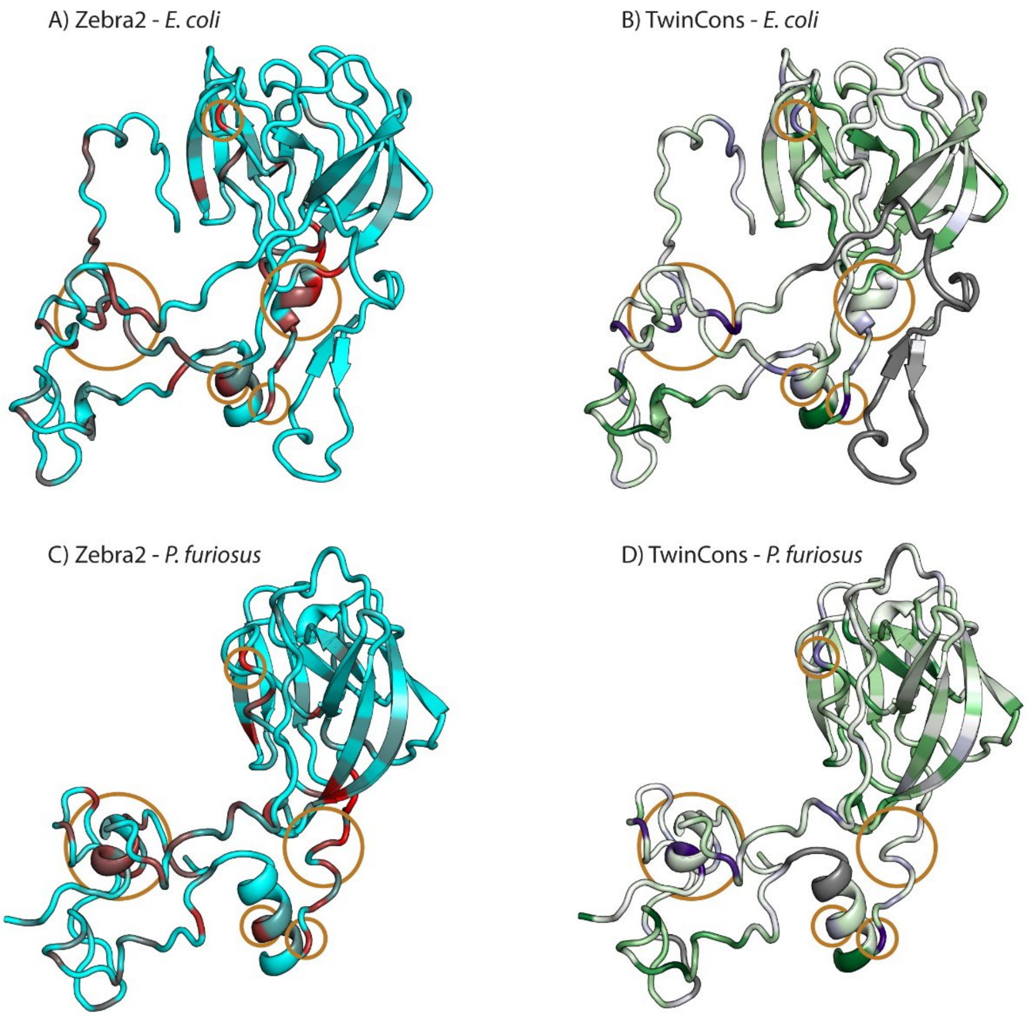


**Fig D**. Comparison of structural mapping between Zebra2 and TwinCons. A) Zebra2 results and B) TwinCons results from sequence alignment for uL02 between archaeal and bacterial sequences mapped on the *E. coli* uL02 structure from PDB 4V9D [22]. C) Zebra2 results and D) TwinCons results from the same sequence alignment mapped on the *P. furiosus* uL02 structure from PDB 4V6U [36]. In panels A) and C) red indicates signatures. In panels B) and D) dark green indicates alignment positions with high conservation of residues, purple indicates signature positions, gray indicates heavily gapped regions in the composite alignment. Orange circles indicate signature positions.

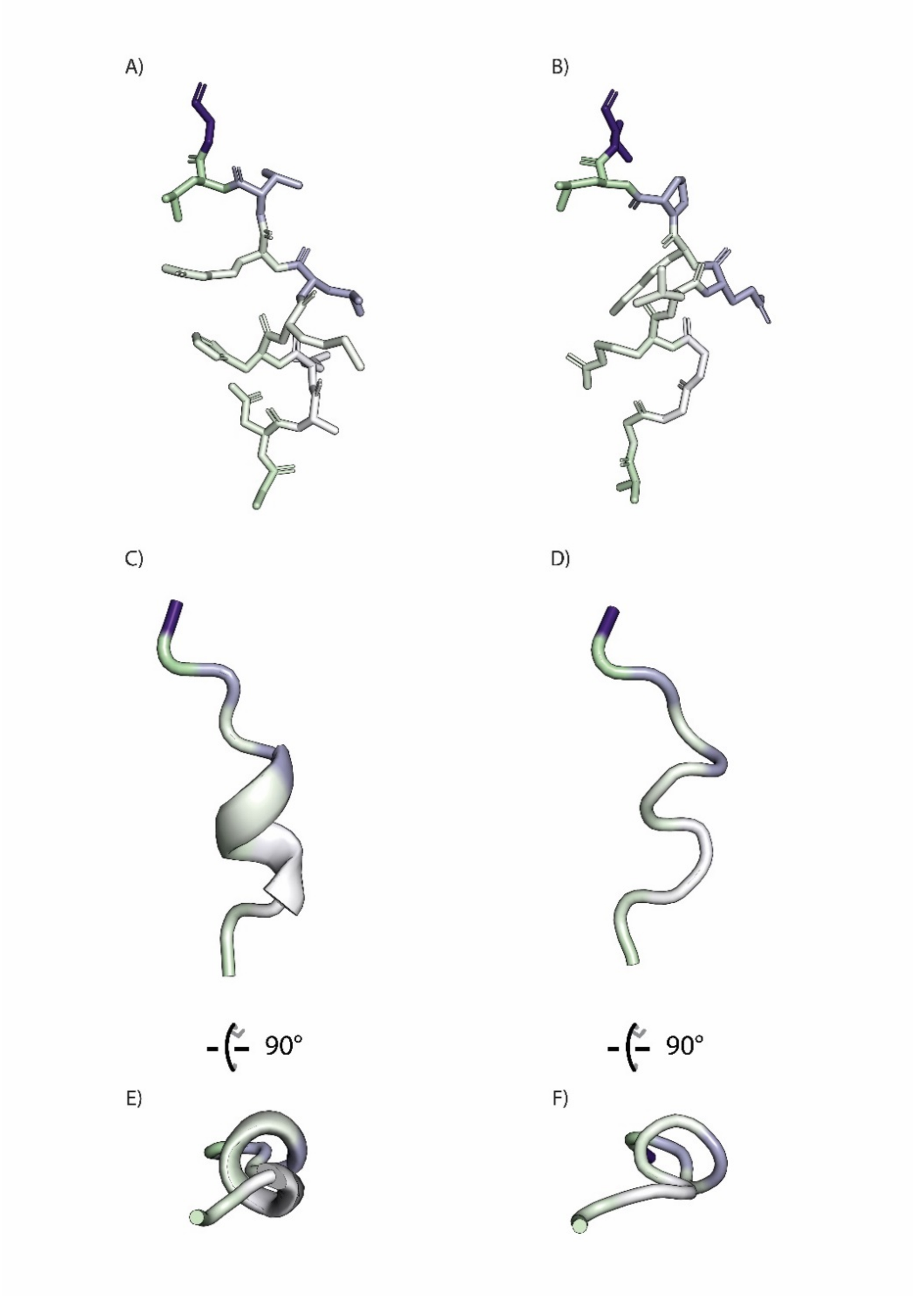


**Fig E**. TwinCons mapped for a short α-helix region in uL2 with analogous sequence between Bacteria and Archaea. Residues depicted here are listed in Table C. (A) stick representation for *E. coli* uL2. (B) stick representation for *P. furiosus* uL2. (C) cartoon representation of *E. coli* uL2. (D) cartoon representation of *P. furiosus* uL2. (E) and (F) show different angle for the *E. coli* and *P. furiosus* uL2. Conserved residues are colored green, signatures are colored purple, and random positions are white. Heavily gapped regions, present in a single group, are colored gray. Figure generated with PyMOL [41]. PDB IDs and chains used for the figure are available in Table E.

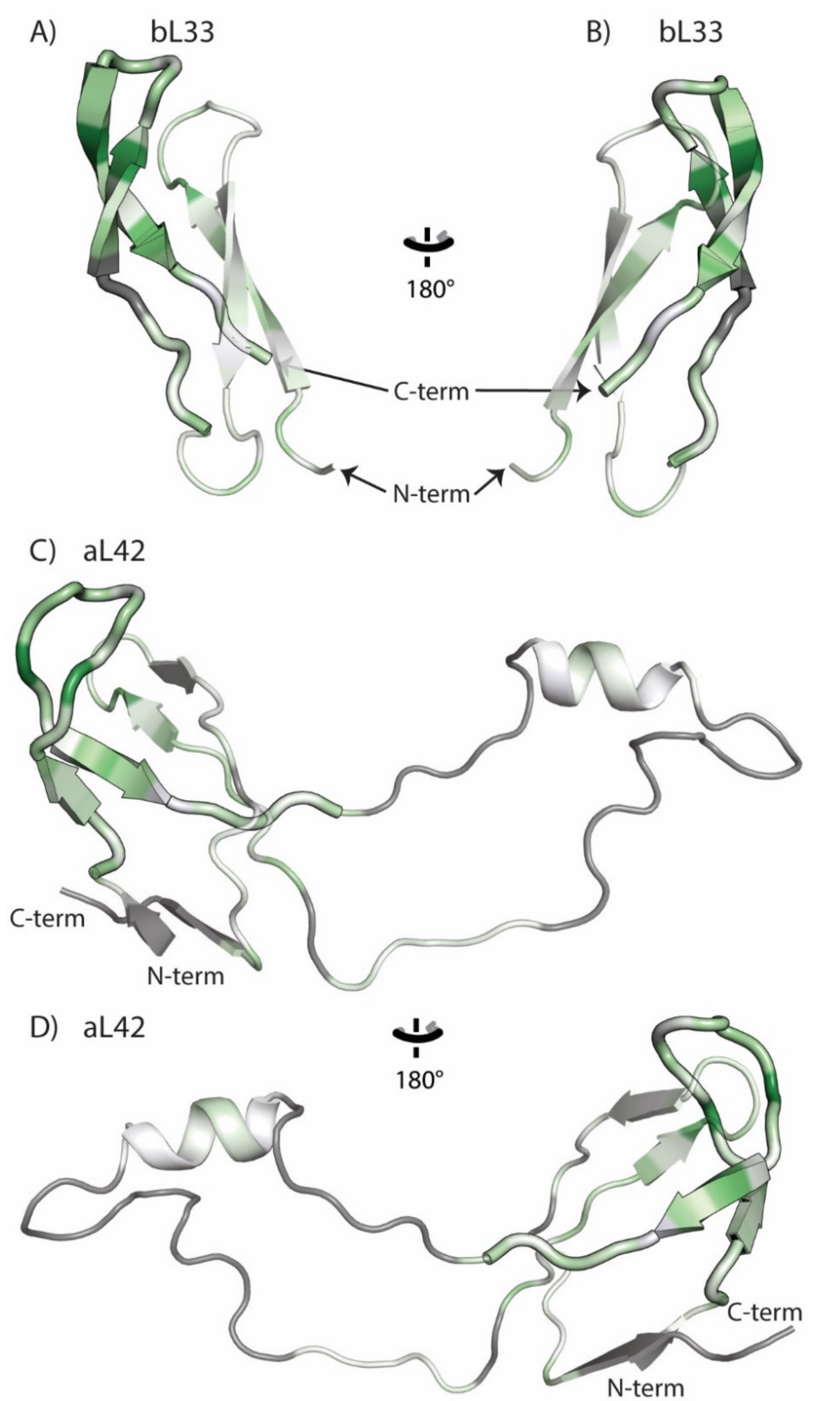


**Fig F.** TwinCons segment with significant sequence similarity between (A, B) bL33 and (C, D) aL42. The segment is shown with full opacity cartoon, non-segment regions are shown with transparent cartoon. Conserved residues are colored green, signatures are colored purple, and random positions are white. Heavily gapped regions, present in a single group, are colored gray. Segment definitions are available in S6 Dataset. Figure generated with PyMOL [41]. PDB IDs and chains used for the figure are available in Table E.

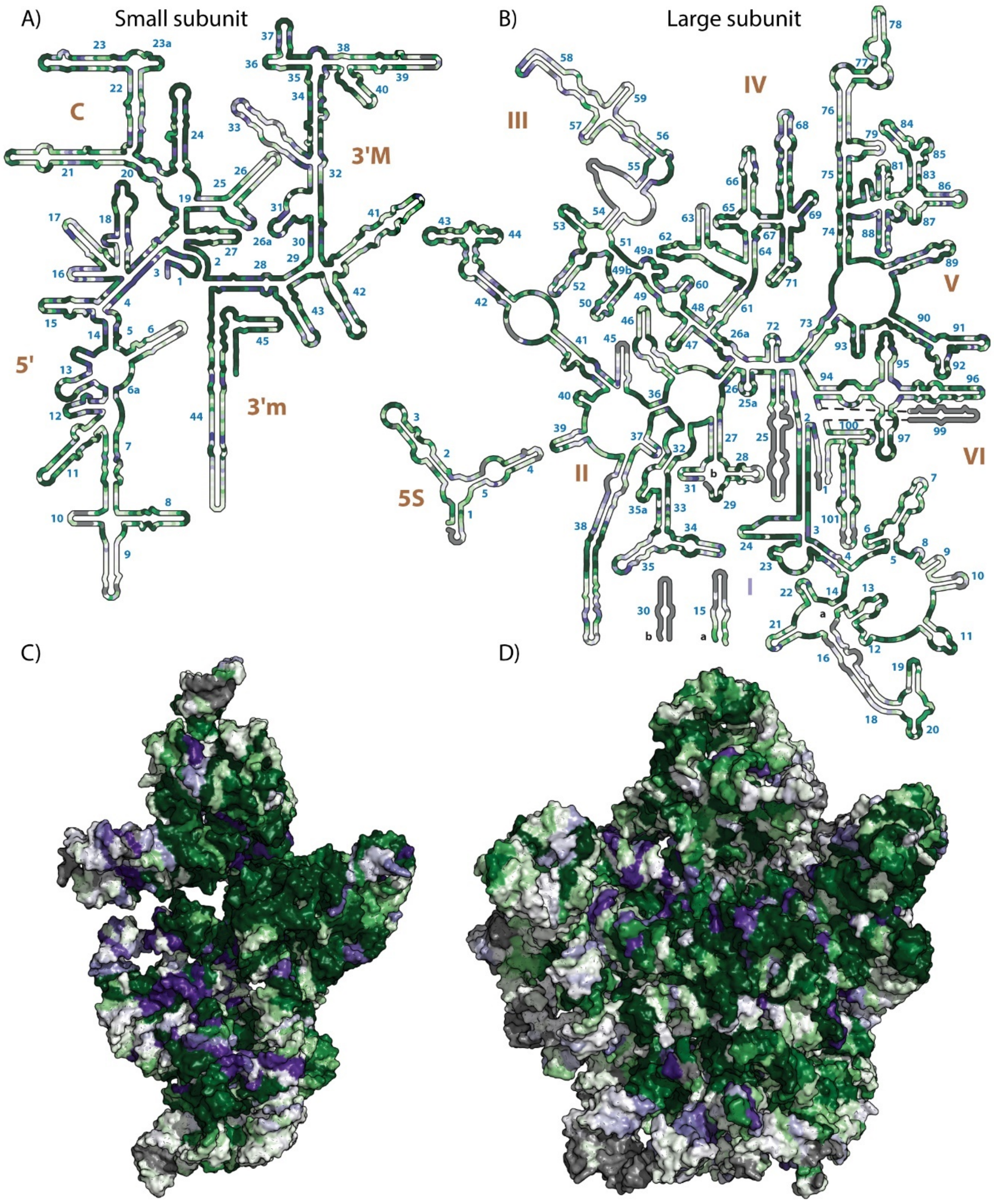
A)
Small subunit
B)
Large subunit
C
5'
3'M
3'm
III
IV
V
VI
II
I
5S
C)
D)

**Fig G.** TwinCons score for Archaea and Bacteria composite alignments of the small and large subunits. (A) Secondary structure of the *P. furiosus* 16S rRNA with mapped TwinCons. (B) Secondary structure of the *P. furiosus* 5S and 23S rRNAs with mapped TwinCons. (C) Surface representation of the 16S rRNA for *P. furiosus* ribosome. (D) Surface representation of the 5S and 23S rRNAs for *P. furiosus* ribosome in crown view. Both the small and large subunits are shown from the subunit interface direction. Gray indicates heavily gapped regions, present only in bacterial or archaeal sequences; dark green indicates highly conserved regions between both bacterial and archaeal sequences; dark purple indicates signature regions between bacterial and archaeal sequences; white indicates sequence variable regions. In panels (A) and (B) blue numbers indicate helical numbering and ribosomal domains are indicated with brown. Panels (A) and (B) are generated with RiboVision [42], panels (C) and (D) are generated with PyMOL [41]. PDB IDs and chains used for the figure are available in Table E.

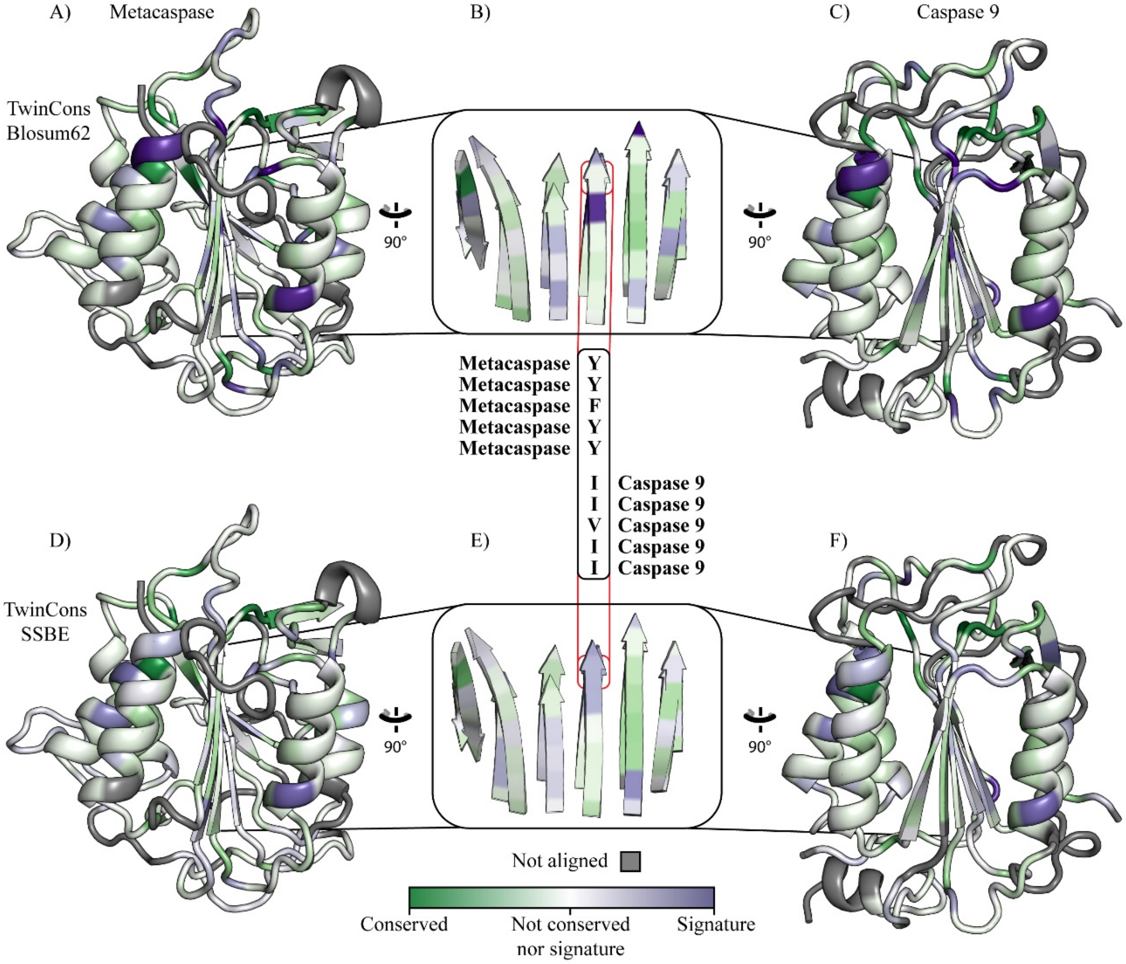


**Fig H.** TwinCons signatures differ based on the substitution matrix used. TwinCons results mapped on (A) metacaspase, (C) caspase, and (B) β-sheet superimposition of both structures, using the Blosum62 matrix. TwinCons results mapped on (D) metacaspase, (F) caspase, and (E) β-sheet superimposition of both structures, using structure-informed substitution matrices. A position with differing result is highlighted between panels (B) and (D) with red. Set of residues, representing the composite alignment column for the highlighted position, are shown between (B) and (E). Structure-informed matrices produce stronger signature signal between the two groups for this alignment position. Structures are generated with PyMOL [41]. PDB IDs and chains used for the figure are available in Table E.

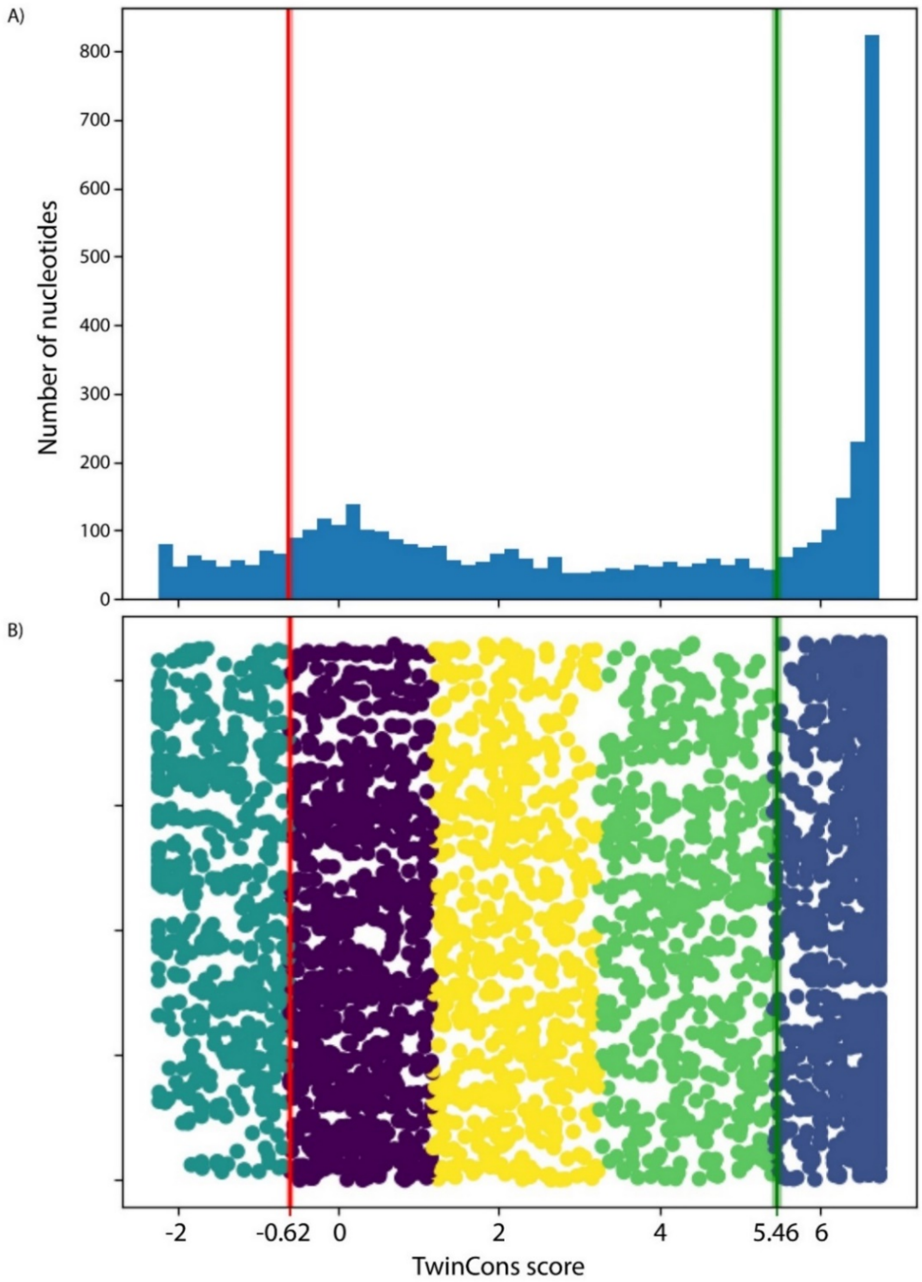


**Fig I.** Distribution of TwinCons scores from the E. coli rRNA, based on three composite alignments between Archaeal and Bacterial sequences of 23S, 16S, and 5S rRNA. (A) Histogram of TwinCons scores showing three peaks of distribution around the minimum score, score zero, and the maximum score. (B) Scatter plot of TwinCons scores with group assignment by k-means clustering algorithm. The y-axis holds randomly assigned values and is only illustrative. Scores from different groups are colored with the viridis gradient. The red and green lines indicate the calculated thresholds of the groups spanning the lowest (red) and highest (green) scores. Thresholds calculated from each composite alignment are available in Table B.

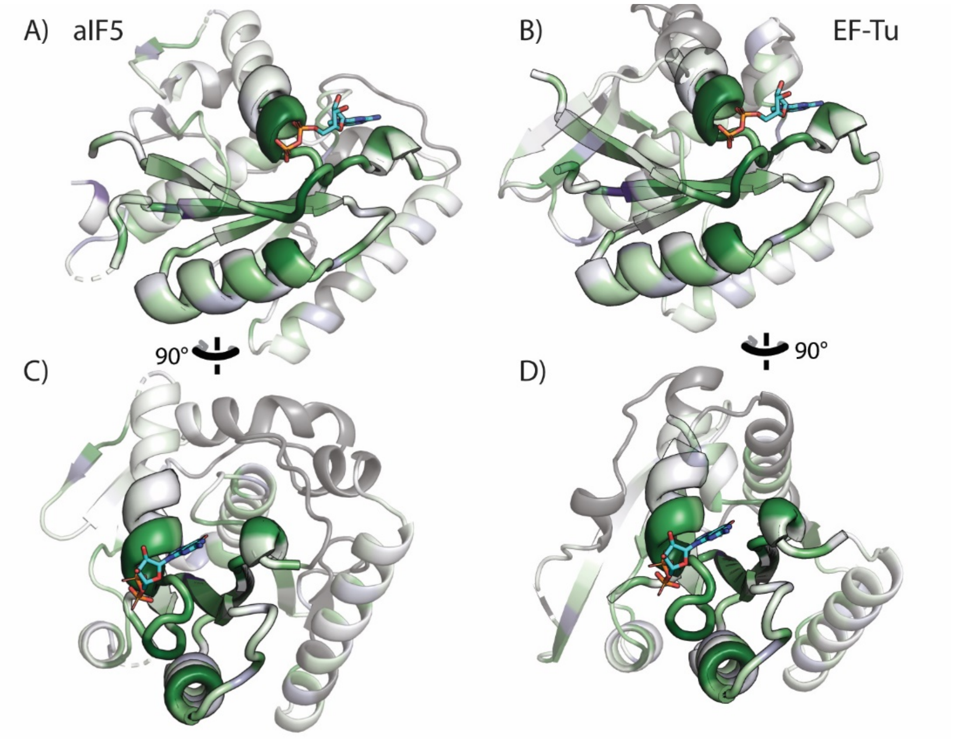


**Fig J**. TwinCons segments with significant sequence similarity between the P-loop domains of (A, C) aIF5 and (B, D) EF-Tu. Segments are shown with full opacity cartoon, while non-segment regions are shown with transparent cartoon. GDP from the EF-Tu structure is shown with sticks. Conserved residues are colored green, signatures are colored purple, and random positions are white. Heavily gapped regions, present in a single group, are colored gray. Segment definitions are available in S6 Dataset. Figure generated with PyMOL [41]. PDB IDs and chains used for the figure are available in Table E.

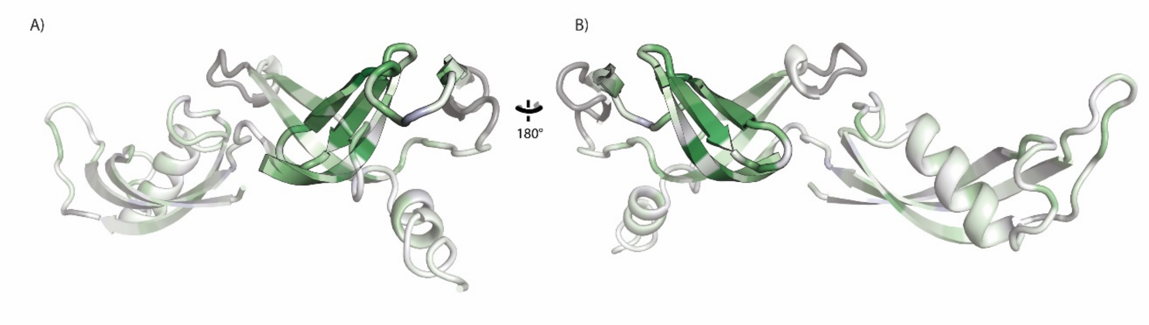


**Fig K.** TwinCons segment with significant sequence similarity between bS1 and domain 7 of RNAP mapped on the RNAP7 structure. (A) and (B) two views of the segment mapped on the RNAP7 structure. Segment is shown with full opacity cartoon, while non-segment regions are shown with transparent cartoon. Conserved residues are colored green, signatures are colored purple, and random positions are white. Heavily gapped regions, present in a single group, are colored gray. Segment definitions are available in S6 Dataset. Figure generated with PyMOL [41]. PDB IDs and chains used for the figure are available in Table E.

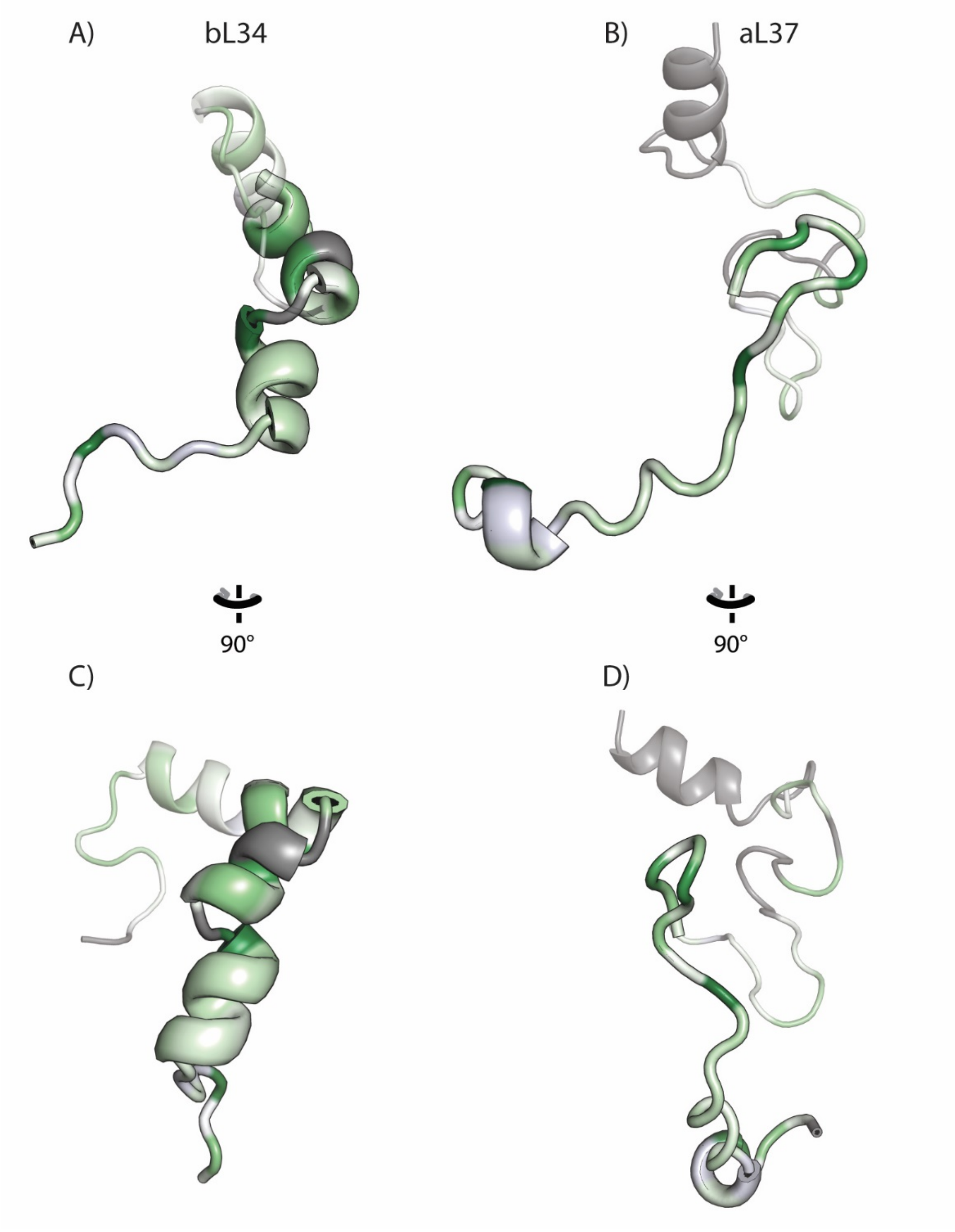


**Fig L.** TwinCons segment with significant sequence similarity between bL34 and aL37. (A) representation of *E. coli* bL34, (B) representation of *P. furiosus* aL37, (C) 90-degree rotation view of *E. coli* bL34, and (D) 90-degree rotation view of *P. furiosus* aL37. The segment is shown with full opacity cartoon, non-segment regions are shown with transparent cartoon. Conserved residues are colored green, signatures are colored purple, and random positions are white. Heavily gapped regions, present in a single group, are colored gray. Segment definitions are available in S6 Dataset. Figure generated with PyMOL [41]. PDB IDs and chains used for the figure are available in Table E.

## Supplementary Tables

**Table A.** Substitution matrices available for TwinCons calculation. Full descriptions of matrices are available in Vogt, Etzold (43) and in Le and Gascuel (14).

| Matrix | Description | Reference |
|---|---|---|
| B | Protein structure buried | [14] |
| BH | Protein structure buried helix | [14] |
| BO | Protein structure buried other | [14] |
| BS | Protein structure buried sheet | [14] |
| E | Protein structure exposed | [14] |
| EH | Protein structure exposed helix | [14] |
| EO | Protein structure exposed other | [14] |
| ES | Protein structure exposed sheet | [14] |
| H | Protein structure helix | [14] |
| O | Protein structure other | [14] |
| S | Protein structure sheet | [14] |
| BEHOS | LG matrix when structure does not match between the two groups | [14] |
| LG | Protein structure | [44] |
| WAG | Protein phylogeny maximum likelihood | [45] |
| blastn | nucleotide | |
| identity | nucleotide | |
| trans | nucleotide | |
| benner6, benner22, benner74 | Aligned database sequences clustered by pam distance | [46] |
| blosum30, blosum35, blosum40, blosum45, blosum50, blosum55, blosum60, blosum62, blosum65, blosum70, blosum75, blosum80, blosum85, blosum90, blosum95, blosum100 | Aligned sequence segments in family groups | [2] |
| genetic | Genetic code distance | [46] |

| | | |
|---|---|---|
| gonnet | Aligned and family clustered database sequences | [47] |
| ident | Exact residue conservation | |
| johnson | Family alignment by structural superposition | |
| levin | Secondary structural properties | [48] |
| miyata | Volume and polarity of amino acid types | [49] |
| nwsgappep | Modified Dayhoff pam250 matrix | [50] |
| pam30, pam60, pam90, pam180, pam250, pam300 | Evolutionary model for point mutations in ancestral families | [51] |
| risler | Pairwise alignments from protein structural superposition | [52] |
| structure | Structure based substitutions | [53] |

**Table B.** TwinCons thresholds calculated with 5 k-clusters for different subsets of rRNA. First two rows, tagged with 'ribosome', include sequences from the 23S, 5S, and 16S. Entries tagged with LSU include sequences from the 23S and 5S. Entries tagged with SSU include only rRNA from the 16S rRNA. TwinCons was calculated against the Archaea-Bacteria composite alignment of the rRNA. Standard deviations were calculated after repeating the calculation 100 times. Full script used to generate this data can be found at https://github.com/LDWLab/TWC_distribution.

| rRNA source | Signature threshold | Signature STD | Conserved threshold | Conserved STD |
|---|---|---|---|---|
| EC ribosome | -0.617 | 0.034 | 5.460 | 0.048 |
| PF ribosome | -0.620 | 0.029 | 5.449 | 0.049 |
| PF LSU | -0.418 | 0.059 | 5.556 | 0.036 |
| EC LSU | -0.419 | 0.054 | 5.556 | 0.037 |
| PF SSU | -0.753 | 0.009 | 5.439 | 0.019 |
| EC SSU | -0.752 | 0.012 | 5.437 | 0.019 |

**Table C.** TwinCons and ConSurf statistics for α-helical region in uL2. Positions with low Shannon entropy, low ConSurf score, and high TwinCons score are detected as highly conserved. Positions with TwinCons below -0.6 are detected as signature positions. Signature positions detected with TwinCons, that are detected as conserved by ConSurf are highlighted with blue.

| *E. coli* residue | Bacteria consensus | *P. furiosus* residue | Archaea consensus | ConSurf score | ConSurf color group | ConSurf confidence | TwinCons score | Shannon entropy |
|---|---|---|---|---|---|---|---|---|
| 196 | G | 163 | A | -0.869 | 8 | 8.7 | 2.002 | 1.52 |
| 197 | N | 164 | G | -1.224 | 9 | 9.9 | 1.165 | 1.13 |
| 198 | E | 165 | G | 0.735 | 3 | 4.2 | 0.003 | 2.75 |
| 199 | E | 166 | G | -0.766 | 7 | 8.7 | -0.416 | 1.76 |
| 200 | H | 167 | R | -0.514 | 7 | 7.6 | 1.137 | 2.21 |
| 201 | M | 168 | T | 2.370 | 1 | 2.1 | 0.426 | 3.66 |
| 202 | N | 169 | E | -0.845 | 8 | 8.7 | -1.437 | 1.99 |
| 203 | I | 170 | K | -0.298 | 6 | 7.6 | 0.836 | 2.55 |
| 204 | N | 171 | P | 0.112 | 5 | 6.4 | -1.362 | 2.52 |
| 205 | L | 172 | F | 0.704 | 3 | 4.2 | 2.526 | 2.47 |
| 206 | G | 173 | L | -0.314 | 6 | 7.5 | -3.277 | 1.89 |

**Table D.** Composite alignments used in sequence similarity analysis.

| Protein name | Used in composite alignments from S5 Dataset |
|---|---|
| Trp aatRNA | aatRNAS_Y-W.fa |
| Tyr aatRNA | aatRNAS_Y-W.fa |
| aL14 | aeL14-eL27.fa |
| aL30 | aL08-aL30.fa; aL30-eS12.fa; |
| aL37 | aL37-bL34.fa; uL02c-aL37.fa |
| aL8 | aL08-aL30.fa; aL08-eS12.fa |
| aS8 | uL14a-aS08.fa |
| bL27 | bL27CP-uL16a-43.fa; bL27CP-uL16b-31.fa; bL27-uL16a.fa; bL27-uL16b.fa |
| bL34 | aL37-bL34.fa; uL02b-bL34.fa |
| bS1 | C-struc_bS1-RNAP7Ca.fas; N-struc_bS01-RNAP7Ca.fas |
| bS18 | uL11a-bS18.fa; uL11b-bS18.fa |
| EF-Tu | IF2-EFTU_Ploop.fa; aIF5-bEFTU.fa; |
| eL27 | aeL14-eL27.fa |
| eS12 | aL08-eS12.fa; aL30-eS12.fa; |
| IF2/IF5B | IF2-EFTU_Ploop.fa; aIF5-bEFTU.fa; uL03a-aIF5.fa |
| RNAP7 | C-struc_bS1-RNAP7Ca.fas; N-struc_bS01-RNAP7Ca.fas |
| RNAPA | uL03a-aRNAPA.fa |
| RNAPA 2 | uL03a-aRNAPA2.fa |
| RNAPB | uL03a-aRNAPB-ClustalW.fa; |
| uL11 | uL11a-bS18.fa; uL11b-bS18.fa |
| uL14 | uL14a-aS08.fa |
| uL16 | bL27CP-uL16a-43.fa; bL27CP-uL16b-31.fa; bL27-uL16a.fa; bL27-uL16b.fa; |
| uL2 | uL02c-aL37.fa; |
| uL3 | uL03a-aRNAPA.fa; uL03a-aRNAPA2.fa; uL03a-aRNAPB-ClustalW.fa; |
| uL30 | uL30b-aL08.fa; uL30b-aL30.fa |
| uL33 | uL33_aperm_STRUC.fa; uL33_bperm_PRMS.fa; uL33_bperm_STRUC.fa; uL33_noperm.fa; |

**Table E.** Protein and rRNA structures used to map sequence similarity analysis. When multiple PDBs are used in a single row they are separated by a semicolon. When multiple chains are used from a single PDB they are separated by &.

| Protein name | PDB ID | Chains | Figures | Citation |
|---|---|---|---|---|
| IF5B | 5YT0 | A | J | Murakami, Singh (15) |
| EF-Tu | 1EFC | A | J | Song, Parsons (16) |
| aL37 | 4V6U | Bi | L | Armache, Anger (36) |
| bL34 | 4V9D | D2 | L | Dunkle, Wang (22) |
| RNAP7 | 4V8S | AT | K | Wojtas, Mogni (21) |
| uL2 | 4V9D; 4V6U | DC; BB | 2; D, E | Dunkle, Wang (22), Armache, Anger (36) |
| 23S & 5S rRNA | 4V9D; 4V6U | DA & DB; B1 & B3 | 5, 6, G, L | Dunkle, Wang (22), Armache, Anger (36) |
| uL33 | 4V9D; 4V6U | D1; Bj | 6, F | Dunkle, Wang (22), Armache, Anger (36) |
| Caspase 9 | 1JXQ | A | 3, H | Renatus, Stennicke (12) |
| Metacaspase | 4F6O | A | 3, H | Wong, Yan (13) |

**Supplementary Dataset Descriptions**

**S1 Dataset (separate file).** Table with BaliBASE alignment names with enzyme and EC annotations present in the alignment. Alignments with similar EC annotations are colored the same. Combinations between alignments that share color are excluded from dataset generation.

**S2 Dataset (separate file).** Figures with ROC curves for all tested parameters. The title of each figure indicates the training dataset and the tested dataset. For example, "BBS vs PROSITE" indicates that the training set was BaliBASE and it was tested against the PROSITE dataset. ROC labels of subplots and lines are the same as the ones used in Fig A.

**S3 Dataset (separate file).** Data used to generate figure S2. The title of each sheet indicates whether penalties or gamma values were tested. TPR, FPR, and TPR standard deviation for each of the datasets. Calculations were done for boundary distance thresholds varying from -20 to 20 with a step of 0.05.

**S4 Dataset (separate file).** Performance of trained classifier from the BaliBASE dataset with best parameter combination against itself and the three other datasets. TPR, TNR, and precision for boundary distance thresholds varying from -5 to 5 with a step of 0.1. The distance thresholds of 0.7 and 1.5 are highlighted.

**S5 Dataset (separate file).** Description of query composite alignments used in figure 4. Alignments are available at https://apollo2.chemistry.gatech.edu/TwinConsDatasets/.

**S6 Dataset (separate file).** TwinCons segment results for composite alignments used in figure 4. Each segment is identified with its alignment position and the distance it was from the decision boundary.

**S7 Dataset (separate file).** TwinCons results from rRNA composite alignment of 23S, 16S, and 5S sequences between Archaea and Bacteria. TwinCons results from protein composite alignments of caspase-metacaspase and uL2. Thresholds for signature and conserved positions for each alignment are indicated.

**S8 Dataset (separate file).** INDELible control file used to generate artificial sequence alignments from random sequence seeds, evolved under biological model.

**S9 Dataset (separate file).** Combined TwinCons and HHalign results for segments detected within the query alignment set. The file reports alignment group names, TwinCons scores and probability, TwinCons segment ranges, HHalign scores and probabilities, HHalign ranges, and index sequences used for the ranges.

**S10 Dataset (separate file).** Direct score comparison for the alignment of uL2 between TwinCons and Zebra2, as well as TwinCons and ConSurf.

**Supplementary Information References**


1. Yu Y-K, Altschul SF. The construction of amino acid substitution matrices for the comparison of proteins with non-standard compositions. Bioinformatics. 2004;21(7):902-11. doi: 10.1093/bioinformatics/bti070.

2. Henikoff S, Henikoff JG. Amino acid substitution matrices from protein blocks. Proc Natl Acad Sci USA. 1992;89(22):10915-9. Epub 1992/11/15. PubMed PMID: 1438297; PubMed Central PMCID: PMCPMC50453.

3. Yu Y-K, Wootton JC, Altschul SF. The compositional adjustment of amino acid substitution matrices. Proc Natl Acad Sci USA. 2003;100(26):15688-93. doi: 10.1073/pnas.2533904100.

4. Fletcher W, Yang Z. Indelible: A flexible simulator of biological sequence evolution. Mol Biol Evol. 2009;26(8):1879-88. doi: 10.1093/molbev/msp098.

5. Katoh K, Misawa K, Kuma Ki, Miyata T. Mafft: A novel method for rapid multiple sequence alignment based on fast fourier transform. Nucleic Acids Res. 2002;30(14):3059-66. doi: 10.1093/nar/gkf436.

6. Katoh K, Standley DM. Mafft multiple sequence alignment software version 7: Improvements in performance and usability. Mol Biol Evol. 2013;30(4):772-80. doi: 10.1093/molbev/mst010.

7. Bernier CR, Petrov AS, Kovacs NA, Penev PI, Williams LD. Translation: The universal structural core of life. Mol Biol Evol. 2018;35(8):2065-76.

8. Thompson JD, Koehl P, Ripp R, Poch O. Balibase 3.0: Latest developments of the multiple sequence alignment benchmark. Proteins: Struct Funct Bioinform. 2005;61(1):127-36. doi: 10.1002/prot.20527.

9. Sigrist CJA, Cerutti L, Hulo N, Gattiker A, Falquet L, Pagni M, Bairoch A, Bucher P. Prosite: A documented database using patterns and profiles as motif descriptors. Briefings in Bioinformatics. 2002;3(3):265-74. doi: 10.1093/bib/3.3.265.

10. Sigrist CJ, de Castro E, Cerutti L, Cuche BA, Hulo N, Bridge A, Bougueleret L, Xenarios I. New and continuing developments at prosite. Nucleic Acids Res. 2013;41(Database issue):D344-7. Epub 2012/11/20. doi: 10.1093/nar/gks1067. PubMed PMID: 23161676; PubMed Central PMCID: PMCPMC3531220.

11. Virtanen P, Gommers R, Oliphant TE, Haberland M, Reddy T, Cournapeau D, Burovski E, Peterson P, Weckesser W, Bright J, van der Walt SJ, Brett M, Wilson J, Millman KJ, et al. Scipy 1.0: Fundamental algorithms for scientific computing in python. Nat Methods. 2020;17(3):261-72. doi: 10.1038/s41592-019-0686-2.

12. Renatus M, Stennicke HR, Scott FL, Liddington RC, Salvesen GS. Dimer formation drives the activation of the cell death protease caspase 9. Proc Natl Acad Sci USA. 2001;98(25):14250. doi: 10.1073/pnas.231465798.

13. Wong AH-H, Yan C, Shi Y. Crystal structure of the yeast metacaspase yca1. The Journal of biological chemistry. 2012;287(35):29251-9. Epub 2012/07/02. doi:

10.1074/jbc.M112.381806. PubMed PMID: 22761449.

14. Le SQ, Gascuel O. Accounting for solvent accessibility and secondary structure in protein phylogenetics is clearly beneficial. Syst Biol. 2010;59(3):277-87. Epub 2010/06/09. doi: 10.1093/sysbio/syq002. PubMed PMID: 20525635.

15. Murakami R, Singh CR, Morris J, Tang L, Harmon I, Takasu A, Miyoshi T, Ito K, Asano K, Uchiumi T. The interaction between the ribosomal stalk proteins and translation initiation factor 5b promotes translation initiation. Mol Cell Biol. 2018;38(16):e00067-18. doi: 10.1128/MCB.00067-18.

16. Song H, Parsons MR, Rowsell S, Leonard G, Phillips SEV. Crystal structure of intact elongation factor ef-tu from escherichia coli in gdp conformation at 2.05å resolution. J Mol Biol. 1999;285(3):1245-56. doi: https://doi.org/10.1006/jmbi.1998.2387.

17. Leipe DD, Wolf YI, Koonin EV, Aravind L. Classification and evolution of p-loop gtpases and related atpases. J Mol Biol. 2002;317(1):41-72.

18. Hartman H, Smith TF. Gtpases and the origin of the ribosome. Biol Direct. 2010;5:36. Epub 2010/05/22. doi: 10.1186/1745-6150-5-36. PubMed PMID: 20487556; PubMed Central PMCID: PMCPMC2881122.

19. Hirata A, Klein BJ, Murakami KS. The x-ray crystal structure of rna polymerase from archaea. Nature. 2008;451(7180):851-4. Epub 2008/02/01. doi: 10.1038/nature06530. PubMed PMID: 18235446; PubMed Central PMCID: PMCPMC2805805.

20. Jun SH, Reichlen MJ, Tajiri M, Murakami KS. Archaeal rna polymerase and transcription regulation. Crit Rev Biochem Mol Biol. 2011;46(1):27-40. Epub 2011/01/22. doi: 10.3109/10409238.2010.538662. PubMed PMID: 21250781; PubMed Central PMCID: PMCPMC3076279.

21. Wojtas MN, Mogni M, Millet O, Bell SD, Abrescia NGA. Structural and functional analyses of the interaction of archaeal rna polymerase with DNA. Nucleic Acids Res. 2012;40(19):9941-52. doi: 10.1093/nar/gks692.

22. Dunkle JA, Wang L, Feldman MB, Pulk A, Chen VB, Kapral GJ, Noeske J, Richardson JS, Blanchard SC, Cate JHD. Structures of the bacterial ribosome in classical and hybrid states of trna binding. Science. 2011;332(6032):981-4. doi: 10.1126/science.1202692.

23. Beckert B, Turk M, Czech A, Berninghausen O, Beckmann R, Ignatova Z, Plitzko JM, Wilson DN. Structure of a hibernating 100s ribosome reveals an inactive conformation of the ribosomal protein s1. Nat Microbiol. 2018;3(10):1115-21. doi: 10.1038/s41564-018-0237-0.

24. Subramanian AR. Structure and functions of ribosomal protein s1. Prog Nucleic Acid Res Mol Biol. 1983;28:101-42. Epub 1983/01/01. doi: 10.1016/s0079-6603(08)60085-9. PubMed PMID: 6348874.

25. Todone F, Brick P, Werner F, Weinzierl ROJ, Onesti S. Structure of an archaeal homolog of the eukaryotic rna polymerase ii rpb4/rpb7 complex. Mol Cell. 2001;8(5):1137-43. doi: 10.1016/S1097-2765(01)00379-3.

26. Meka H, Werner F, Cordell SC, Onesti S, Brick P. Crystal structure and rna binding of the rpb4/rpb7 subunits of human rna polymerase ii. Nucleic Acids Res.

2005;33(19):6435-44. doi: 10.1093/nar/gki945. PubMed PMID: 16282592.

27. Bycroft M, Hubbard TJP, Proctor M, Freund SMV, Murzin AG. The solution structure of the s1 rna binding domain: A member of an ancient nucleic acid–binding fold. Cell. 1997;88(2):235-42. doi: https://doi.org/10.1016/S0092-8674(00)81844-9.

28. Sette M, van Tilborg P, Spurio R, Kaptein R, Paci M, Gualerzi CO, Boelens R. The structure of the translational initiation factor if1 from e.Coli contains an oligomer-binding motif. EMBO J. 1997;16(6):1436-43. doi: 10.1093/emboj/16.6.1436. PubMed PMID: 9135158.

29. Battiste JL, Pestova TV, Hellen CUT, Wagner G. The eif1a solution structure reveals a large rna-binding surface important for scanning function. Mol Cell. 2000;5(1):109-19. doi: https://doi.org/10.1016/S1097-2765(00)80407-4.

30. Gribskov M. Translational initiation factors if-1 and eif-2 alpha share an rna-binding motif with prokaryotic ribosomal protein s1 and polynucleotide phosphorylase. Gene. 1992;119(1):107-11. Epub 1992/09/21. doi: 10.1016/0378-1119(92)90073-x. PubMed PMID: 1383091.

31. Company M, Arenas J, Abelson J. Requirement of the rna helicase-like protein prp22 for release of messenger rna from spliceosomes. Nature. 1991;349(6309):487-93. doi: 10.1038/349487a0.

32. Kaberdin VR, Miczak A, Jakobsen JS, Lin-Chao S, McDowall KJ, von Gabain A. The endoribonucleolytic n-terminal half of <em>escherichia coli</em> rnase e is evolutionarily conserved in <em>synechocystis</em> sp. And other bacteria but not the c-terminal half, which is sufficient for degradosome assembly. Proc Natl Acad Sci USA. 1998;95(20):11637. doi: 10.1073/pnas.95.20.11637.

33. Qu X, Lancaster L, Noller HF, Bustamante C, Tinoco I. Ribosomal protein s1 unwinds double-stranded rna in multiple steps. Proc Natl Acad Sci USA. 2012;109(36):14458. doi: 10.1073/pnas.1208950109.

34. Orlicky SM, Tran PT, Sayre MH, Edwards AM. Dissociable rpb4-rpb7 subassembly of rna polymerase ii binds to single-strand nucleic acid and mediates a post-recruitment step in transcription initiation. J Biol Chem. 2001;276(13):10097-102. Epub 2000/12/03. doi: 10.1074/jbc.M003165200. PubMed PMID: 11087726.

35. Duval M, Korepanov A, Fuchsbauer O, Fechter P, Haller A, Fabbretti A, Choulier L, Micura R, Klaholz BP, Romby P, Springer M, Marzi S. Escherichia coli ribosomal protein s1 unfolds structured mrnas onto the ribosome for active translation initiation. PLoS Biol. 2013;11(12):e1001731. Epub 2013/12/18. doi: 10.1371/journal.pbio.1001731. PubMed PMID: 24339747; PubMed Central PMCID: PMCPMC3858243.

36. Armache JP, Anger AM, Marquez V, Franckenberg S, Frohlich T, Villa E, Berninghausen O, Thomm M, Arnold GJ, Beckmann R, Wilson DN. Promiscuous behaviour of archaeal ribosomal proteins: Implications for eukaryotic ribosome evolution. Nucleic Acids Res. 2013;41(2):1284-93. Epub 2012/12/12. doi: 10.1093/nar/gks1259. PubMed PMID: 23222135; PubMed Central PMCID: PMCPMC3553981.

37. Cheng H, Schaeffer RD, Liao Y, Kinch LN, Pei J, Shi S, Kim B-H, Grishin NV. Ecod: An evolutionary classification of protein domains. PLoS Comp Biol.

2014;10(12):e1003926-e. doi: 10.1371/journal.pcbi.1003926. PubMed PMID: 25474468.

38. Cheng H, Liao Y, Schaeffer RD, Grishin NV. Manual classification strategies in the ecod database. Proteins. 2015;83(7):1238-51. Epub 2015/04/29. doi: 10.1002/prot.24818. PubMed PMID: 25917548; PubMed Central PMCID: PMCPMC4624060.

39. Chao JA, Prasad GS, White SA, Stout CD, Williamson JR. Inherent protein structural flexibility at the rna-binding interface of l30e. J Mol Biol. 2003;326(4):999-1004. doi: https://doi.org/10.1016/S0022-2836(02)01476-6.

40. Wang J, Dasgupta I, Fox GE. Many nonuniversal archaeal ribosomal proteins are found in conserved gene clusters. Archaea. 2009;2:971494. doi: 10.1155/2009/971494.

41. Schrodinger, LLC. The pymol molecular graphics system, version 1.8. 2015.

42. Bernier C, Petrov AS, Waterbury C, Jett J, Li F, Freil LE, Xiong b, Wang L, Le A, Milhouse BL, Hershkovitz E, Grover M, Xue Y, Hsiao C, et al. Ribovision: Visualization and analysis of ribosomes. Faraday Discuss. 2014;169(1):195-207. doi: 10.1039/C3FD00126A.

43. Vogt G, Etzold T, Argos P. An assessment of amino acid exchange matrices in aligning protein sequences: The twilight zone revisited. J Mol Biol. 1995;249(4):816-31. doi: https://doi.org/10.1006/jmbi.1995.0340.

44. Le SQ, Gascuel O. An improved general amino acid replacement matrix. Mol Biol Evol. 2008;25(7):1307-20. Epub 2008/03/28. doi: 10.1093/molbev/msn067. PubMed PMID: 18367465.

45. Whelan S, Goldman N. A general empirical model of protein evolution derived from multiple protein families using a maximum-likelihood approach. Mol Biol Evol. 2001;18(5):691-9. Epub 2001/04/25. doi: 10.1093/oxfordjournals.molbev.a003851. PubMed PMID: 11319253.

46. Benner SA, Cohen MA, Gonnet GH. Amino acid substitution during functionally constrained divergent evolution of protein sequences. Protein Eng. 1994;7(11):1323-32. Epub 1994/11/01. doi: 10.1093/protein/7.11.1323. PubMed PMID: 7700864.

47. Gonnet GH, Cohen MA, Benner SA. Exhaustive matching of the entire protein sequence database. Science. 1992;256(5062):1443-5.

48. Levin JM, Robson B, Garnier J. An algorithm for secondary structure determination in proteins based on sequence similarity. FEBS Lett. 1986;205(2):303-8. doi: https://doi.org/10.1016/0014-5793(86)80917-6.

49. Miyata T, Miyazawa S, Yasunaga T. Two types of amino acid substitutions in protein evolution. J Mol Evol. 1979;12(3):219-36. doi: 10.1007/BF01732340.

50. Gribskov M, Burgess RR. Sigma factors from e. Coli, b. Subtilis , phage sp01, and phage t4 are homologous proteins. Nucleic Acids Res. 1986;14(16):6745-63. doi: 10.1093/nar/14.16.6745.

51. Dayhoff MO, Barker WC, Hunt LT. [47] establishing homologies in protein sequences. Methods enzymol. 91: Academic Press; 1983. p. 524-45.

52. Risler JL, Delorme MO, Delacroix H, Henaut A. Amino acid substitutions in structurally related proteins a pattern recognition approach: Determination of a new and efficient

scoring matrix. J Mol Biol. 1988;204(4):1019-29. doi: https://doi.org/10.1016/0022-2836(88)90058-7.

53. Henikoff S, Henikoff JG. Performance evaluation of amino acid substitution matrices. Proteins. 1993;17(1):49-61. Epub 1993/09/01. doi: 10.1002/prot.340170108. PubMed PMID: 8234244.
