# Supplementary material for: TwinCons: Conservation score for uncovering deep sequence similarity and divergence
Source: PLoS Comput Biol. 2021 Oct 29;17(10):e1009541. doi: 10.1371/journal.pcbi.1009541 (PMC8580257; doi:10.1371/journal.pcbi.1009541)
Supplement: S2 Dataset — The title of each figure indicates the training dataset and the tested dataset. For example, “BBS vs PROSITE” indicates that the training set was BaliBASE and it was tested against the PROSITE dataset. ROC labels of subplots and lines are the same as the ones used in Fig A in S1 Appendix. (PDF) [file pcbi.1009541.s003.pdf]

TwinCons S2 Dataset

This dataset is a PDF generated with code available at https://github.com/LDWLab/TwinCons .

Each page of the PDF shows parameter testing of classifiers based on two datasets (BBS and PRST) against 4 datasets (BBS, PRST, rProt, and IND), excluding self-testing (e.g. BBS vs BBS).

Complete description of the datasets and parameters is available in the main manuscript text.

Titles indicate which is the dataset used for training and which is the dataset used for testing, separated by a dash.

The titles end with a descriptor for the general parameters used:

Cumulative - SVM classifiers, segments are created based on cumulative scoring by varying the window smoothing;

Old segment - SVM classifiers, segments are created based on intensity and length thresholds;

Trees - Random forest classifiers, segments are created based on cumulative scoring by varying the window smoothing.

For example, BBS-IND Cumulative shows parameters searched for classifier trained on BBS, tested against IND and using the cumulative segment creation rule.

Page to title index:

2 BBS-IND Cumulative
3 BBS-IND Old segments
4 BBS-IND Trees
5 BBS-PRST Cumulative
6 BBS-PRST Old segments
7 BBS-PRST Trees
8 BBS-rProt Cumulative
9 BBS-rProt Old segments
10 BBS-rProt Trees
11 PRST-BBS Cumulative
12 PRST-BBS Old segments
13 PRST-BBS Trees
14 PRST-IND Cumulative
15 PRST-IND Old segments
16 PRST-IND Trees
17 PRST-rProt Cumulative
18 PRST-rProt Old segments
19 PRST-rProt Trees

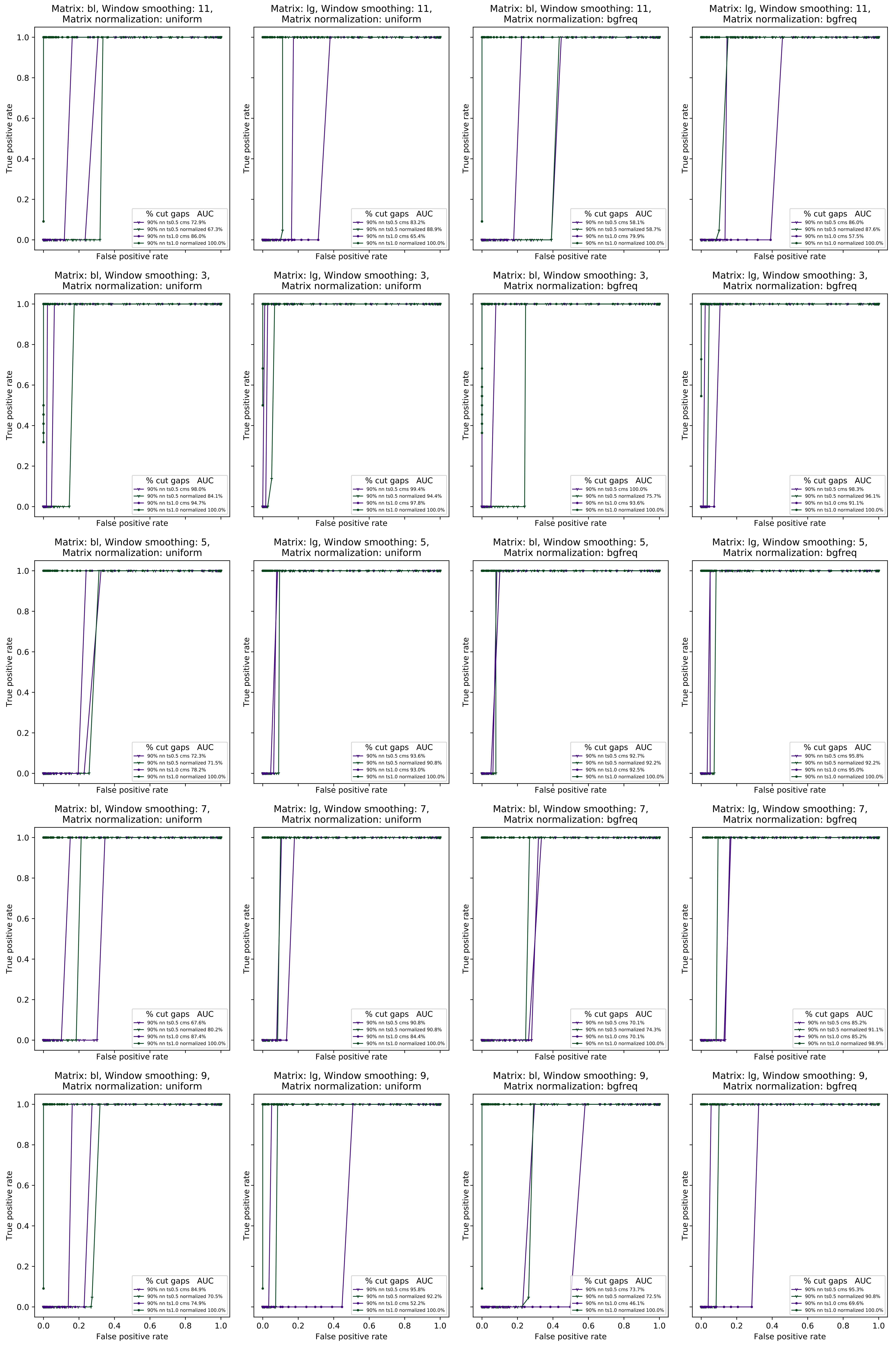

Matrix: bl, Window smoothing: 11, Matrix normalization: uniform
% cut gaps AUC
90% nn ts0.5 cms 72.9%
90% nn ts0.5 normalized 67.3%
90% nn ts1.0 cms 86.0%
90% nn ts1.0 normalized 100.0%
Matrix: lg, Window smoothing: 11, Matrix normalization: uniform
% cut gaps AUC
90% nn ts0.5 cms 83.2%
90% nn ts0.5 normalized 88.9%
90% nn ts1.0 cms 65.4%
90% nn ts1.0 normalized 100.0%
Matrix: bl, Window smoothing: 11, Matrix normalization: bgfreq
% cut gaps AUC
90% nn ts0.5 cms 58.1%
90% nn ts0.5 normalized 58.7%
90% nn ts1.0 cms 79.9%
90% nn ts1.0 normalized 100.0%
Matrix: lg, Window smoothing: 11, Matrix normalization: bgfreq
% cut gaps AUC
90% nn ts0.5 cms 86.0%
90% nn ts0.5 normalized 87.6%
90% nn ts1.0 cms 57.5%
90% nn ts1.0 normalized 100.0%
Matrix: bl, Window smoothing: 3, Matrix normalization: uniform
% cut gaps AUC
90% nn ts0.5 cms 98.0%
90% nn ts0.5 normalized 84.1%
90% nn ts1.0 cms 94.7%
90% nn ts1.0 normalized 100.0%
Matrix: lg, Window smoothing: 3, Matrix normalization: uniform
% cut gaps AUC
90% nn ts0.5 cms 99.4%
90% nn ts0.5 normalized 94.4%
90% nn ts1.0 cms 97.8%
90% nn ts1.0 normalized 100.0%
Matrix: bl, Window smoothing: 3, Matrix normalization: bgfreq
% cut gaps AUC
90% nn ts0.5 cms 100.0%
90% nn ts0.5 normalized 75.7%
90% nn ts1.0 cms 93.6%
90% nn ts1.0 normalized 100.0%
Matrix: lg, Window smoothing: 3, Matrix normalization: bgfreq
% cut gaps AUC
90% nn ts0.5 cms 98.3%
90% nn ts0.5 normalized 96.1%
90% nn ts1.0 cms 91.1%
90% nn ts1.0 normalized 100.0%
Matrix: bl, Window smoothing: 5, Matrix normalization: uniform
% cut gaps AUC
90% nn ts0.5 cms 72.3%
90% nn ts0.5 normalized 71.5%
90% nn ts1.0 cms 78.2%
90% nn ts1.0 normalized 100.0%
Matrix: lg, Window smoothing: 5, Matrix normalization: uniform
% cut gaps AUC
90% nn ts0.5 cms 93.6%
90% nn ts0.5 normalized 90.8%
90% nn ts1.0 cms 93.0%
90% nn ts1.0 normalized 100.0%
Matrix: bl, Window smoothing: 5, Matrix normalization: bgfreq
% cut gaps AUC
90% nn ts0.5 cms 92.7%
90% nn ts0.5 normalized 92.2%
90% nn ts1.0 cms 92.5%
90% nn ts1.0 normalized 100.0%
Matrix: lg, Window smoothing: 5, Matrix normalization: bgfreq
% cut gaps AUC
90% nn ts0.5 cms 95.8%
90% nn ts0.5 normalized 92.2%
90% nn ts1.0 cms 95.0%
90% nn ts1.0 normalized 100.0%
Matrix: bl, Window smoothing: 7, Matrix normalization: uniform
% cut gaps AUC
90% nn ts0.5 cms 67.6%
90% nn ts0.5 normalized 80.2%
90% nn ts1.0 cms 87.4%
90% nn ts1.0 normalized 100.0%
Matrix: lg, Window smoothing: 7, Matrix normalization: uniform
% cut gaps AUC
90% nn ts0.5 cms 90.8%
90% nn ts0.5 normalized 90.8%
90% nn ts1.0 cms 84.4%
90% nn ts1.0 normalized 100.0%
Matrix: bl, Window smoothing: 7, Matrix normalization: bgfreq
% cut gaps AUC
90% nn ts0.5 cms 70.1%
90% nn ts0.5 normalized 74.3%
90% nn ts1.0 cms 70.1%
90% nn ts1.0 normalized 100.0%
Matrix: lg, Window smoothing: 7, Matrix normalization: bgfreq
% cut gaps AUC
90% nn ts0.5 cms 85.2%
90% nn ts0.5 normalized 91.1%
90% nn ts1.0 cms 85.2%
90% nn ts1.0 normalized 98.9%
Matrix: bl, Window smoothing: 9, Matrix normalization: uniform
% cut gaps AUC
90% nn ts0.5 cms 84.9%
90% nn ts0.5 normalized 70.5%
90% nn ts1.0 cms 74.9%
90% nn ts1.0 normalized 100.0%
Matrix: lg, Window smoothing: 9, Matrix normalization: uniform
% cut gaps AUC
90% nn ts0.5 cms 95.8%
90% nn ts0.5 normalized 92.2%
90% nn ts1.0 cms 52.2%
90% nn ts1.0 normalized 100.0%
Matrix: bl, Window smoothing: 9, Matrix normalization: bgfreq
% cut gaps AUC
90% nn ts0.5 cms 73.7%
90% nn ts0.5 normalized 72.5%
90% nn ts1.0 cms 46.1%
90% nn ts1.0 normalized 100.0%
Matrix: lg, Window smoothing: 9, Matrix normalization: bgfreq
% cut gaps AUC
90% nn ts0.5 cms 95.3%
90% nn ts0.5 normalized 90.8%
90% nn ts1.0 cms 69.6%
90% nn ts1.0 normalized 100.0%
True positive rate
False positive rate
0.0
0.2
0.4
0.6
0.8
1.0

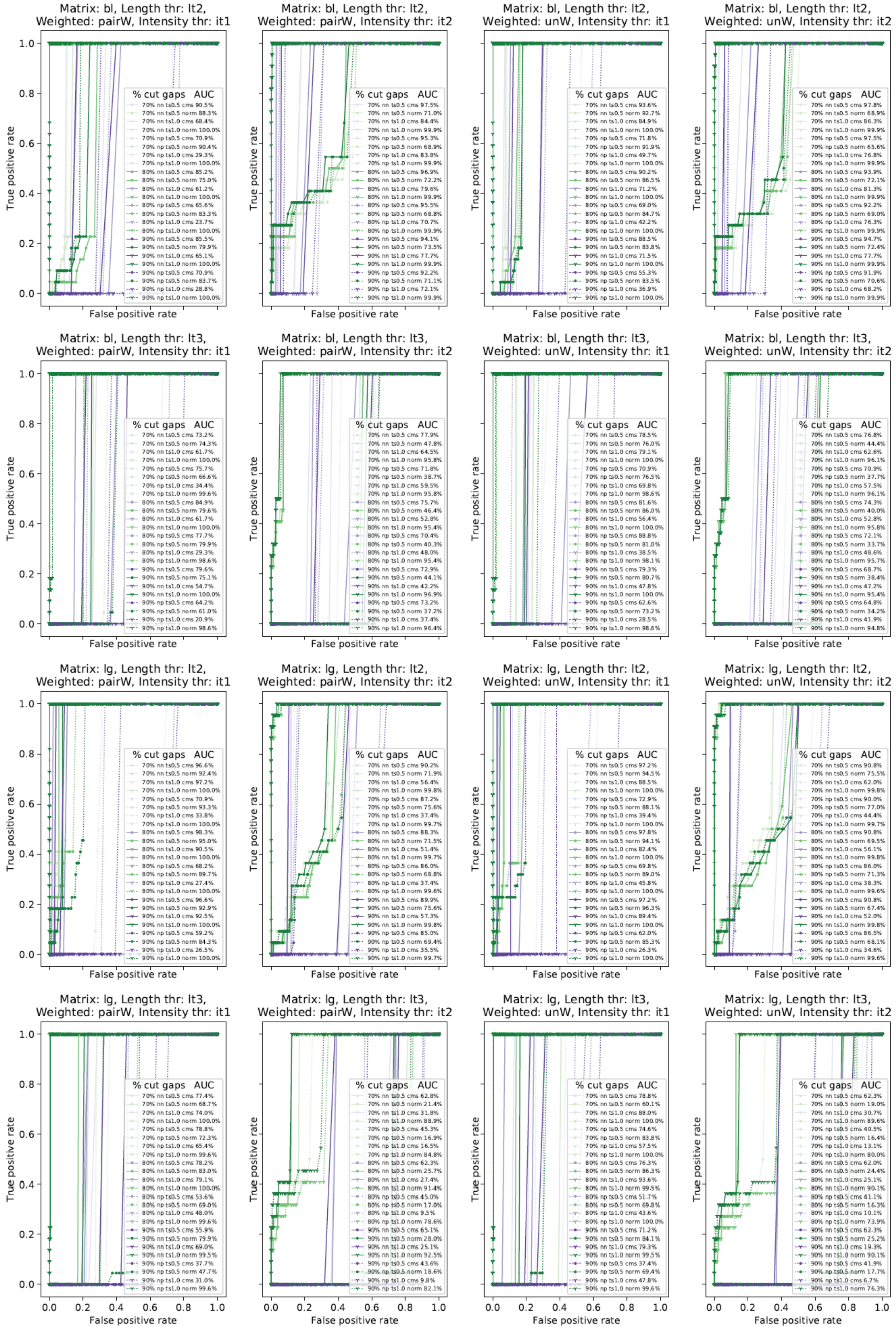
Matrix: bl, Length thr: lt2, Weighted: pairW, Intensity thr: it1
% cut gaps AUC
70% nn ts0.5 cms 90.5%
70% nn ts0.5 norm 88.3%
70% nn ts1.0 cms 68.4%
70% nn ts1.0 norm 100.0%
70% np ts0.5 cms 70.9%
70% np ts0.5 norm 90.4%
70% np ts1.0 cms 29.3%
70% np ts1.0 norm 100.0%
80% nn ts0.5 cms 85.2%
80% nn ts0.5 norm 75.0%
80% nn ts1.0 cms 61.2%
80% nn ts1.0 norm 100.0%
80% np ts0.5 cms 65.6%
80% np ts0.5 norm 83.3%
80% np ts1.0 cms 23.7%
80% np ts1.0 norm 100.0%
90% nn ts0.5 cms 85.5%
90% nn ts0.5 norm 79.9%
90% nn ts1.0 cms 65.1%
90% nn ts1.0 norm 100.0%
90% np ts0.5 cms 70.9%
90% np ts0.5 norm 83.7%
90% np ts1.0 cms 28.8%
90% np ts1.0 norm 100.0%
Matrix: bl, Length thr: lt2, Weighted: pairW, Intensity thr: it2
% cut gaps AUC
70% nn ts0.5 cms 97.5%
70% nn ts0.5 norm 71.0%
70% nn ts1.0 cms 84.4%
70% nn ts1.0 norm 99.9%
70% np ts0.5 cms 95.3%
70% np ts0.5 norm 68.9%
70% np ts1.0 cms 83.8%
70% np ts1.0 norm 99.9%
80% nn ts0.5 cms 96.9%
80% nn ts0.5 norm 72.2%
80% nn ts1.0 cms 79.6%
80% nn ts1.0 norm 99.9%
80% np ts0.5 cms 95.5%
80% np ts0.5 norm 68.8%
80% np ts1.0 cms 70.7%
80% np ts1.0 norm 99.9%
90% nn ts0.5 cms 94.1%
90% nn ts0.5 norm 73.5%
90% nn ts1.0 cms 77.7%
90% nn ts1.0 norm 99.9%
90% np ts0.5 cms 92.2%
90% np ts0.5 norm 71.1%
90% np ts1.0 cms 72.1%
90% np ts1.0 norm 99.9%
Matrix: bl, Length thr: lt2, Weighted: unW, Intensity thr: it1
% cut gaps AUC
70% nn ts0.5 cms 93.6%
70% nn ts0.5 norm 92.7%
70% nn ts1.0 cms 84.9%
70% nn ts1.0 norm 100.0%
70% np ts0.5 cms 71.8%
70% np ts0.5 norm 91.9%
70% np ts1.0 cms 49.7%
70% np ts1.0 norm 100.0%
80% nn ts0.5 cms 90.2%
80% nn ts0.5 norm 86.5%
80% nn ts1.0 cms 71.2%
80% nn ts1.0 norm 100.0%
80% np ts0.5 cms 69.0%
80% np ts0.5 norm 84.7%
80% np ts1.0 cms 42.2%
80% np ts1.0 norm 100.0%
90% nn ts0.5 cms 88.5%
90% nn ts0.5 norm 83.8%
90% nn ts1.0 cms 71.5%
90% nn ts1.0 norm 100.0%
90% np ts0.5 cms 55.3%
90% np ts0.5 norm 83.5%
90% np ts1.0 cms 36.9%
90% np ts1.0 norm 100.0%
Matrix: bl, Length thr: lt2, Weighted: unW, Intensity thr: it2
% cut gaps AUC
70% nn ts0.5 cms 97.8%
70% nn ts0.5 norm 68.9%
70% nn ts1.0 cms 86.3%
70% nn ts1.0 norm 99.9%
70% np ts0.5 cms 97.5%
70% np ts0.5 norm 65.6%
70% np ts1.0 cms 76.8%
70% np ts1.0 norm 99.9%
80% nn ts0.5 cms 93.9%
80% nn ts0.5 norm 72.1%
80% nn ts1.0 cms 81.3%
80% nn ts1.0 norm 99.9%
80% np ts0.5 cms 92.2%
80% np ts0.5 norm 69.0%
80% np ts1.0 cms 76.3%
80% np ts1.0 norm 99.9%
90% nn ts0.5 cms 94.7%
90% nn ts0.5 norm 72.4%
90% nn ts1.0 cms 77.7%
90% nn ts1.0 norm 99.9%
90% np ts0.5 cms 91.9%
90% np ts0.5 norm 70.6%
90% np ts1.0 cms 68.2%
90% np ts1.0 norm 99.9%
Matrix: bl, Length thr: lt3, Weighted: pairW, Intensity thr: it1
% cut gaps AUC
70% nn ts0.5 cms 73.2%
70% nn ts0.5 norm 74.3%
70% nn ts1.0 cms 61.7%
70% nn ts1.0 norm 100.0%
70% np ts0.5 cms 75.7%
70% np ts0.5 norm 66.6%
70% np ts1.0 cms 34.4%
70% np ts1.0 norm 99.6%
80% nn ts0.5 cms 84.9%
80% nn ts0.5 norm 79.6%
80% nn ts1.0 cms 61.7%
80% nn ts1.0 norm 100.0%
80% np ts0.5 cms 77.7%
80% np ts0.5 norm 79.9%
80% np ts1.0 cms 29.3%
80% np ts1.0 norm 98.6%
90% nn ts0.5 cms 79.6%
90% nn ts0.5 norm 75.1%
90% nn ts1.0 cms 54.7%
90% nn ts1.0 norm 100.0%
90% np ts0.5 cms 64.2%
90% np ts0.5 norm 61.0%
90% np ts1.0 cms 20.9%
90% np ts1.0 norm 98.6%
Matrix: bl, Length thr: lt3, Weighted: pairW, Intensity thr: it2
% cut gaps AUC
70% nn ts0.5 cms 77.9%
70% nn ts0.5 norm 47.8%
70% nn ts1.0 cms 64.5%
70% nn ts1.0 norm 95.8%
70% np ts0.5 cms 71.8%
70% np ts0.5 norm 38.7%
70% np ts1.0 cms 59.5%
70% np ts1.0 norm 95.8%
80% nn ts0.5 cms 75.7%
80% nn ts0.5 norm 46.4%
80% nn ts1.0 cms 52.8%
80% nn ts1.0 norm 95.4%
80% np ts0.5 cms 70.4%
80% np ts0.5 norm 40.3%
80% np ts1.0 cms 48.0%
80% np ts1.0 norm 95.4%
90% nn ts0.5 cms 72.9%
90% nn ts0.5 norm 44.1%
90% nn ts1.0 cms 42.2%
90% nn ts1.0 norm 96.9%
90% np ts0.5 cms 73.2%
90% np ts0.5 norm 37.2%
90% np ts1.0 cms 37.4%
90% np ts1.0 norm 96.4%
Matrix: bl, Length thr: lt3, Weighted: unW, Intensity thr: it1
% cut gaps AUC
70% nn ts0.5 cms 78.5%
70% nn ts0.5 norm 76.0%
70% nn ts1.0 cms 79.1%
70% nn ts1.0 norm 100.0%
70% np ts0.5 cms 70.9%
70% np ts0.5 norm 76.5%
70% np ts1.0 cms 69.8%
70% np ts1.0 norm 98.6%
80% nn ts0.5 cms 81.6%
80% nn ts0.5 norm 86.0%
80% nn ts1.0 cms 56.4%
80% nn ts1.0 norm 100.0%
80% np ts0.5 cms 88.8%
80% np ts0.5 norm 81.0%
80% np ts1.0 cms 38.5%
80% np ts1.0 norm 98.1%
90% nn ts0.5 cms 79.3%
90% nn ts0.5 norm 80.7%
90% nn ts1.0 cms 47.8%
90% nn ts1.0 norm 100.0%
90% np ts0.5 cms 62.6%
90% np ts0.5 norm 73.2%
90% np ts1.0 cms 28.5%
90% np ts1.0 norm 98.6%
Matrix: bl, Length thr: lt3, Weighted: unW, Intensity thr: it2
% cut gaps AUC
70% nn ts0.5 cms 76.8%
70% nn ts0.5 norm 44.4%
70% nn ts1.0 cms 62.6%
70% nn ts1.0 norm 96.1%
70% np ts0.5 cms 70.9%
70% np ts0.5 norm 37.7%
70% np ts1.0 cms 57.5%
70% np ts1.0 norm 96.1%
80% nn ts0.5 cms 74.3%
80% nn ts0.5 norm 40.0%
80% nn ts1.0 cms 52.8%
80% nn ts1.0 norm 95.8%
80% np ts0.5 cms 72.1%
80% np ts0.5 norm 33.7%
80% np ts1.0 cms 48.6%
80% np ts1.0 norm 95.7%
90% nn ts0.5 cms 68.7%
90% nn ts0.5 norm 38.4%
90% nn ts1.0 cms 47.2%
90% nn ts1.0 norm 95.4%
90% np ts0.5 cms 64.8%
90% np ts0.5 norm 34.2%
90% np ts1.0 cms 41.9%
90% np ts1.0 norm 94.8%
Matrix: lg, Length thr: lt2, Weighted: pairW, Intensity thr: it1
% cut gaps AUC
70% nn ts0.5 cms 96.6%
70% nn ts0.5 norm 92.4%
70% nn ts1.0 cms 97.2%
70% nn ts1.0 norm 100.0%
70% np ts0.5 cms 70.9%
70% np ts0.5 norm 93.3%
70% np ts1.0 cms 33.8%
70% np ts1.0 norm 100.0%
80% nn ts0.5 cms 98.3%
80% nn ts0.5 norm 95.0%
80% nn ts1.0 cms 90.5%
80% nn ts1.0 norm 100.0%
80% np ts0.5 cms 68.2%
80% np ts0.5 norm 89.7%
80% np ts1.0 cms 27.4%
80% np ts1.0 norm 100.0%
90% nn ts0.5 cms 96.6%
90% nn ts0.5 norm 92.9%
90% nn ts1.0 cms 92.5%
90% nn ts1.0 norm 100.0%
90% np ts0.5 cms 59.2%
90% np ts0.5 norm 84.3%
90% np ts1.0 cms 26.5%
90% np ts1.0 norm 100.0%
Matrix: lg, Length thr: lt2, Weighted: pairW, Intensity thr: it2
% cut gaps AUC
70% nn ts0.5 cms 90.2%
70% nn ts0.5 norm 71.9%
70% nn ts1.0 cms 56.4%
70% nn ts1.0 norm 99.8%
70% np ts0.5 cms 87.2%
70% np ts0.5 norm 75.6%
70% np ts1.0 cms 37.4%
70% np ts1.0 norm 99.7%
80% nn ts0.5 cms 88.3%
80% nn ts0.5 norm 71.5%
80% nn ts1.0 cms 51.4%
80% nn ts1.0 norm 99.7%
80% np ts0.5 cms 86.0%
80% np ts0.5 norm 68.8%
80% np ts1.0 cms 37.4%
80% np ts1.0 norm 99.6%
90% nn ts0.5 cms 89.9%
90% nn ts0.5 norm 75.6%
90% nn ts1.0 cms 57.3%
90% nn ts1.0 norm 99.8%
90% np ts0.5 cms 85.0%
90% np ts0.5 norm 69.4%
90% np ts1.0 cms 35.5%
90% np ts1.0 norm 99.7%
Matrix: lg, Length thr: lt2, Weighted: unW, Intensity thr: it1
% cut gaps AUC
70% nn ts0.5 cms 97.2%
70% nn ts0.5 norm 94.5%
70% nn ts1.0 cms 88.5%
70% nn ts1.0 norm 100.0%
70% np ts0.5 cms 72.9%
70% np ts0.5 norm 88.1%
70% np ts1.0 cms 39.4%
70% np ts1.0 norm 100.0%
80% nn ts0.5 cms 97.8%
80% nn ts0.5 norm 94.1%
80% nn ts1.0 cms 82.4%
80% nn ts1.0 norm 100.0%
80% np ts0.5 cms 69.8%
80% np ts0.5 norm 89.0%
80% np ts1.0 cms 45.8%
80% np ts1.0 norm 100.0%
90% nn ts0.5 cms 97.2%
90% nn ts0.5 norm 96.3%
90% nn ts1.0 cms 89.4%
90% nn ts1.0 norm 100.0%
90% np ts0.5 cms 62.0%
90% np ts0.5 norm 85.3%
90% np ts1.0 cms 26.3%
90% np ts1.0 norm 100.0%
Matrix: lg, Length thr: lt2, Weighted: unW, Intensity thr: it2
% cut gaps AUC
70% nn ts0.5 cms 90.8%
70% nn ts0.5 norm 75.5%
70% nn ts1.0 cms 62.0%
70% nn ts1.0 norm 99.8%
70% np ts0.5 cms 90.0%
70% np ts0.5 norm 77.0%
70% np ts1.0 cms 44.4%
70% np ts1.0 norm 99.7%
80% nn ts0.5 cms 90.8%
80% nn ts0.5 norm 69.5%
80% nn ts1.0 cms 56.1%
80% nn ts1.0 norm 99.8%
80% np ts0.5 cms 86.0%
80% np ts0.5 norm 71.3%
80% np ts1.0 cms 38.3%
80% np ts1.0 norm 99.6%
90% nn ts0.5 cms 90.8%
90% nn ts0.5 norm 67.4%
90% nn ts1.0 cms 52.0%
90% nn ts1.0 norm 99.8%
90% np ts0.5 cms 86.5%
90% np ts0.5 norm 68.1%
90% np ts1.0 cms 34.6%
90% np ts1.0 norm 99.6%
Matrix: lg, Length thr: lt3, Weighted: pairW, Intensity thr: it1
% cut gaps AUC
70% nn ts0.5 cms 77.4%
70% nn ts0.5 norm 68.7%
70% nn ts1.0 cms 74.0%
70% nn ts1.0 norm 100.0%
70% np ts0.5 cms 78.8%
70% np ts0.5 norm 72.3%
70% np ts1.0 cms 65.4%
70% np ts1.0 norm 99.6%
80% nn ts0.5 cms 78.2%
80% nn ts0.5 norm 83.0%
80% nn ts1.0 cms 79.1%
80% nn ts1.0 norm 100.0%
80% np ts0.5 cms 53.6%
80% np ts0.5 norm 69.0%
80% np ts1.0 cms 48.0%
80% np ts1.0 norm 99.6%
90% nn ts0.5 cms 55.9%
90% nn ts0.5 norm 79.9%
90% nn ts1.0 cms 69.0%
90% nn ts1.0 norm 99.5%
90% np ts0.5 cms 37.7%
90% np ts0.5 norm 47.7%
90% np ts1.0 cms 31.0%
90% np ts1.0 norm 99.6%
Matrix: lg, Length thr: lt3, Weighted: pairW, Intensity thr: it2
% cut gaps AUC
70% nn ts0.5 cms 62.8%
70% nn ts0.5 norm 21.4%
70% nn ts1.0 cms 31.8%
70% nn ts1.0 norm 88.9%
70% np ts0.5 cms 45.3%
70% np ts0.5 norm 16.9%
70% np ts1.0 cms 16.5%
70% np ts1.0 norm 84.8%
80% nn ts0.5 cms 62.3%
80% nn ts0.5 norm 25.7%
80% nn ts1.0 cms 27.4%
80% nn ts1.0 norm 91.4%
80% np ts0.5 cms 45.0%
80% np ts0.5 norm 17.0%
80% np ts1.0 cms 9.5%
80% np ts1.0 norm 78.6%
90% nn ts0.5 cms 65.1%
90% nn ts0.5 norm 28.0%
90% nn ts1.0 cms 25.1%
90% nn ts1.0 norm 92.5%
90% np ts0.5 cms 43.6%
90% np ts0.5 norm 18.6%
90% np ts1.0 cms 9.8%
90% np ts1.0 norm 82.1%
Matrix: lg, Length thr: lt3, Weighted: unW, Intensity thr: it1
% cut gaps AUC
70% nn ts0.5 cms 78.8%
70% nn ts0.5 norm 60.1%
70% nn ts1.0 cms 88.0%
70% nn ts1.0 norm 100.0%
70% np ts0.5 cms 74.6%
70% np ts0.5 norm 83.8%
70% np ts1.0 cms 57.5%
70% np ts1.0 norm 100.0%
80% nn ts0.5 cms 76.3%
80% nn ts0.5 norm 86.3%
80% nn ts1.0 cms 93.6%
80% nn ts1.0 norm 99.5%
80% np ts0.5 cms 51.7%
80% np ts0.5 norm 69.8%
80% np ts1.0 cms 43.6%
80% np ts1.0 norm 100.0%
90% nn ts0.5 cms 71.2%
90% nn ts0.5 norm 84.1%
90% nn ts1.0 cms 79.3%
90% nn ts1.0 norm 99.5%
90% np ts0.5 cms 37.4%
90% np ts0.5 norm 69.4%
90% np ts1.0 cms 47.8%
90% np ts1.0 norm 99.6%
Matrix: lg, Length thr: lt3, Weighted: unW, Intensity thr: it2
% cut gaps AUC
70% nn ts0.5 cms 62.3%
70% nn ts0.5 norm 19.0%
70% nn ts1.0 cms 30.7%
70% nn ts1.0 norm 89.6%
70% np ts0.5 cms 40.5%
70% np ts0.5 norm 16.4%
70% np ts1.0 cms 13.1%
70% np ts1.0 norm 80.0%
80% nn ts0.5 cms 62.0%
80% nn ts0.5 norm 24.4%
80% nn ts1.0 cms 25.1%
80% nn ts1.0 norm 90.1%
80% np ts0.5 cms 41.1%
80% np ts0.5 norm 16.3%
80% np ts1.0 cms 10.1%
80% np ts1.0 norm 73.9%
90% nn ts0.5 cms 62.3%
90% nn ts0.5 norm 25.2%
90% nn ts1.0 cms 19.3%
90% nn ts1.0 norm 90.1%
90% np ts0.5 cms 41.9%
90% np ts0.5 norm 17.7%
90% np ts1.0 cms 6.7%
90% np ts1.0 norm 76.3%
True positive rate
False positive rate
0.0
0.2
0.4
0.6
0.8
1.0

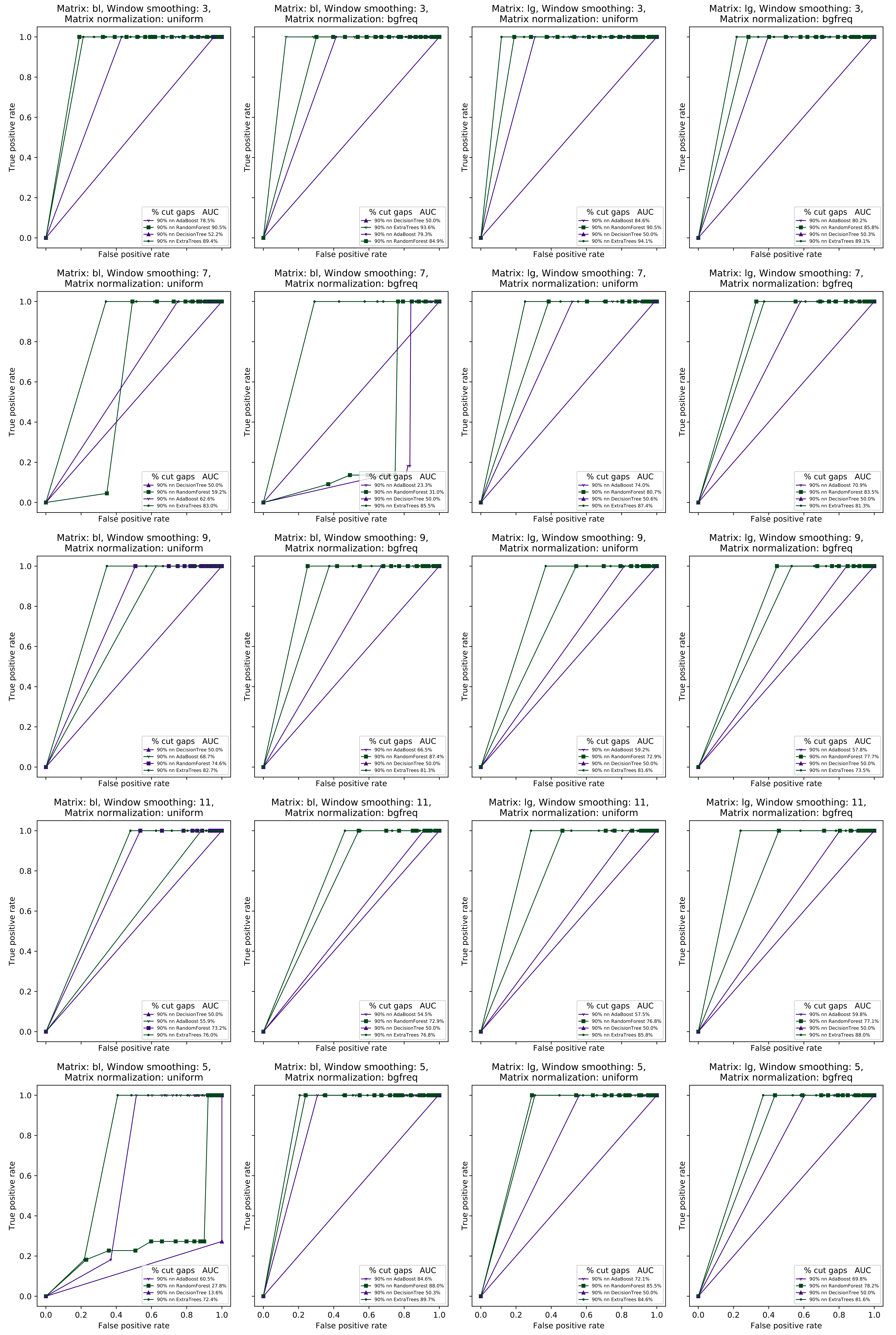
Matrix: bl, Window smoothing: 3, Matrix normalization: uniform
% cut gaps AUC
90% nn AdaBoost 78.5%
90% nn RandomForest 90.5%
90% nn DecisionTree 52.2%
90% nn ExtraTrees 89.4%
Matrix: bl, Window smoothing: 3, Matrix normalization: bgfreq
% cut gaps AUC
90% nn DecisionTree 50.0%
90% nn ExtraTrees 93.6%
90% nn AdaBoost 79.3%
90% nn RandomForest 84.9%
Matrix: lg, Window smoothing: 3, Matrix normalization: uniform
% cut gaps AUC
90% nn AdaBoost 84.6%
90% nn RandomForest 90.5%
90% nn DecisionTree 50.0%
90% nn ExtraTrees 94.1%
Matrix: lg, Window smoothing: 3, Matrix normalization: bgfreq
% cut gaps AUC
90% nn AdaBoost 80.2%
90% nn RandomForest 85.8%
90% nn DecisionTree 50.3%
90% nn ExtraTrees 89.1%
Matrix: bl, Window smoothing: 7, Matrix normalization: uniform
% cut gaps AUC
90% nn DecisionTree 50.0%
90% nn RandomForest 59.2%
90% nn AdaBoost 62.6%
90% nn ExtraTrees 83.0%
Matrix: bl, Window smoothing: 7, Matrix normalization: bgfreq
% cut gaps AUC
90% nn AdaBoost 23.3%
90% nn RandomForest 31.0%
90% nn DecisionTree 50.0%
90% nn ExtraTrees 85.5%
Matrix: lg, Window smoothing: 7, Matrix normalization: uniform
% cut gaps AUC
90% nn AdaBoost 74.0%
90% nn RandomForest 80.7%
90% nn DecisionTree 50.6%
90% nn ExtraTrees 87.4%
Matrix: lg, Window smoothing: 7, Matrix normalization: bgfreq
% cut gaps AUC
90% nn AdaBoost 70.9%
90% nn RandomForest 83.5%
90% nn DecisionTree 50.0%
90% nn ExtraTrees 81.3%
Matrix: bl, Window smoothing: 9, Matrix normalization: uniform
% cut gaps AUC
90% nn DecisionTree 50.0%
90% nn AdaBoost 68.7%
90% nn RandomForest 74.6%
90% nn ExtraTrees 82.7%
Matrix: bl, Window smoothing: 9, Matrix normalization: bgfreq
% cut gaps AUC
90% nn AdaBoost 66.5%
90% nn RandomForest 87.4%
90% nn DecisionTree 50.0%
90% nn ExtraTrees 81.3%
Matrix: lg, Window smoothing: 9, Matrix normalization: uniform
% cut gaps AUC
90% nn AdaBoost 59.2%
90% nn RandomForest 72.9%
90% nn DecisionTree 50.0%
90% nn ExtraTrees 81.6%
Matrix: lg, Window smoothing: 9, Matrix normalization: bgfreq
% cut gaps AUC
90% nn AdaBoost 57.8%
90% nn RandomForest 77.7%
90% nn DecisionTree 50.0%
90% nn ExtraTrees 73.5%
Matrix: bl, Window smoothing: 11, Matrix normalization: uniform
% cut gaps AUC
90% nn DecisionTree 50.0%
90% nn AdaBoost 55.9%
90% nn RandomForest 73.2%
90% nn ExtraTrees 76.0%
Matrix: bl, Window smoothing: 11, Matrix normalization: bgfreq
% cut gaps AUC
90% nn AdaBoost 54.5%
90% nn RandomForest 72.9%
90% nn DecisionTree 50.0%
90% nn ExtraTrees 76.8%
Matrix: lg, Window smoothing: 11, Matrix normalization: uniform
% cut gaps AUC
90% nn AdaBoost 57.5%
90% nn RandomForest 76.8%
90% nn DecisionTree 50.0%
90% nn ExtraTrees 85.8%
Matrix: lg, Window smoothing: 11, Matrix normalization: bgfreq
% cut gaps AUC
90% nn AdaBoost 59.8%
90% nn RandomForest 77.1%
90% nn DecisionTree 50.0%
90% nn ExtraTrees 88.0%
Matrix: bl, Window smoothing: 5, Matrix normalization: uniform
% cut gaps AUC
90% nn AdaBoost 60.5%
90% nn RandomForest 27.8%
90% nn DecisionTree 13.6%
90% nn ExtraTrees 72.4%
Matrix: bl, Window smoothing: 5, Matrix normalization: bgfreq
% cut gaps AUC
90% nn AdaBoost 84.6%
90% nn RandomForest 88.0%
90% nn DecisionTree 50.3%
90% nn ExtraTrees 89.7%
Matrix: lg, Window smoothing: 5, Matrix normalization: uniform
% cut gaps AUC
90% nn AdaBoost 72.1%
90% nn RandomForest 85.5%
90% nn DecisionTree 50.0%
90% nn ExtraTrees 84.6%
Matrix: lg, Window smoothing: 5, Matrix normalization: bgfreq
% cut gaps AUC
90% nn AdaBoost 69.8%
90% nn RandomForest 78.2%
90% nn DecisionTree 50.0%
90% nn ExtraTrees 81.6%
True positive rate
False positive rate

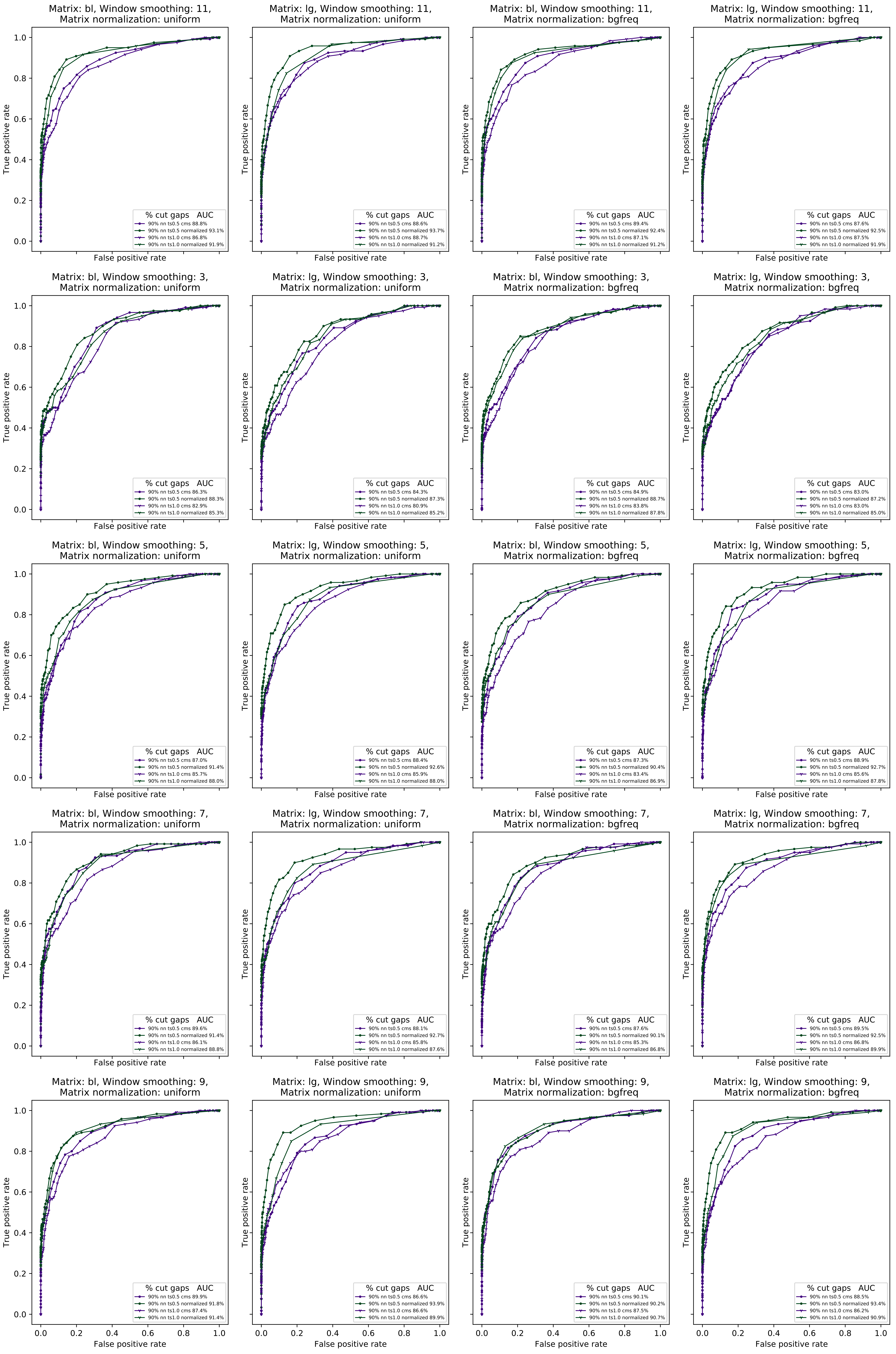

Matrix: bl, Window smoothing: 11, Matrix normalization: uniform
% cut gaps AUC
90% nn ts0.5 cms 88.8%
90% nn ts0.5 normalized 93.1%
90% nn ts1.0 cms 86.8%
90% nn ts1.0 normalized 91.9%
Matrix: lg, Window smoothing: 11, Matrix normalization: uniform
% cut gaps AUC
90% nn ts0.5 cms 88.6%
90% nn ts0.5 normalized 93.7%
90% nn ts1.0 cms 88.7%
90% nn ts1.0 normalized 91.2%
Matrix: bl, Window smoothing: 11, Matrix normalization: bgfreq
% cut gaps AUC
90% nn ts0.5 cms 89.4%
90% nn ts0.5 normalized 92.4%
90% nn ts1.0 cms 87.1%
90% nn ts1.0 normalized 91.2%
Matrix: lg, Window smoothing: 11, Matrix normalization: bgfreq
% cut gaps AUC
90% nn ts0.5 cms 87.6%
90% nn ts0.5 normalized 92.5%
90% nn ts1.0 cms 87.5%
90% nn ts1.0 normalized 91.9%
Matrix: bl, Window smoothing: 3, Matrix normalization: uniform
% cut gaps AUC
90% nn ts0.5 cms 86.3%
90% nn ts0.5 normalized 88.3%
90% nn ts1.0 cms 82.9%
90% nn ts1.0 normalized 85.3%
Matrix: lg, Window smoothing: 3, Matrix normalization: uniform
% cut gaps AUC
90% nn ts0.5 cms 84.3%
90% nn ts0.5 normalized 87.3%
90% nn ts1.0 cms 80.9%
90% nn ts1.0 normalized 85.2%
Matrix: bl, Window smoothing: 3, Matrix normalization: bgfreq
% cut gaps AUC
90% nn ts0.5 cms 84.9%
90% nn ts0.5 normalized 88.7%
90% nn ts1.0 cms 83.8%
90% nn ts1.0 normalized 87.8%
Matrix: lg, Window smoothing: 3, Matrix normalization: bgfreq
% cut gaps AUC
90% nn ts0.5 cms 83.0%
90% nn ts0.5 normalized 87.2%
90% nn ts1.0 cms 83.0%
90% nn ts1.0 normalized 85.0%
Matrix: bl, Window smoothing: 5, Matrix normalization: uniform
% cut gaps AUC
90% nn ts0.5 cms 87.0%
90% nn ts0.5 normalized 91.4%
90% nn ts1.0 cms 85.7%
90% nn ts1.0 normalized 88.0%
Matrix: lg, Window smoothing: 5, Matrix normalization: uniform
% cut gaps AUC
90% nn ts0.5 cms 88.4%
90% nn ts0.5 normalized 92.6%
90% nn ts1.0 cms 85.9%
90% nn ts1.0 normalized 88.0%
Matrix: bl, Window smoothing: 5, Matrix normalization: bgfreq
% cut gaps AUC
90% nn ts0.5 cms 87.3%
90% nn ts0.5 normalized 90.4%
90% nn ts1.0 cms 83.4%
90% nn ts1.0 normalized 86.9%
Matrix: lg, Window smoothing: 5, Matrix normalization: bgfreq
% cut gaps AUC
90% nn ts0.5 cms 88.9%
90% nn ts0.5 normalized 92.7%
90% nn ts1.0 cms 85.6%
90% nn ts1.0 normalized 87.8%
Matrix: bl, Window smoothing: 7, Matrix normalization: uniform
% cut gaps AUC
90% nn ts0.5 cms 89.6%
90% nn ts0.5 normalized 91.4%
90% nn ts1.0 cms 86.1%
90% nn ts1.0 normalized 88.8%
Matrix: lg, Window smoothing: 7, Matrix normalization: uniform
% cut gaps AUC
90% nn ts0.5 cms 88.1%
90% nn ts0.5 normalized 92.7%
90% nn ts1.0 cms 85.8%
90% nn ts1.0 normalized 87.6%
Matrix: bl, Window smoothing: 7, Matrix normalization: bgfreq
% cut gaps AUC
90% nn ts0.5 cms 87.6%
90% nn ts0.5 normalized 90.1%
90% nn ts1.0 cms 85.3%
90% nn ts1.0 normalized 86.8%
Matrix: lg, Window smoothing: 7, Matrix normalization: bgfreq
% cut gaps AUC
90% nn ts0.5 cms 89.5%
90% nn ts0.5 normalized 92.5%
90% nn ts1.0 cms 86.8%
90% nn ts1.0 normalized 89.9%
Matrix: bl, Window smoothing: 9, Matrix normalization: uniform
% cut gaps AUC
90% nn ts0.5 cms 89.9%
90% nn ts0.5 normalized 91.8%
90% nn ts1.0 cms 87.4%
90% nn ts1.0 normalized 91.4%
Matrix: lg, Window smoothing: 9, Matrix normalization: uniform
% cut gaps AUC
90% nn ts0.5 cms 86.6%
90% nn ts0.5 normalized 93.9%
90% nn ts1.0 cms 86.6%
90% nn ts1.0 normalized 89.9%
Matrix: bl, Window smoothing: 9, Matrix normalization: bgfreq
% cut gaps AUC
90% nn ts0.5 cms 90.1%
90% nn ts0.5 normalized 90.2%
90% nn ts1.0 cms 87.5%
90% nn ts1.0 normalized 90.7%
Matrix: lg, Window smoothing: 9, Matrix normalization: bgfreq
% cut gaps AUC
90% nn ts0.5 cms 88.5%
90% nn ts0.5 normalized 93.4%
90% nn ts1.0 cms 86.2%
90% nn ts1.0 normalized 90.9%
True positive rate
False positive rate

BBS vs PRST

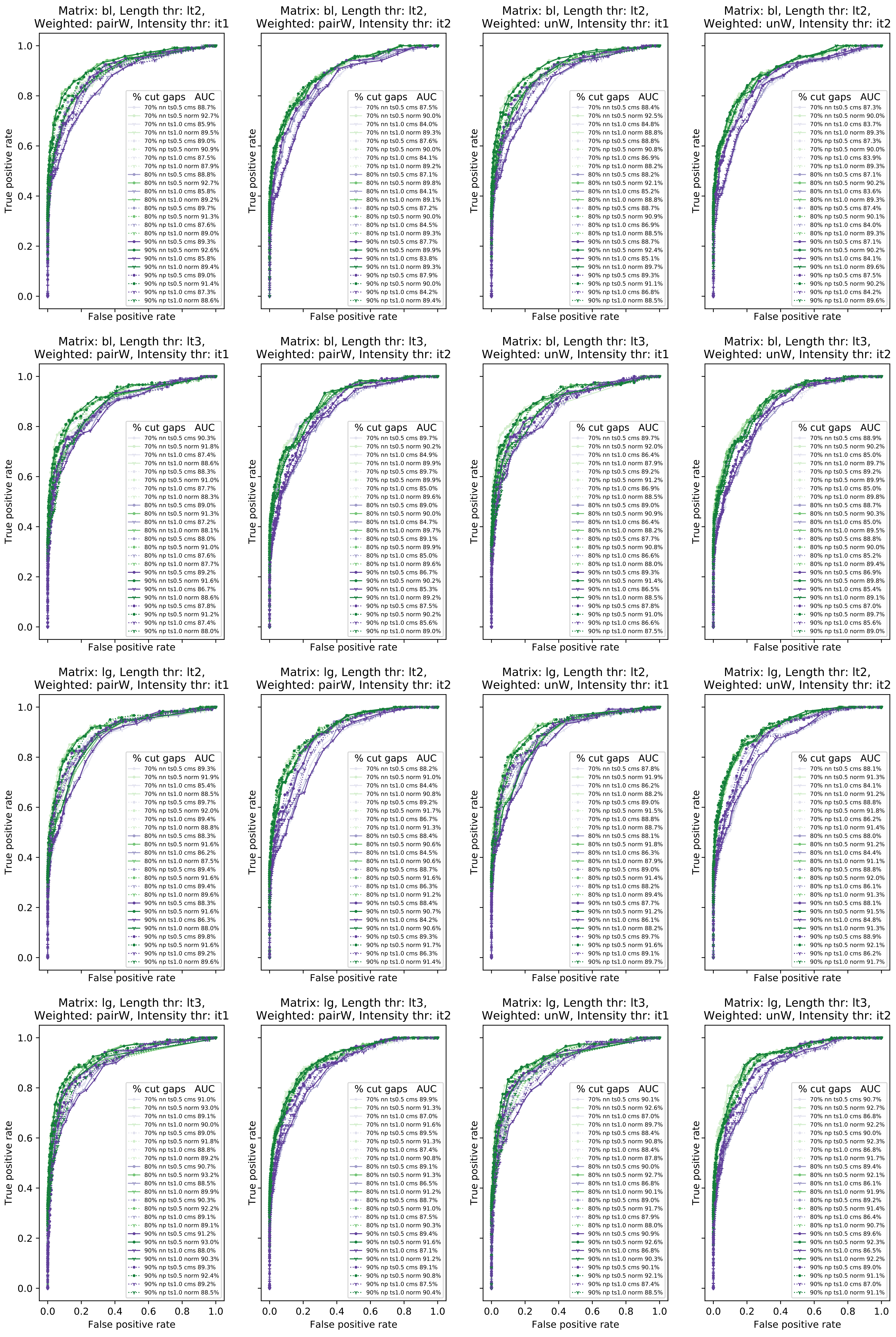
Matrix: bl, Length thr: lt2, Weighted: pairW, Intensity thr: it1
Matrix: bl, Length thr: lt2, Weighted: pairW, Intensity thr: it2
Matrix: bl, Length thr: lt2, Weighted: unW, Intensity thr: it1
Matrix: bl, Length thr: lt2, Weighted: unW, Intensity thr: it2
Matrix: bl, Length thr: lt3, Weighted: pairW, Intensity thr: it1
Matrix: bl, Length thr: lt3, Weighted: pairW, Intensity thr: it2
Matrix: bl, Length thr: lt3, Weighted: unW, Intensity thr: it1
Matrix: bl, Length thr: lt3, Weighted: unW, Intensity thr: it2
Matrix: lg, Length thr: lt2, Weighted: pairW, Intensity thr: it1
Matrix: lg, Length thr: lt2, Weighted: pairW, Intensity thr: it2
Matrix: lg, Length thr: lt2, Weighted: unW, Intensity thr: it1
Matrix: lg, Length thr: lt2, Weighted: unW, Intensity thr: it2
Matrix: lg, Length thr: lt3, Weighted: pairW, Intensity thr: it1
Matrix: lg, Length thr: lt3, Weighted: pairW, Intensity thr: it2
Matrix: lg, Length thr: lt3, Weighted: unW, Intensity thr: it1
Matrix: lg, Length thr: lt3, Weighted: unW, Intensity thr: it2
% cut gaps AUC
True positive rate
False positive rate

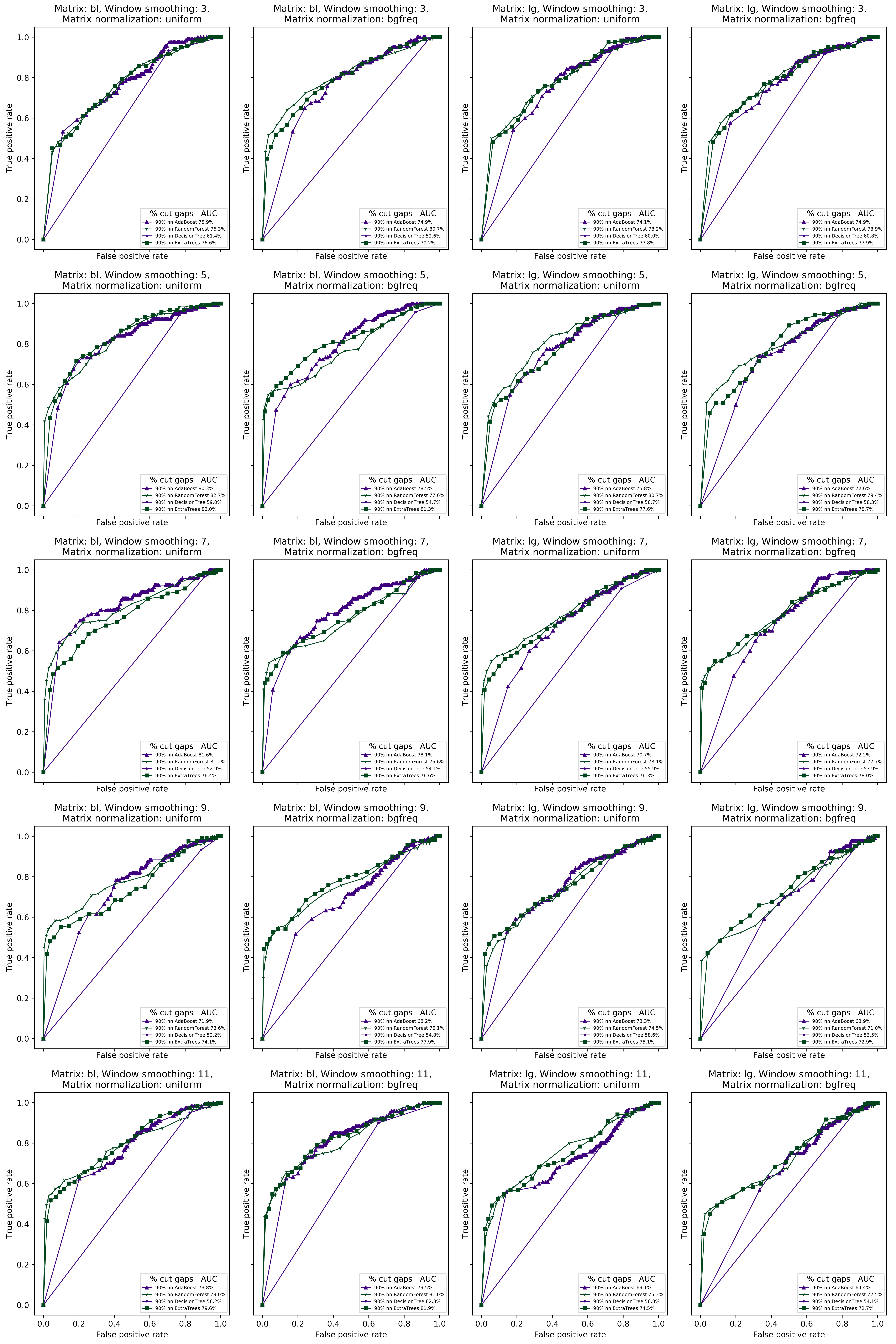

Matrix: bl, Window smoothing: 3, Matrix normalization: uniform
% cut gaps AUC
90% nn AdaBoost 75.9%
90% nn RandomForest 76.3%
90% nn DecisionTree 61.4%
90% nn ExtraTrees 76.6%
Matrix: bl, Window smoothing: 3, Matrix normalization: bgfreq
% cut gaps AUC
90% nn AdaBoost 74.9%
90% nn RandomForest 80.7%
90% nn DecisionTree 52.6%
90% nn ExtraTrees 79.2%
Matrix: lg, Window smoothing: 3, Matrix normalization: uniform
% cut gaps AUC
90% nn AdaBoost 74.1%
90% nn RandomForest 78.2%
90% nn DecisionTree 60.0%
90% nn ExtraTrees 77.8%
Matrix: lg, Window smoothing: 3, Matrix normalization: bgfreq
% cut gaps AUC
90% nn AdaBoost 74.9%
90% nn RandomForest 78.9%
90% nn DecisionTree 60.8%
90% nn ExtraTrees 77.9%
Matrix: bl, Window smoothing: 5, Matrix normalization: uniform
% cut gaps AUC
90% nn AdaBoost 80.3%
90% nn RandomForest 82.7%
90% nn DecisionTree 59.0%
90% nn ExtraTrees 83.0%
Matrix: bl, Window smoothing: 5, Matrix normalization: bgfreq
% cut gaps AUC
90% nn AdaBoost 78.5%
90% nn RandomForest 77.6%
90% nn DecisionTree 54.7%
90% nn ExtraTrees 81.3%
Matrix: lg, Window smoothing: 5, Matrix normalization: uniform
% cut gaps AUC
90% nn AdaBoost 75.8%
90% nn RandomForest 80.7%
90% nn DecisionTree 58.7%
90% nn ExtraTrees 77.6%
Matrix: lg, Window smoothing: 5, Matrix normalization: bgfreq
% cut gaps AUC
90% nn AdaBoost 72.6%
90% nn RandomForest 79.4%
90% nn DecisionTree 58.3%
90% nn ExtraTrees 78.7%
Matrix: bl, Window smoothing: 7, Matrix normalization: uniform
% cut gaps AUC
90% nn AdaBoost 81.6%
90% nn RandomForest 81.2%
90% nn DecisionTree 52.9%
90% nn ExtraTrees 76.4%
Matrix: bl, Window smoothing: 7, Matrix normalization: bgfreq
% cut gaps AUC
90% nn AdaBoost 78.1%
90% nn RandomForest 75.6%
90% nn DecisionTree 54.1%
90% nn ExtraTrees 76.6%
Matrix: lg, Window smoothing: 7, Matrix normalization: uniform
% cut gaps AUC
90% nn AdaBoost 70.7%
90% nn RandomForest 78.1%
90% nn DecisionTree 55.9%
90% nn ExtraTrees 76.3%
Matrix: lg, Window smoothing: 7, Matrix normalization: bgfreq
% cut gaps AUC
90% nn AdaBoost 72.2%
90% nn RandomForest 77.7%
90% nn DecisionTree 53.9%
90% nn ExtraTrees 78.0%
Matrix: bl, Window smoothing: 9, Matrix normalization: uniform
% cut gaps AUC
90% nn AdaBoost 71.9%
90% nn RandomForest 78.6%
90% nn DecisionTree 52.2%
90% nn ExtraTrees 74.1%
Matrix: bl, Window smoothing: 9, Matrix normalization: bgfreq
% cut gaps AUC
90% nn AdaBoost 68.2%
90% nn RandomForest 76.1%
90% nn DecisionTree 54.8%
90% nn ExtraTrees 77.9%
Matrix: lg, Window smoothing: 9, Matrix normalization: uniform
% cut gaps AUC
90% nn AdaBoost 73.3%
90% nn RandomForest 74.5%
90% nn DecisionTree 58.6%
90% nn ExtraTrees 75.1%
Matrix: lg, Window smoothing: 9, Matrix normalization: bgfreq
% cut gaps AUC
90% nn AdaBoost 63.9%
90% nn RandomForest 71.0%
90% nn DecisionTree 53.5%
90% nn ExtraTrees 72.9%
Matrix: bl, Window smoothing: 11, Matrix normalization: uniform
% cut gaps AUC
90% nn AdaBoost 73.8%
90% nn RandomForest 79.0%
90% nn DecisionTree 56.2%
90% nn ExtraTrees 79.6%
Matrix: bl, Window smoothing: 11, Matrix normalization: bgfreq
% cut gaps AUC
90% nn AdaBoost 79.5%
90% nn RandomForest 81.0%
90% nn DecisionTree 62.3%
90% nn ExtraTrees 81.9%
Matrix: lg, Window smoothing: 11, Matrix normalization: uniform
% cut gaps AUC
90% nn AdaBoost 69.1%
90% nn RandomForest 75.3%
90% nn DecisionTree 56.8%
90% nn ExtraTrees 74.5%
Matrix: lg, Window smoothing: 11, Matrix normalization: bgfreq
% cut gaps AUC
90% nn AdaBoost 64.4%
90% nn RandomForest 72.5%
90% nn DecisionTree 54.1%
90% nn ExtraTrees 72.7%
True positive rate
False positive rate

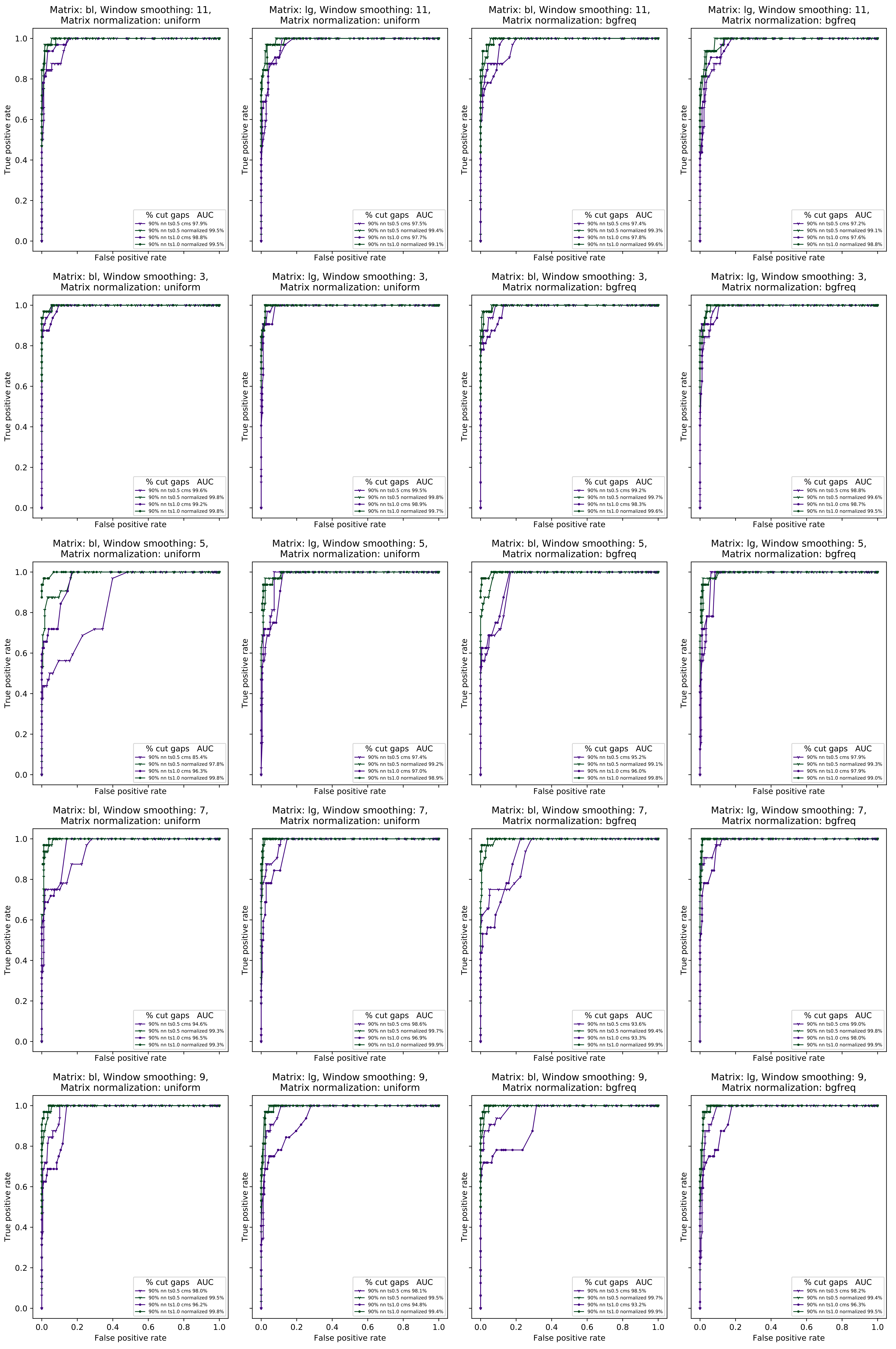
Matrix: bl, Window smoothing: 11, Matrix normalization: uniform
% cut gaps AUC
90% nn ts0.5 cms 97.9%
90% nn ts0.5 normalized 99.5%
90% nn ts1.0 cms 98.8%
90% nn ts1.0 normalized 99.5%
Matrix: lg, Window smoothing: 11, Matrix normalization: uniform
% cut gaps AUC
90% nn ts0.5 cms 97.5%
90% nn ts0.5 normalized 99.4%
90% nn ts1.0 cms 97.7%
90% nn ts1.0 normalized 99.1%
Matrix: bl, Window smoothing: 11, Matrix normalization: bgfreq
% cut gaps AUC
90% nn ts0.5 cms 97.4%
90% nn ts0.5 normalized 99.3%
90% nn ts1.0 cms 97.8%
90% nn ts1.0 normalized 99.6%
Matrix: lg, Window smoothing: 11, Matrix normalization: bgfreq
% cut gaps AUC
90% nn ts0.5 cms 97.2%
90% nn ts0.5 normalized 99.1%
90% nn ts1.0 cms 97.6%
90% nn ts1.0 normalized 98.8%
Matrix: bl, Window smoothing: 3, Matrix normalization: uniform
% cut gaps AUC
90% nn ts0.5 cms 99.6%
90% nn ts0.5 normalized 99.8%
90% nn ts1.0 cms 99.2%
90% nn ts1.0 normalized 99.8%
Matrix: lg, Window smoothing: 3, Matrix normalization: uniform
% cut gaps AUC
90% nn ts0.5 cms 99.5%
90% nn ts0.5 normalized 99.8%
90% nn ts1.0 cms 98.9%
90% nn ts1.0 normalized 99.7%
Matrix: bl, Window smoothing: 3, Matrix normalization: bgfreq
% cut gaps AUC
90% nn ts0.5 cms 99.2%
90% nn ts0.5 normalized 99.7%
90% nn ts1.0 cms 98.3%
90% nn ts1.0 normalized 99.6%
Matrix: lg, Window smoothing: 3, Matrix normalization: bgfreq
% cut gaps AUC
90% nn ts0.5 cms 98.8%
90% nn ts0.5 normalized 99.6%
90% nn ts1.0 cms 98.7%
90% nn ts1.0 normalized 99.5%
Matrix: bl, Window smoothing: 5, Matrix normalization: uniform
% cut gaps AUC
90% nn ts0.5 cms 85.4%
90% nn ts0.5 normalized 97.8%
90% nn ts1.0 cms 96.3%
90% nn ts1.0 normalized 99.8%
Matrix: lg, Window smoothing: 5, Matrix normalization: uniform
% cut gaps AUC
90% nn ts0.5 cms 97.4%
90% nn ts0.5 normalized 99.2%
90% nn ts1.0 cms 97.0%
90% nn ts1.0 normalized 98.9%
Matrix: bl, Window smoothing: 5, Matrix normalization: bgfreq
% cut gaps AUC
90% nn ts0.5 cms 95.2%
90% nn ts0.5 normalized 99.1%
90% nn ts1.0 cms 96.0%
90% nn ts1.0 normalized 99.8%
Matrix: lg, Window smoothing: 5, Matrix normalization: bgfreq
% cut gaps AUC
90% nn ts0.5 cms 97.9%
90% nn ts0.5 normalized 99.3%
90% nn ts1.0 cms 97.9%
90% nn ts1.0 normalized 99.0%
Matrix: bl, Window smoothing: 7, Matrix normalization: uniform
% cut gaps AUC
90% nn ts0.5 cms 94.6%
90% nn ts0.5 normalized 99.3%
90% nn ts1.0 cms 96.5%
90% nn ts1.0 normalized 99.3%
Matrix: lg, Window smoothing: 7, Matrix normalization: uniform
% cut gaps AUC
90% nn ts0.5 cms 98.6%
90% nn ts0.5 normalized 99.7%
90% nn ts1.0 cms 96.9%
90% nn ts1.0 normalized 99.9%
Matrix: bl, Window smoothing: 7, Matrix normalization: bgfreq
% cut gaps AUC
90% nn ts0.5 cms 93.6%
90% nn ts0.5 normalized 99.4%
90% nn ts1.0 cms 93.3%
90% nn ts1.0 normalized 99.9%
Matrix: lg, Window smoothing: 7, Matrix normalization: bgfreq
% cut gaps AUC
90% nn ts0.5 cms 99.0%
90% nn ts0.5 normalized 99.8%
90% nn ts1.0 cms 98.0%
90% nn ts1.0 normalized 99.9%
Matrix: bl, Window smoothing: 9, Matrix normalization: uniform
% cut gaps AUC
90% nn ts0.5 cms 98.0%
90% nn ts0.5 normalized 99.5%
90% nn ts1.0 cms 96.2%
90% nn ts1.0 normalized 99.8%
Matrix: lg, Window smoothing: 9, Matrix normalization: uniform
% cut gaps AUC
90% nn ts0.5 cms 98.1%
90% nn ts0.5 normalized 99.5%
90% nn ts1.0 cms 94.8%
90% nn ts1.0 normalized 99.4%
Matrix: bl, Window smoothing: 9, Matrix normalization: bgfreq
% cut gaps AUC
90% nn ts0.5 cms 98.5%
90% nn ts0.5 normalized 99.7%
90% nn ts1.0 cms 93.2%
90% nn ts1.0 normalized 99.9%
Matrix: lg, Window smoothing: 9, Matrix normalization: bgfreq
% cut gaps AUC
90% nn ts0.5 cms 98.2%
90% nn ts0.5 normalized 99.4%
90% nn ts1.0 cms 96.3%
90% nn ts1.0 normalized 99.5%
True positive rate
False positive rate
0.0
0.2
0.4
0.6
0.8
1.0

BBS vs rProt

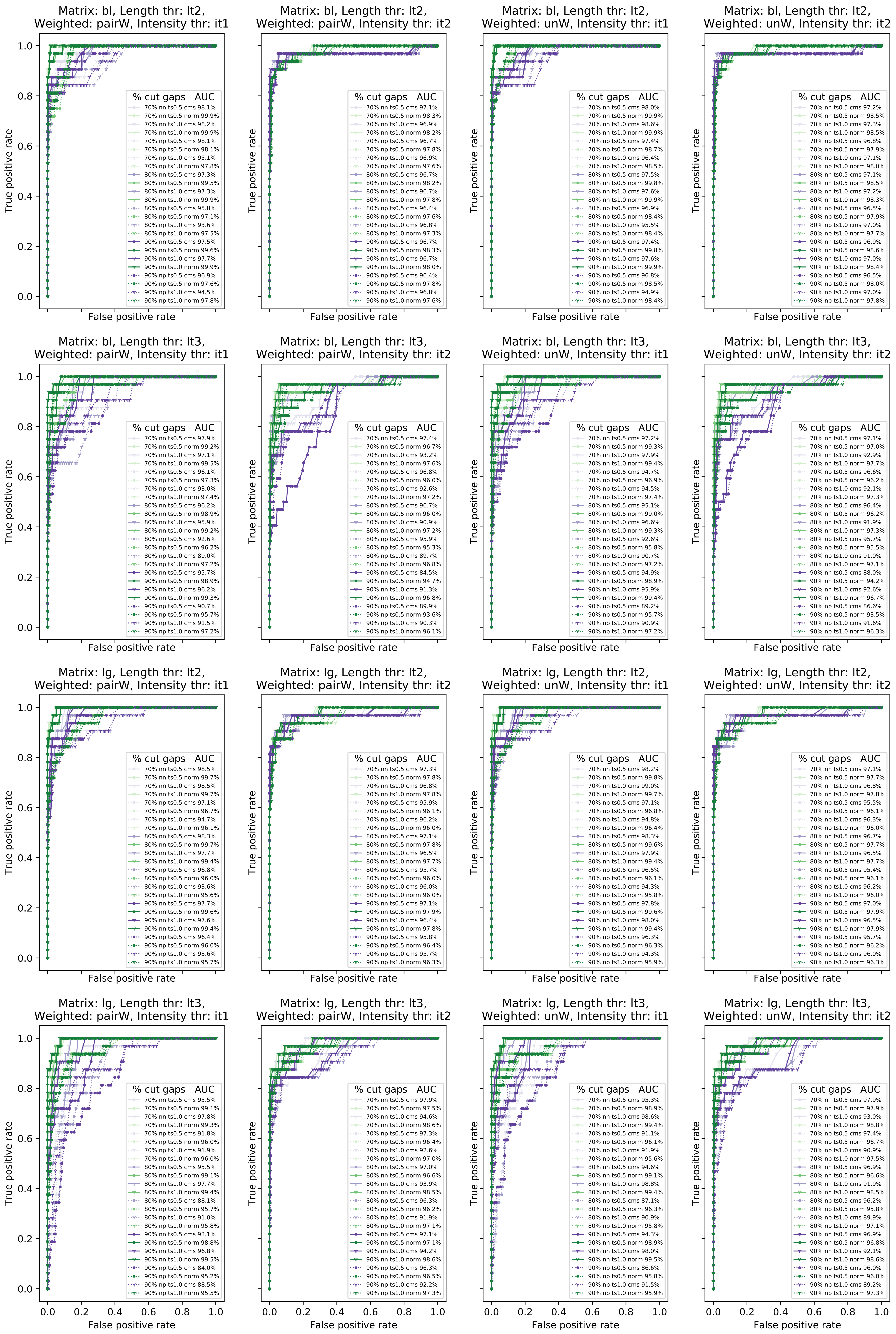

Matrix: bl, Length thr: lt2, Weighted: pairW, Intensity thr: it1
% cut gaps AUC
70% nn ts0.5 cms 98.1%
70% nn ts0.5 norm 99.9%
70% nn ts1.0 cms 98.2%
70% nn ts1.0 norm 99.9%
70% np ts0.5 cms 98.1%
70% np ts0.5 norm 98.1%
70% np ts1.0 cms 95.1%
70% np ts1.0 norm 97.8%
80% nn ts0.5 cms 97.3%
80% nn ts0.5 norm 99.5%
80% nn ts1.0 cms 97.3%
80% nn ts1.0 norm 99.9%
80% np ts0.5 cms 95.8%
80% np ts0.5 norm 97.1%
80% np ts1.0 cms 93.6%
80% np ts1.0 norm 97.5%
90% nn ts0.5 cms 97.5%
90% nn ts0.5 norm 99.6%
90% nn ts1.0 cms 97.7%
90% nn ts1.0 norm 99.9%
90% np ts0.5 cms 96.9%
90% np ts0.5 norm 97.6%
90% np ts1.0 cms 94.5%
90% np ts1.0 norm 97.8%
Matrix: bl, Length thr: lt2, Weighted: pairW, Intensity thr: it2
% cut gaps AUC
70% nn ts0.5 cms 97.1%
70% nn ts0.5 norm 98.3%
70% nn ts1.0 cms 96.9%
70% nn ts1.0 norm 98.2%
70% np ts0.5 cms 96.7%
70% np ts0.5 norm 97.8%
70% np ts1.0 cms 96.9%
70% np ts1.0 norm 97.6%
80% nn ts0.5 cms 96.7%
80% nn ts0.5 norm 98.2%
80% nn ts1.0 cms 96.7%
80% nn ts1.0 norm 97.8%
80% np ts0.5 cms 96.4%
80% np ts0.5 norm 97.6%
80% np ts1.0 cms 96.8%
80% np ts1.0 norm 97.3%
90% nn ts0.5 cms 96.7%
90% nn ts0.5 norm 98.3%
90% nn ts1.0 cms 96.7%
90% nn ts1.0 norm 98.0%
90% np ts0.5 cms 96.4%
90% np ts0.5 norm 97.8%
90% np ts1.0 cms 96.8%
90% np ts1.0 norm 97.6%
Matrix: bl, Length thr: lt2, Weighted: unW, Intensity thr: it1
% cut gaps AUC
70% nn ts0.5 cms 98.0%
70% nn ts0.5 norm 99.9%
70% nn ts1.0 cms 98.6%
70% nn ts1.0 norm 99.9%
70% np ts0.5 cms 97.4%
70% np ts0.5 norm 98.7%
70% np ts1.0 cms 96.4%
70% np ts1.0 norm 98.5%
80% nn ts0.5 cms 97.5%
80% nn ts0.5 norm 99.8%
80% nn ts1.0 cms 97.6%
80% nn ts1.0 norm 99.9%
80% np ts0.5 cms 96.9%
80% np ts0.5 norm 98.4%
80% np ts1.0 cms 95.5%
80% np ts1.0 norm 98.4%
90% nn ts0.5 cms 97.4%
90% nn ts0.5 norm 99.8%
90% nn ts1.0 cms 97.6%
90% nn ts1.0 norm 99.9%
90% np ts0.5 cms 96.8%
90% np ts0.5 norm 98.5%
90% np ts1.0 cms 94.9%
90% np ts1.0 norm 98.4%
Matrix: bl, Length thr: lt2, Weighted: unW, Intensity thr: it2
% cut gaps AUC
70% nn ts0.5 cms 97.2%
70% nn ts0.5 norm 98.5%
70% nn ts1.0 cms 97.3%
70% nn ts1.0 norm 98.5%
70% np ts0.5 cms 96.8%
70% np ts0.5 norm 97.9%
70% np ts1.0 cms 97.1%
70% np ts1.0 norm 98.0%
80% nn ts0.5 cms 97.1%
80% nn ts0.5 norm 98.5%
80% nn ts1.0 cms 97.2%
80% nn ts1.0 norm 98.3%
80% np ts0.5 cms 96.5%
80% np ts0.5 norm 97.9%
80% np ts1.0 cms 97.0%
80% np ts1.0 norm 97.7%
90% nn ts0.5 cms 96.9%
90% nn ts0.5 norm 98.6%
90% nn ts1.0 cms 97.0%
90% nn ts1.0 norm 98.4%
90% np ts0.5 cms 96.5%
90% np ts0.5 norm 98.0%
90% np ts1.0 cms 97.0%
90% np ts1.0 norm 97.8%
Matrix: bl, Length thr: lt3, Weighted: pairW, Intensity thr: it1
% cut gaps AUC
70% nn ts0.5 cms 97.9%
70% nn ts0.5 norm 99.2%
70% nn ts1.0 cms 97.1%
70% nn ts1.0 norm 99.5%
70% np ts0.5 cms 96.1%
70% np ts0.5 norm 97.3%
70% np ts1.0 cms 93.0%
70% np ts1.0 norm 97.4%
80% nn ts0.5 cms 96.2%
80% nn ts0.5 norm 98.9%
80% nn ts1.0 cms 95.9%
80% nn ts1.0 norm 99.2%
80% np ts0.5 cms 92.6%
80% np ts0.5 norm 96.2%
80% np ts1.0 cms 89.0%
80% np ts1.0 norm 97.2%
90% nn ts0.5 cms 95.7%
90% nn ts0.5 norm 98.9%
90% nn ts1.0 cms 96.2%
90% nn ts1.0 norm 99.3%
90% np ts0.5 cms 90.7%
90% np ts0.5 norm 95.7%
90% np ts1.0 cms 91.5%
90% np ts1.0 norm 97.2%
Matrix: bl, Length thr: lt3, Weighted: pairW, Intensity thr: it2
% cut gaps AUC
70% nn ts0.5 cms 97.4%
70% nn ts0.5 norm 96.7%
70% nn ts1.0 cms 93.2%
70% nn ts1.0 norm 97.6%
70% np ts0.5 cms 96.9%
70% np ts0.5 norm 96.0%
70% np ts1.0 cms 92.6%
70% np ts1.0 norm 97.2%
80% nn ts0.5 cms 96.7%
80% nn ts0.5 norm 96.0%
80% nn ts1.0 cms 90.9%
80% nn ts1.0 norm 97.2%
80% np ts0.5 cms 95.9%
80% np ts0.5 norm 95.3%
80% np ts1.0 cms 89.7%
80% np ts1.0 norm 96.8%
90% nn ts0.5 cms 84.5%
90% nn ts0.5 norm 94.7%
90% nn ts1.0 cms 91.3%
90% nn ts1.0 norm 96.8%
90% np ts0.5 cms 89.9%
90% np ts0.5 norm 93.6%
90% np ts1.0 cms 90.3%
90% np ts1.0 norm 96.1%
Matrix: bl, Length thr: lt3, Weighted: unW, Intensity thr: it1
% cut gaps AUC
70% nn ts0.5 cms 97.2%
70% nn ts0.5 norm 99.3%
70% nn ts1.0 cms 97.9%
70% nn ts1.0 norm 99.4%
70% np ts0.5 cms 94.7%
70% np ts0.5 norm 96.9%
70% np ts1.0 cms 94.5%
70% np ts1.0 norm 97.4%
80% nn ts0.5 cms 95.1%
80% nn ts0.5 norm 99.0%
80% nn ts1.0 cms 96.6%
80% nn ts1.0 norm 99.3%
80% np ts0.5 cms 92.6%
80% np ts0.5 norm 95.8%
80% np ts1.0 cms 90.7%
80% np ts1.0 norm 97.2%
90% nn ts0.5 cms 94.9%
90% nn ts0.5 norm 98.9%
90% nn ts1.0 cms 95.9%
90% nn ts1.0 norm 99.4%
90% np ts0.5 cms 89.2%
90% np ts0.5 norm 95.7%
90% np ts1.0 cms 90.9%
90% np ts1.0 norm 97.2%
Matrix: bl, Length thr: lt3, Weighted: unW, Intensity thr: it2
% cut gaps AUC
70% nn ts0.5 cms 97.1%
70% nn ts0.5 norm 97.0%
70% nn ts1.0 cms 92.9%
70% nn ts1.0 norm 97.7%
70% np ts0.5 cms 96.6%
70% np ts0.5 norm 96.2%
70% np ts1.0 cms 92.1%
70% np ts1.0 norm 97.3%
80% nn ts0.5 cms 96.4%
80% nn ts0.5 norm 96.2%
80% nn ts1.0 cms 91.9%
80% nn ts1.0 norm 97.3%
80% np ts0.5 cms 95.7%
80% np ts0.5 norm 95.5%
80% np ts1.0 cms 91.0%
80% np ts1.0 norm 97.1%
90% nn ts0.5 cms 88.0%
90% nn ts0.5 norm 94.2%
90% nn ts1.0 cms 92.6%
90% nn ts1.0 norm 96.7%
90% np ts0.5 cms 86.6%
90% np ts0.5 norm 93.5%
90% np ts1.0 cms 91.6%
90% np ts1.0 norm 96.3%
Matrix: lg, Length thr: lt2, Weighted: pairW, Intensity thr: it1
% cut gaps AUC
70% nn ts0.5 cms 98.5%
70% nn ts0.5 norm 99.7%
70% nn ts1.0 cms 98.5%
70% nn ts1.0 norm 99.7%
70% np ts0.5 cms 97.1%
70% np ts0.5 norm 96.7%
70% np ts1.0 cms 94.7%
70% np ts1.0 norm 96.1%
80% nn ts0.5 cms 98.3%
80% nn ts0.5 norm 99.7%
80% nn ts1.0 cms 97.7%
80% nn ts1.0 norm 99.4%
80% np ts0.5 cms 96.8%
80% np ts0.5 norm 96.0%
80% np ts1.0 cms 93.6%
80% np ts1.0 norm 95.6%
90% nn ts0.5 cms 97.7%
90% nn ts0.5 norm 99.6%
90% nn ts1.0 cms 97.6%
90% nn ts1.0 norm 99.4%
90% np ts0.5 cms 96.4%
90% np ts0.5 norm 96.0%
90% np ts1.0 cms 93.6%
90% np ts1.0 norm 95.7%
Matrix: lg, Length thr: lt2, Weighted: pairW, Intensity thr: it2
% cut gaps AUC
70% nn ts0.5 cms 97.3%
70% nn ts0.5 norm 97.8%
70% nn ts1.0 cms 96.8%
70% nn ts1.0 norm 97.8%
70% np ts0.5 cms 95.9%
70% np ts0.5 norm 96.1%
70% np ts1.0 cms 96.2%
70% np ts1.0 norm 96.0%
80% nn ts0.5 cms 97.1%
80% nn ts0.5 norm 97.8%
80% nn ts1.0 cms 96.5%
80% nn ts1.0 norm 97.7%
80% np ts0.5 cms 95.7%
80% np ts0.5 norm 96.0%
80% np ts1.0 cms 96.0%
80% np ts1.0 norm 96.0%
90% nn ts0.5 cms 97.1%
90% nn ts0.5 norm 97.9%
90% nn ts1.0 cms 96.4%
90% nn ts1.0 norm 97.8%
90% np ts0.5 cms 95.8%
90% np ts0.5 norm 96.4%
90% np ts1.0 cms 95.7%
90% np ts1.0 norm 96.3%
Matrix: lg, Length thr: lt2, Weighted: unW, Intensity thr: it1
% cut gaps AUC
70% nn ts0.5 cms 98.2%
70% nn ts0.5 norm 99.8%
70% nn ts1.0 cms 99.0%
70% nn ts1.0 norm 99.7%
70% np ts0.5 cms 97.1%
70% np ts0.5 norm 96.8%
70% np ts1.0 cms 94.8%
70% np ts1.0 norm 96.4%
80% nn ts0.5 cms 98.3%
80% nn ts0.5 norm 99.6%
80% nn ts1.0 cms 97.9%
80% nn ts1.0 norm 99.4%
80% np ts0.5 cms 96.5%
80% np ts0.5 norm 96.1%
80% np ts1.0 cms 94.3%
80% np ts1.0 norm 95.8%
90% nn ts0.5 cms 97.8%
90% nn ts0.5 norm 99.6%
90% nn ts1.0 cms 98.0%
90% nn ts1.0 norm 99.4%
90% np ts0.5 cms 96.3%
90% np ts0.5 norm 96.3%
90% np ts1.0 cms 94.3%
90% np ts1.0 norm 95.9%
Matrix: lg, Length thr: lt2, Weighted: unW, Intensity thr: it2
% cut gaps AUC
70% nn ts0.5 cms 97.1%
70% nn ts0.5 norm 97.7%
70% nn ts1.0 cms 96.8%
70% nn ts1.0 norm 97.8%
70% np ts0.5 cms 95.5%
70% np ts0.5 norm 96.1%
70% np ts1.0 cms 96.3%
70% np ts1.0 norm 96.0%
80% nn ts0.5 cms 96.7%
80% nn ts0.5 norm 97.7%
80% nn ts1.0 cms 96.5%
80% nn ts1.0 norm 97.7%
80% np ts0.5 cms 95.4%
80% np ts0.5 norm 96.1%
80% np ts1.0 cms 96.2%
80% np ts1.0 norm 96.0%
90% nn ts0.5 cms 97.0%
90% nn ts0.5 norm 97.9%
90% nn ts1.0 cms 96.5%
90% nn ts1.0 norm 97.9%
90% np ts0.5 cms 95.7%
90% np ts0.5 norm 96.2%
90% np ts1.0 cms 96.0%
90% np ts1.0 norm 96.3%
Matrix: lg, Length thr: lt3, Weighted: pairW, Intensity thr: it1
% cut gaps AUC
70% nn ts0.5 cms 95.5%
70% nn ts0.5 norm 99.1%
70% nn ts1.0 cms 97.8%
70% nn ts1.0 norm 99.3%
70% np ts0.5 cms 91.8%
70% np ts0.5 norm 96.0%
70% np ts1.0 cms 91.9%
70% np ts1.0 norm 96.0%
80% nn ts0.5 cms 95.5%
80% nn ts0.5 norm 99.1%
80% nn ts1.0 cms 97.7%
80% nn ts1.0 norm 99.4%
80% np ts0.5 cms 88.1%
80% np ts0.5 norm 95.7%
80% np ts1.0 cms 91.0%
80% np ts1.0 norm 95.8%
90% nn ts0.5 cms 93.1%
90% nn ts0.5 norm 98.8%
90% nn ts1.0 cms 96.8%
90% nn ts1.0 norm 99.5%
90% np ts0.5 cms 84.0%
90% np ts0.5 norm 95.2%
90% np ts1.0 cms 88.5%
90% np ts1.0 norm 95.5%
Matrix: lg, Length thr: lt3, Weighted: pairW, Intensity thr: it2
% cut gaps AUC
70% nn ts0.5 cms 97.9%
70% nn ts0.5 norm 97.5%
70% nn ts1.0 cms 94.6%
70% nn ts1.0 norm 98.6%
70% np ts0.5 cms 97.3%
70% np ts0.5 norm 96.4%
70% np ts1.0 cms 92.6%
70% np ts1.0 norm 97.0%
80% nn ts0.5 cms 97.0%
80% nn ts0.5 norm 96.6%
80% nn ts1.0 cms 93.9%
80% nn ts1.0 norm 98.5%
80% np ts0.5 cms 96.3%
80% np ts0.5 norm 96.2%
80% np ts1.0 cms 91.9%
80% np ts1.0 norm 97.1%
90% nn ts0.5 cms 97.1%
90% nn ts0.5 norm 97.1%
90% nn ts1.0 cms 94.2%
90% nn ts1.0 norm 98.6%
90% np ts0.5 cms 96.3%
90% np ts0.5 norm 96.5%
90% np ts1.0 cms 92.2%
90% np ts1.0 norm 97.3%
Matrix: lg, Length thr: lt3, Weighted: unW, Intensity thr: it1
% cut gaps AUC
70% nn ts0.5 cms 95.3%
70% nn ts0.5 norm 98.9%
70% nn ts1.0 cms 98.6%
70% nn ts1.0 norm 99.4%
70% np ts0.5 cms 91.1%
70% np ts0.5 norm 96.1%
70% np ts1.0 cms 91.9%
70% np ts1.0 norm 95.6%
80% nn ts0.5 cms 94.6%
80% nn ts0.5 norm 99.1%
80% nn ts1.0 cms 98.8%
80% nn ts1.0 norm 99.4%
80% np ts0.5 cms 87.1%
80% np ts0.5 norm 96.3%
80% np ts1.0 cms 90.9%
80% np ts1.0 norm 95.8%
90% nn ts0.5 cms 94.3%
90% nn ts0.5 norm 98.9%
90% nn ts1.0 cms 98.0%
90% nn ts1.0 norm 99.5%
90% np ts0.5 cms 86.6%
90% np ts0.5 norm 95.8%
90% np ts1.0 cms 91.5%
90% np ts1.0 norm 95.9%
Matrix: lg, Length thr: lt3, Weighted: unW, Intensity thr: it2
% cut gaps AUC
70% nn ts0.5 cms 97.9%
70% nn ts0.5 norm 97.9%
70% nn ts1.0 cms 93.0%
70% nn ts1.0 norm 98.8%
70% np ts0.5 cms 97.4%
70% np ts0.5 norm 96.7%
70% np ts1.0 cms 90.9%
70% np ts1.0 norm 97.5%
80% nn ts0.5 cms 96.9%
80% nn ts0.5 norm 96.6%
80% nn ts1.0 cms 91.9%
80% nn ts1.0 norm 98.5%
80% np ts0.5 cms 96.2%
80% np ts0.5 norm 95.8%
80% np ts1.0 cms 89.9%
80% np ts1.0 norm 97.1%
90% nn ts0.5 cms 96.9%
90% nn ts0.5 norm 96.8%
90% nn ts1.0 cms 92.1%
90% nn ts1.0 norm 98.6%
90% np ts0.5 cms 96.0%
90% np ts0.5 norm 96.0%
90% np ts1.0 cms 89.2%
90% np ts1.0 norm 97.3%
True positive rate
False positive rate
0.0
0.2
0.4
0.6
0.8
1.0

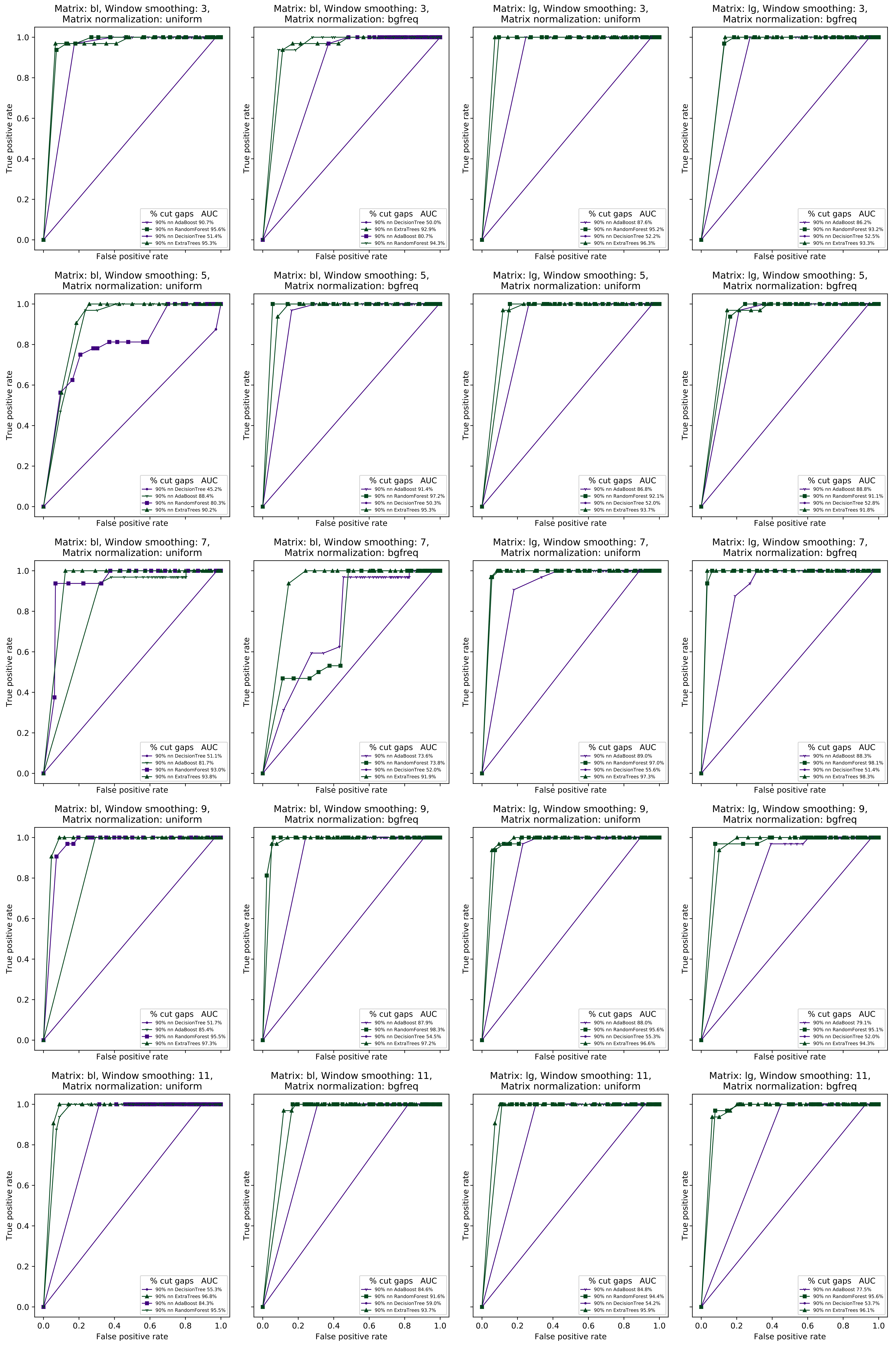

Matrix: bl, Window smoothing: 3, Matrix normalization: uniform
% cut gaps AUC
90% nn AdaBoost 90.7%
90% nn RandomForest 95.6%
90% nn DecisionTree 51.4%
90% nn ExtraTrees 95.3%
Matrix: bl, Window smoothing: 3, Matrix normalization: bgfreq
% cut gaps AUC
90% nn DecisionTree 50.0%
90% nn ExtraTrees 92.9%
90% nn AdaBoost 80.7%
90% nn RandomForest 94.3%
Matrix: lg, Window smoothing: 3, Matrix normalization: uniform
% cut gaps AUC
90% nn AdaBoost 87.6%
90% nn RandomForest 95.2%
90% nn DecisionTree 52.2%
90% nn ExtraTrees 96.3%
Matrix: lg, Window smoothing: 3, Matrix normalization: bgfreq
% cut gaps AUC
90% nn AdaBoost 86.2%
90% nn RandomForest 93.2%
90% nn DecisionTree 52.5%
90% nn ExtraTrees 93.3%
Matrix: bl, Window smoothing: 5, Matrix normalization: uniform
% cut gaps AUC
90% nn DecisionTree 45.2%
90% nn AdaBoost 88.4%
90% nn RandomForest 80.3%
90% nn ExtraTrees 90.2%
Matrix: bl, Window smoothing: 5, Matrix normalization: bgfreq
% cut gaps AUC
90% nn AdaBoost 91.4%
90% nn RandomForest 97.2%
90% nn DecisionTree 50.3%
90% nn ExtraTrees 95.3%
Matrix: lg, Window smoothing: 5, Matrix normalization: uniform
% cut gaps AUC
90% nn AdaBoost 86.8%
90% nn RandomForest 92.1%
90% nn DecisionTree 52.0%
90% nn ExtraTrees 93.7%
Matrix: lg, Window smoothing: 5, Matrix normalization: bgfreq
% cut gaps AUC
90% nn AdaBoost 88.8%
90% nn RandomForest 91.1%
90% nn DecisionTree 52.8%
90% nn ExtraTrees 91.8%
Matrix: bl, Window smoothing: 7, Matrix normalization: uniform
% cut gaps AUC
90% nn DecisionTree 51.1%
90% nn AdaBoost 81.7%
90% nn RandomForest 93.0%
90% nn ExtraTrees 93.8%
Matrix: bl, Window smoothing: 7, Matrix normalization: bgfreq
% cut gaps AUC
90% nn AdaBoost 73.6%
90% nn RandomForest 73.8%
90% nn DecisionTree 52.0%
90% nn ExtraTrees 91.9%
Matrix: lg, Window smoothing: 7, Matrix normalization: uniform
% cut gaps AUC
90% nn AdaBoost 89.0%
90% nn RandomForest 97.0%
90% nn DecisionTree 55.6%
90% nn ExtraTrees 97.3%
Matrix: lg, Window smoothing: 7, Matrix normalization: bgfreq
% cut gaps AUC
90% nn AdaBoost 88.3%
90% nn RandomForest 98.1%
90% nn DecisionTree 51.4%
90% nn ExtraTrees 98.3%
Matrix: bl, Window smoothing: 9, Matrix normalization: uniform
% cut gaps AUC
90% nn DecisionTree 51.7%
90% nn AdaBoost 85.4%
90% nn RandomForest 95.5%
90% nn ExtraTrees 97.3%
Matrix: bl, Window smoothing: 9, Matrix normalization: bgfreq
% cut gaps AUC
90% nn AdaBoost 87.9%
90% nn RandomForest 98.3%
90% nn DecisionTree 54.5%
90% nn ExtraTrees 97.2%
Matrix: lg, Window smoothing: 9, Matrix normalization: uniform
% cut gaps AUC
90% nn AdaBoost 88.0%
90% nn RandomForest 95.6%
90% nn DecisionTree 55.3%
90% nn ExtraTrees 96.6%
Matrix: lg, Window smoothing: 9, Matrix normalization: bgfreq
% cut gaps AUC
90% nn AdaBoost 79.1%
90% nn RandomForest 95.1%
90% nn DecisionTree 52.0%
90% nn ExtraTrees 94.3%
Matrix: bl, Window smoothing: 11, Matrix normalization: uniform
% cut gaps AUC
90% nn DecisionTree 55.3%
90% nn ExtraTrees 96.8%
90% nn AdaBoost 84.3%
90% nn RandomForest 95.5%
Matrix: bl, Window smoothing: 11, Matrix normalization: bgfreq
% cut gaps AUC
90% nn AdaBoost 84.6%
90% nn RandomForest 91.6%
90% nn DecisionTree 59.0%
90% nn ExtraTrees 93.7%
Matrix: lg, Window smoothing: 11, Matrix normalization: uniform
% cut gaps AUC
90% nn AdaBoost 84.8%
90% nn RandomForest 94.4%
90% nn DecisionTree 54.2%
90% nn ExtraTrees 95.9%
Matrix: lg, Window smoothing: 11, Matrix normalization: bgfreq
% cut gaps AUC
90% nn AdaBoost 77.5%
90% nn RandomForest 95.6%
90% nn DecisionTree 53.7%
90% nn ExtraTrees 96.1%
True positive rate
False positive rate

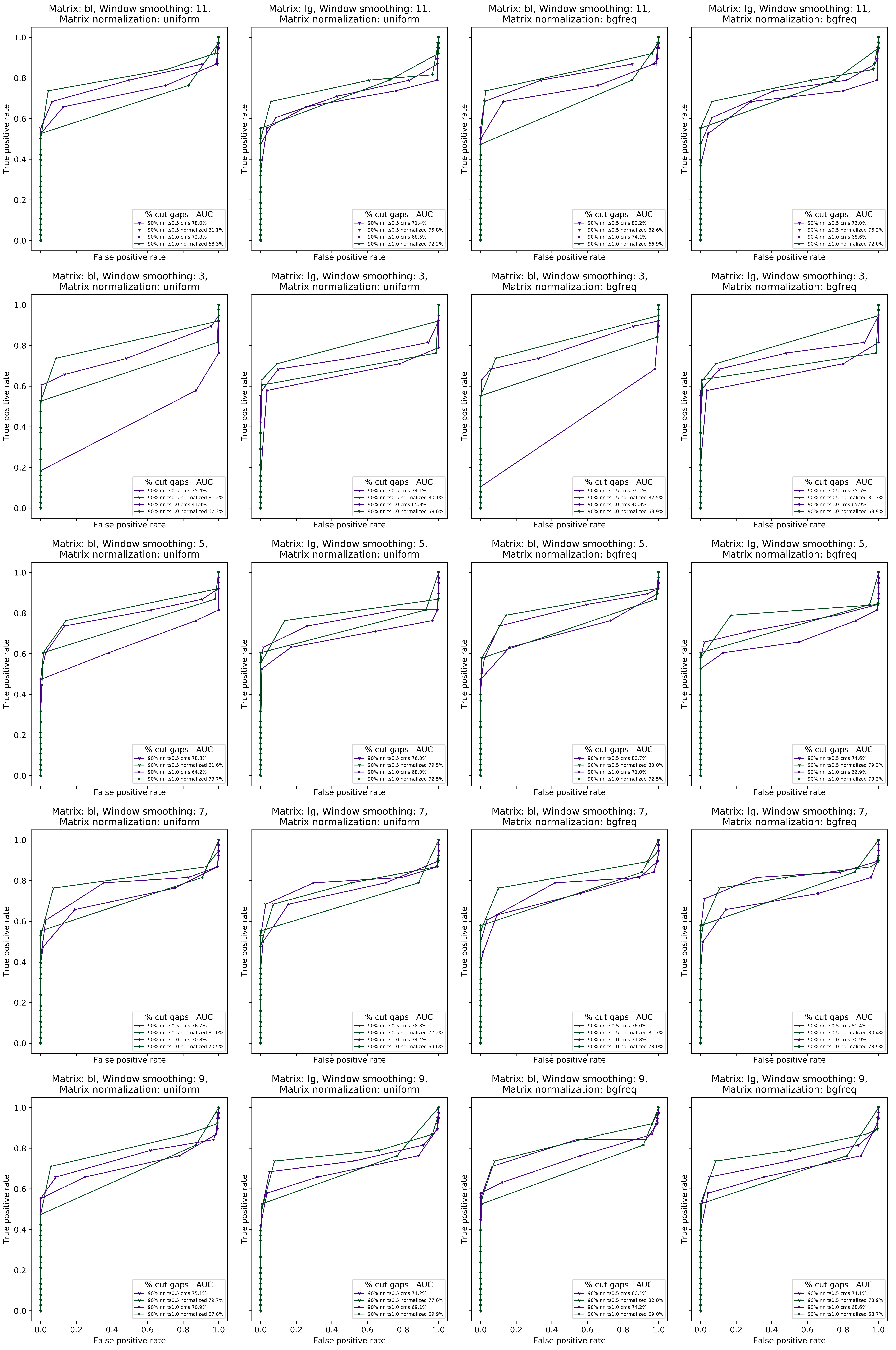
Matrix: bl, Window smoothing: 11, Matrix normalization: uniform
% cut gaps AUC
90% nn ts0.5 cms 78.0%
90% nn ts0.5 normalized 81.1%
90% nn ts1.0 cms 72.8%
90% nn ts1.0 normalized 68.3%
Matrix: lg, Window smoothing: 11, Matrix normalization: uniform
% cut gaps AUC
90% nn ts0.5 cms 71.4%
90% nn ts0.5 normalized 75.8%
90% nn ts1.0 cms 68.5%
90% nn ts1.0 normalized 72.2%
Matrix: bl, Window smoothing: 11, Matrix normalization: bgfreq
% cut gaps AUC
90% nn ts0.5 cms 80.2%
90% nn ts0.5 normalized 82.6%
90% nn ts1.0 cms 74.1%
90% nn ts1.0 normalized 66.9%
Matrix: lg, Window smoothing: 11, Matrix normalization: bgfreq
% cut gaps AUC
90% nn ts0.5 cms 73.0%
90% nn ts0.5 normalized 76.2%
90% nn ts1.0 cms 68.6%
90% nn ts1.0 normalized 72.0%
Matrix: bl, Window smoothing: 3, Matrix normalization: uniform
% cut gaps AUC
90% nn ts0.5 cms 75.4%
90% nn ts0.5 normalized 81.2%
90% nn ts1.0 cms 41.9%
90% nn ts1.0 normalized 67.3%
Matrix: lg, Window smoothing: 3, Matrix normalization: uniform
% cut gaps AUC
90% nn ts0.5 cms 74.1%
90% nn ts0.5 normalized 80.1%
90% nn ts1.0 cms 65.8%
90% nn ts1.0 normalized 68.6%
Matrix: bl, Window smoothing: 3, Matrix normalization: bgfreq
% cut gaps AUC
90% nn ts0.5 cms 79.1%
90% nn ts0.5 normalized 82.5%
90% nn ts1.0 cms 40.3%
90% nn ts1.0 normalized 69.9%
Matrix: lg, Window smoothing: 3, Matrix normalization: bgfreq
% cut gaps AUC
90% nn ts0.5 cms 75.5%
90% nn ts0.5 normalized 81.3%
90% nn ts1.0 cms 65.9%
90% nn ts1.0 normalized 69.9%
Matrix: bl, Window smoothing: 5, Matrix normalization: uniform
% cut gaps AUC
90% nn ts0.5 cms 78.8%
90% nn ts0.5 normalized 81.6%
90% nn ts1.0 cms 64.2%
90% nn ts1.0 normalized 73.7%
Matrix: lg, Window smoothing: 5, Matrix normalization: uniform
% cut gaps AUC
90% nn ts0.5 cms 76.0%
90% nn ts0.5 normalized 79.5%
90% nn ts1.0 cms 68.0%
90% nn ts1.0 normalized 72.5%
Matrix: bl, Window smoothing: 5, Matrix normalization: bgfreq
% cut gaps AUC
90% nn ts0.5 cms 80.7%
90% nn ts0.5 normalized 83.0%
90% nn ts1.0 cms 71.0%
90% nn ts1.0 normalized 72.5%
Matrix: lg, Window smoothing: 5, Matrix normalization: bgfreq
% cut gaps AUC
90% nn ts0.5 cms 74.6%
90% nn ts0.5 normalized 79.3%
90% nn ts1.0 cms 66.9%
90% nn ts1.0 normalized 73.3%
Matrix: bl, Window smoothing: 7, Matrix normalization: uniform
% cut gaps AUC
90% nn ts0.5 cms 76.7%
90% nn ts0.5 normalized 81.0%
90% nn ts1.0 cms 70.8%
90% nn ts1.0 normalized 70.5%
Matrix: lg, Window smoothing: 7, Matrix normalization: uniform
% cut gaps AUC
90% nn ts0.5 cms 78.8%
90% nn ts0.5 normalized 77.2%
90% nn ts1.0 cms 74.4%
90% nn ts1.0 normalized 69.6%
Matrix: bl, Window smoothing: 7, Matrix normalization: bgfreq
% cut gaps AUC
90% nn ts0.5 cms 76.0%
90% nn ts0.5 normalized 81.7%
90% nn ts1.0 cms 71.8%
90% nn ts1.0 normalized 73.0%
Matrix: lg, Window smoothing: 7, Matrix normalization: bgfreq
% cut gaps AUC
90% nn ts0.5 cms 81.4%
90% nn ts0.5 normalized 80.4%
90% nn ts1.0 cms 70.9%
90% nn ts1.0 normalized 73.9%
Matrix: bl, Window smoothing: 9, Matrix normalization: uniform
% cut gaps AUC
90% nn ts0.5 cms 75.1%
90% nn ts0.5 normalized 79.7%
90% nn ts1.0 cms 70.9%
90% nn ts1.0 normalized 67.8%
Matrix: lg, Window smoothing: 9, Matrix normalization: uniform
% cut gaps AUC
90% nn ts0.5 cms 74.2%
90% nn ts0.5 normalized 77.6%
90% nn ts1.0 cms 69.1%
90% nn ts1.0 normalized 69.9%
Matrix: bl, Window smoothing: 9, Matrix normalization: bgfreq
% cut gaps AUC
90% nn ts0.5 cms 80.1%
90% nn ts0.5 normalized 82.0%
90% nn ts1.0 cms 74.2%
90% nn ts1.0 normalized 69.0%
Matrix: lg, Window smoothing: 9, Matrix normalization: bgfreq
% cut gaps AUC
90% nn ts0.5 cms 74.1%
90% nn ts0.5 normalized 78.9%
90% nn ts1.0 cms 68.6%
90% nn ts1.0 normalized 68.7%
True positive rate
False positive rate

PRST vs BBS

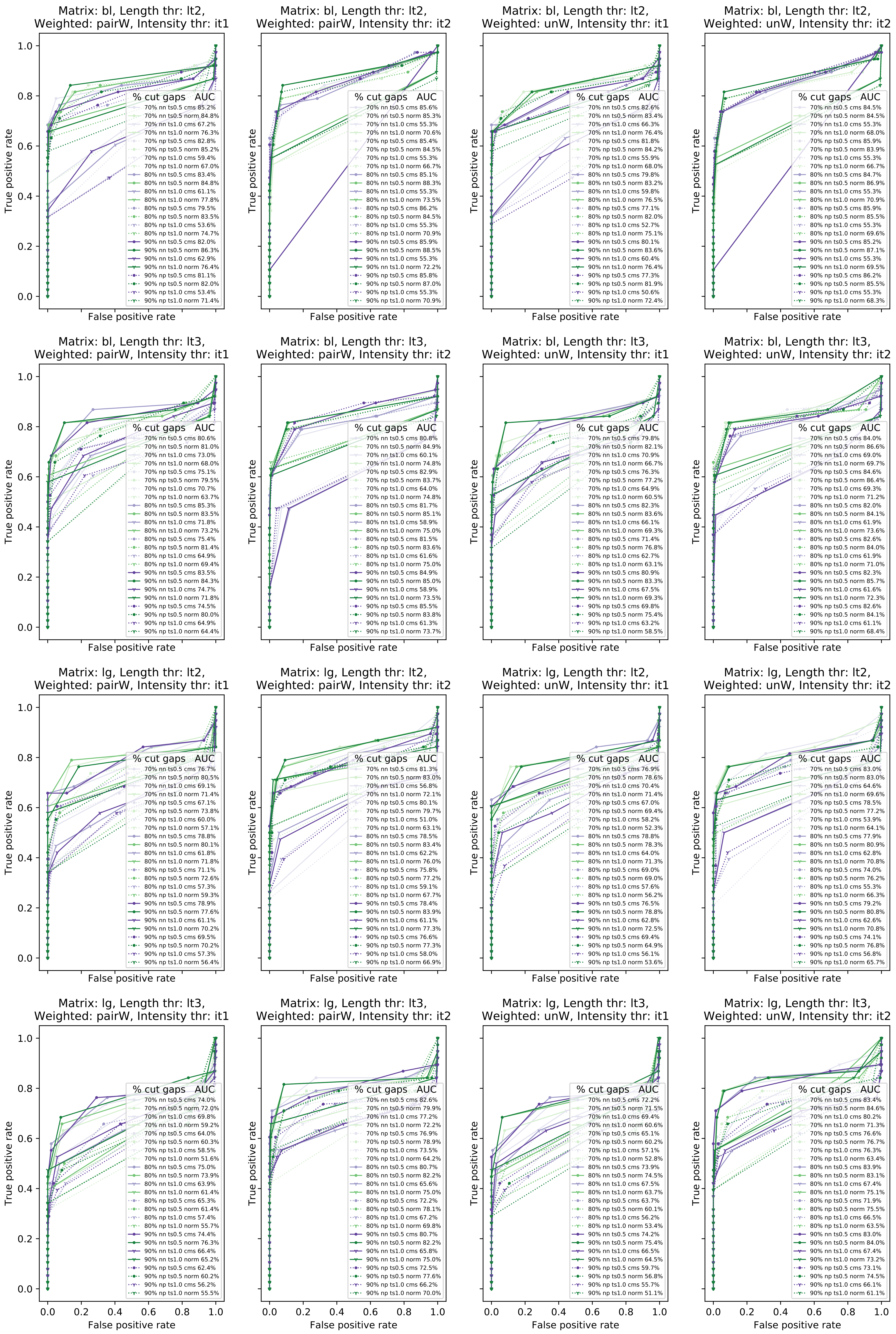

Matrix: bl, Length thr: lt2, Weighted: pairW, Intensity thr: it1
Matrix: bl, Length thr: lt2, Weighted: pairW, Intensity thr: it2
Matrix: bl, Length thr: lt2, Weighted: unW, Intensity thr: it1
Matrix: bl, Length thr: lt2, Weighted: unW, Intensity thr: it2
Matrix: bl, Length thr: lt3, Weighted: pairW, Intensity thr: it1
Matrix: bl, Length thr: lt3, Weighted: pairW, Intensity thr: it2
Matrix: bl, Length thr: lt3, Weighted: unW, Intensity thr: it1
Matrix: bl, Length thr: lt3, Weighted: unW, Intensity thr: it2
Matrix: lg, Length thr: lt2, Weighted: pairW, Intensity thr: it1
Matrix: lg, Length thr: lt2, Weighted: pairW, Intensity thr: it2
Matrix: lg, Length thr: lt2, Weighted: unW, Intensity thr: it1
Matrix: lg, Length thr: lt2, Weighted: unW, Intensity thr: it2
Matrix: lg, Length thr: lt3, Weighted: pairW, Intensity thr: it1
Matrix: lg, Length thr: lt3, Weighted: pairW, Intensity thr: it2
Matrix: lg, Length thr: lt3, Weighted: unW, Intensity thr: it1
Matrix: lg, Length thr: lt3, Weighted: unW, Intensity thr: it2
% cut gaps AUC
True positive rate
False positive rate

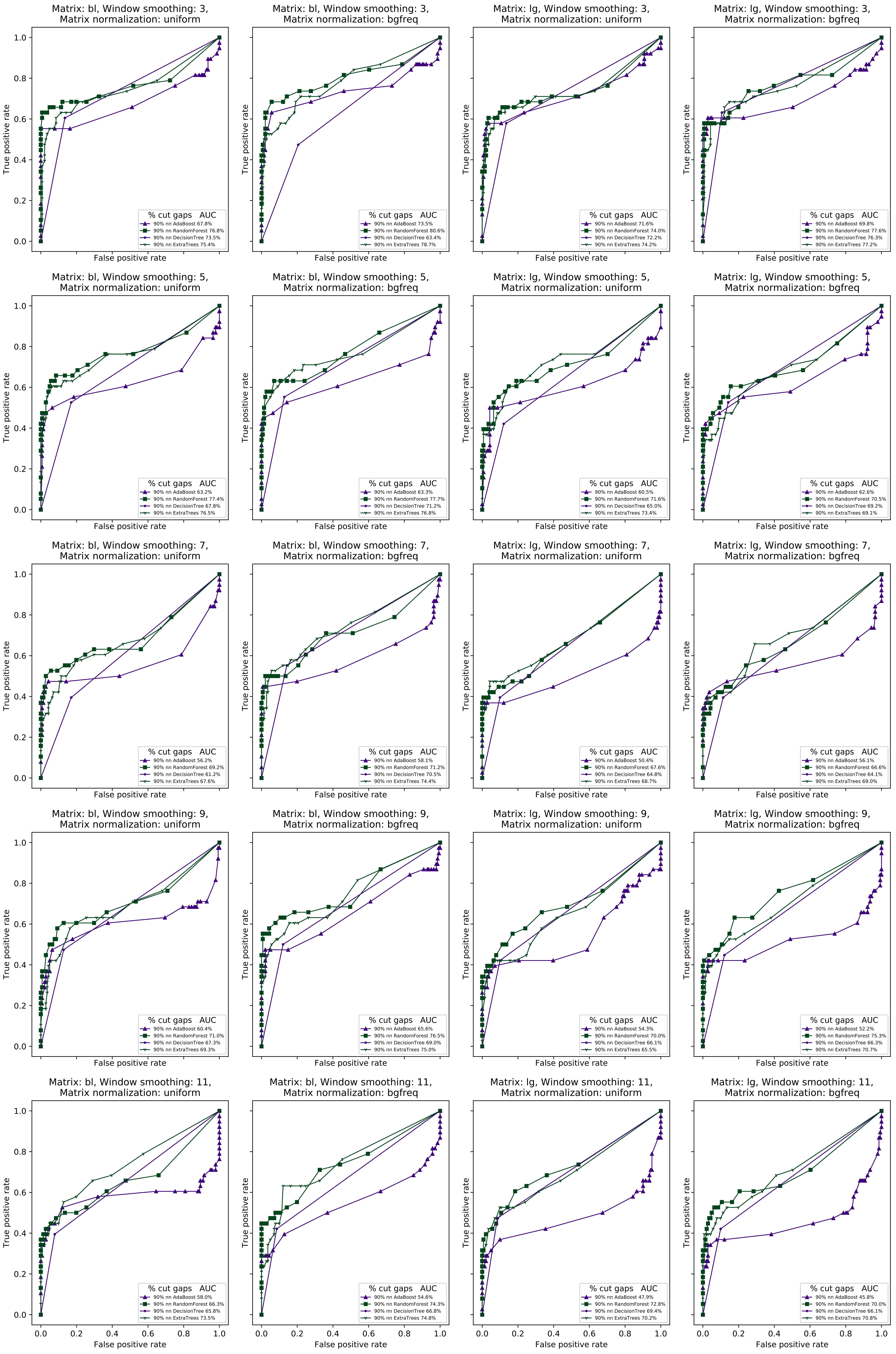

Matrix: bl, Window smoothing: 3, Matrix normalization: uniform
% cut gaps AUC
90% nn AdaBoost 67.8%
90% nn RandomForest 76.8%
90% nn DecisionTree 73.5%
90% nn ExtraTrees 75.4%
Matrix: bl, Window smoothing: 3, Matrix normalization: bgfreq
% cut gaps AUC
90% nn AdaBoost 73.5%
90% nn RandomForest 80.6%
90% nn DecisionTree 63.4%
90% nn ExtraTrees 78.7%
Matrix: lg, Window smoothing: 3, Matrix normalization: uniform
% cut gaps AUC
90% nn AdaBoost 71.6%
90% nn RandomForest 74.0%
90% nn DecisionTree 72.2%
90% nn ExtraTrees 74.2%
Matrix: lg, Window smoothing: 3, Matrix normalization: bgfreq
% cut gaps AUC
90% nn AdaBoost 69.8%
90% nn RandomForest 77.6%
90% nn DecisionTree 76.3%
90% nn ExtraTrees 77.2%
Matrix: bl, Window smoothing: 5, Matrix normalization: uniform
% cut gaps AUC
90% nn AdaBoost 63.2%
90% nn RandomForest 77.4%
90% nn DecisionTree 67.8%
90% nn ExtraTrees 76.5%
Matrix: bl, Window smoothing: 5, Matrix normalization: bgfreq
% cut gaps AUC
90% nn AdaBoost 63.3%
90% nn RandomForest 77.7%
90% nn DecisionTree 71.2%
90% nn ExtraTrees 76.8%
Matrix: lg, Window smoothing: 5, Matrix normalization: uniform
% cut gaps AUC
90% nn AdaBoost 60.5%
90% nn RandomForest 71.6%
90% nn DecisionTree 65.0%
90% nn ExtraTrees 73.4%
Matrix: lg, Window smoothing: 5, Matrix normalization: bgfreq
% cut gaps AUC
90% nn AdaBoost 62.6%
90% nn RandomForest 70.5%
90% nn DecisionTree 69.2%
90% nn ExtraTrees 69.1%
Matrix: bl, Window smoothing: 7, Matrix normalization: uniform
% cut gaps AUC
90% nn AdaBoost 56.2%
90% nn RandomForest 69.2%
90% nn DecisionTree 61.2%
90% nn ExtraTrees 67.6%
Matrix: bl, Window smoothing: 7, Matrix normalization: bgfreq
% cut gaps AUC
90% nn AdaBoost 58.1%
90% nn RandomForest 71.2%
90% nn DecisionTree 70.5%
90% nn ExtraTrees 74.4%
Matrix: lg, Window smoothing: 7, Matrix normalization: uniform
% cut gaps AUC
90% nn AdaBoost 50.4%
90% nn RandomForest 67.6%
90% nn DecisionTree 64.8%
90% nn ExtraTrees 68.7%
Matrix: lg, Window smoothing: 7, Matrix normalization: bgfreq
% cut gaps AUC
90% nn AdaBoost 56.1%
90% nn RandomForest 66.6%
90% nn DecisionTree 64.1%
90% nn ExtraTrees 69.0%
Matrix: bl, Window smoothing: 9, Matrix normalization: uniform
% cut gaps AUC
90% nn AdaBoost 60.4%
90% nn RandomForest 71.0%
90% nn DecisionTree 67.3%
90% nn ExtraTrees 69.3%
Matrix: bl, Window smoothing: 9, Matrix normalization: bgfreq
% cut gaps AUC
90% nn AdaBoost 65.6%
90% nn RandomForest 76.5%
90% nn DecisionTree 69.0%
90% nn ExtraTrees 75.0%
Matrix: lg, Window smoothing: 9, Matrix normalization: uniform
% cut gaps AUC
90% nn AdaBoost 54.3%
90% nn RandomForest 70.0%
90% nn DecisionTree 66.1%
90% nn ExtraTrees 65.5%
Matrix: lg, Window smoothing: 9, Matrix normalization: bgfreq
% cut gaps AUC
90% nn AdaBoost 52.2%
90% nn RandomForest 75.3%
90% nn DecisionTree 66.3%
90% nn ExtraTrees 70.7%
Matrix: bl, Window smoothing: 11, Matrix normalization: uniform
% cut gaps AUC
90% nn AdaBoost 58.0%
90% nn RandomForest 66.3%
90% nn DecisionTree 65.8%
90% nn ExtraTrees 73.5%
Matrix: bl, Window smoothing: 11, Matrix normalization: bgfreq
% cut gaps AUC
90% nn AdaBoost 54.6%
90% nn RandomForest 74.3%
90% nn DecisionTree 66.8%
90% nn ExtraTrees 74.8%
Matrix: lg, Window smoothing: 11, Matrix normalization: uniform
% cut gaps AUC
90% nn AdaBoost 47.9%
90% nn RandomForest 72.8%
90% nn DecisionTree 69.4%
90% nn ExtraTrees 70.2%
Matrix: lg, Window smoothing: 11, Matrix normalization: bgfreq
% cut gaps AUC
90% nn AdaBoost 45.8%
90% nn RandomForest 70.0%
90% nn DecisionTree 66.1%
90% nn ExtraTrees 70.8%
True positive rate
False positive rate

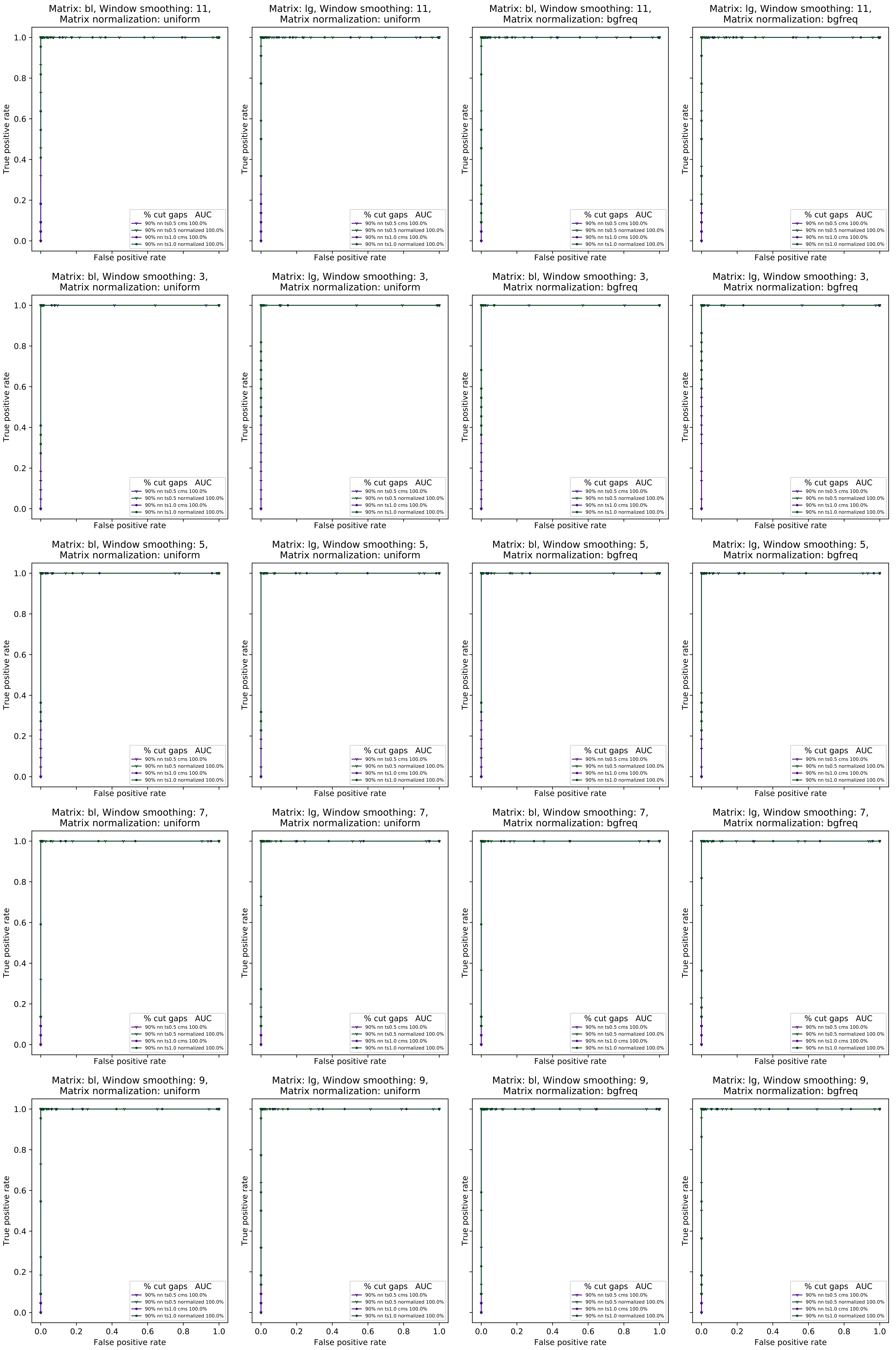

Matrix: bl, Window smoothing: 11, Matrix normalization: uniform
Matrix: lg, Window smoothing: 11, Matrix normalization: uniform
Matrix: bl, Window smoothing: 11, Matrix normalization: bgfreq
Matrix: lg, Window smoothing: 11, Matrix normalization: bgfreq
Matrix: bl, Window smoothing: 3, Matrix normalization: uniform
Matrix: lg, Window smoothing: 3, Matrix normalization: uniform
Matrix: bl, Window smoothing: 3, Matrix normalization: bgfreq
Matrix: lg, Window smoothing: 3, Matrix normalization: bgfreq
Matrix: bl, Window smoothing: 5, Matrix normalization: uniform
Matrix: lg, Window smoothing: 5, Matrix normalization: uniform
Matrix: bl, Window smoothing: 5, Matrix normalization: bgfreq
Matrix: lg, Window smoothing: 5, Matrix normalization: bgfreq
Matrix: bl, Window smoothing: 7, Matrix normalization: uniform
Matrix: lg, Window smoothing: 7, Matrix normalization: uniform
Matrix: bl, Window smoothing: 7, Matrix normalization: bgfreq
Matrix: lg, Window smoothing: 7, Matrix normalization: bgfreq
Matrix: bl, Window smoothing: 9, Matrix normalization: uniform
Matrix: lg, Window smoothing: 9, Matrix normalization: uniform
Matrix: bl, Window smoothing: 9, Matrix normalization: bgfreq
Matrix: lg, Window smoothing: 9, Matrix normalization: bgfreq
True positive rate
False positive rate
% cut gaps AUC
90% nn ts0.5 cms 100.0%
90% nn ts0.5 normalized 100.0%
90% nn ts1.0 cms 100.0%
90% nn ts1.0 normalized 100.0%

PRST vs IND

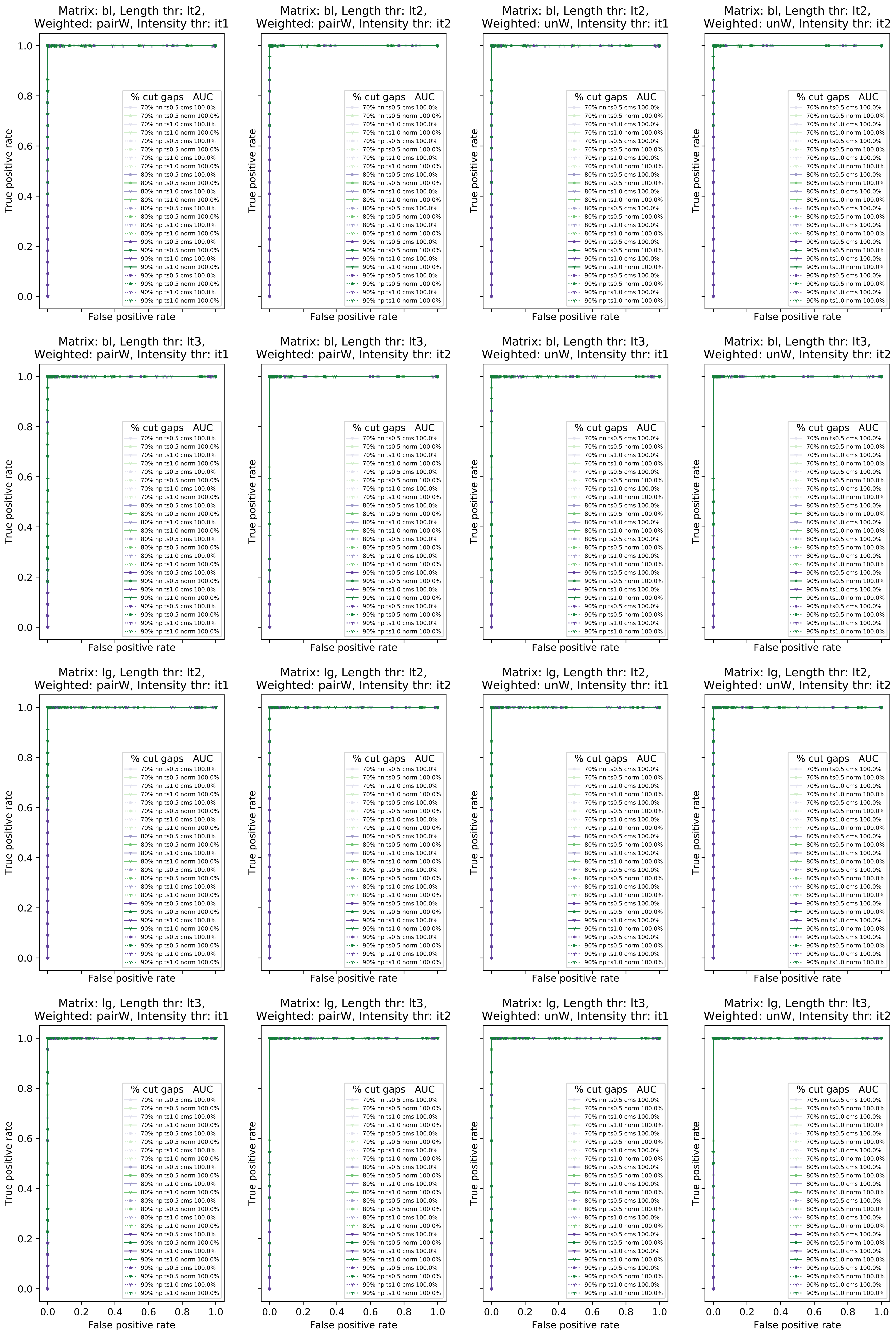

Matrix: bl, Length thr: lt2, Weighted: pairW, Intensity thr: it1
Matrix: bl, Length thr: lt2, Weighted: pairW, Intensity thr: it2
Matrix: bl, Length thr: lt2, Weighted: unW, Intensity thr: it1
Matrix: bl, Length thr: lt2, Weighted: unW, Intensity thr: it2
Matrix: bl, Length thr: lt3, Weighted: pairW, Intensity thr: it1
Matrix: bl, Length thr: lt3, Weighted: pairW, Intensity thr: it2
Matrix: bl, Length thr: lt3, Weighted: unW, Intensity thr: it1
Matrix: bl, Length thr: lt3, Weighted: unW, Intensity thr: it2
Matrix: lg, Length thr: lt2, Weighted: pairW, Intensity thr: it1
Matrix: lg, Length thr: lt2, Weighted: pairW, Intensity thr: it2
Matrix: lg, Length thr: lt2, Weighted: unW, Intensity thr: it1
Matrix: lg, Length thr: lt2, Weighted: unW, Intensity thr: it2
Matrix: lg, Length thr: lt3, Weighted: pairW, Intensity thr: it1
Matrix: lg, Length thr: lt3, Weighted: pairW, Intensity thr: it2
Matrix: lg, Length thr: lt3, Weighted: unW, Intensity thr: it1
Matrix: lg, Length thr: lt3, Weighted: unW, Intensity thr: it2
True positive rate
False positive rate
0.0
0.2
0.4
0.6
0.8
1.0
% cut gaps AUC
70% nn ts0.5 cms 100.0%
70% nn ts0.5 norm 100.0%
70% nn ts1.0 cms 100.0%
70% nn ts1.0 norm 100.0%
70% np ts0.5 cms 100.0%
70% np ts0.5 norm 100.0%
70% np ts1.0 cms 100.0%
70% np ts1.0 norm 100.0%
80% nn ts0.5 cms 100.0%
80% nn ts0.5 norm 100.0%
80% nn ts1.0 cms 100.0%
80% nn ts1.0 norm 100.0%
80% np ts0.5 cms 100.0%
80% np ts0.5 norm 100.0%
80% np ts1.0 cms 100.0%
80% np ts1.0 norm 100.0%
90% nn ts0.5 cms 100.0%
90% nn ts0.5 norm 100.0%
90% nn ts1.0 cms 100.0%
90% nn ts1.0 norm 100.0%
90% np ts0.5 cms 100.0%
90% np ts0.5 norm 100.0%
90% np ts1.0 cms 100.0%
90% np ts1.0 norm 100.0%

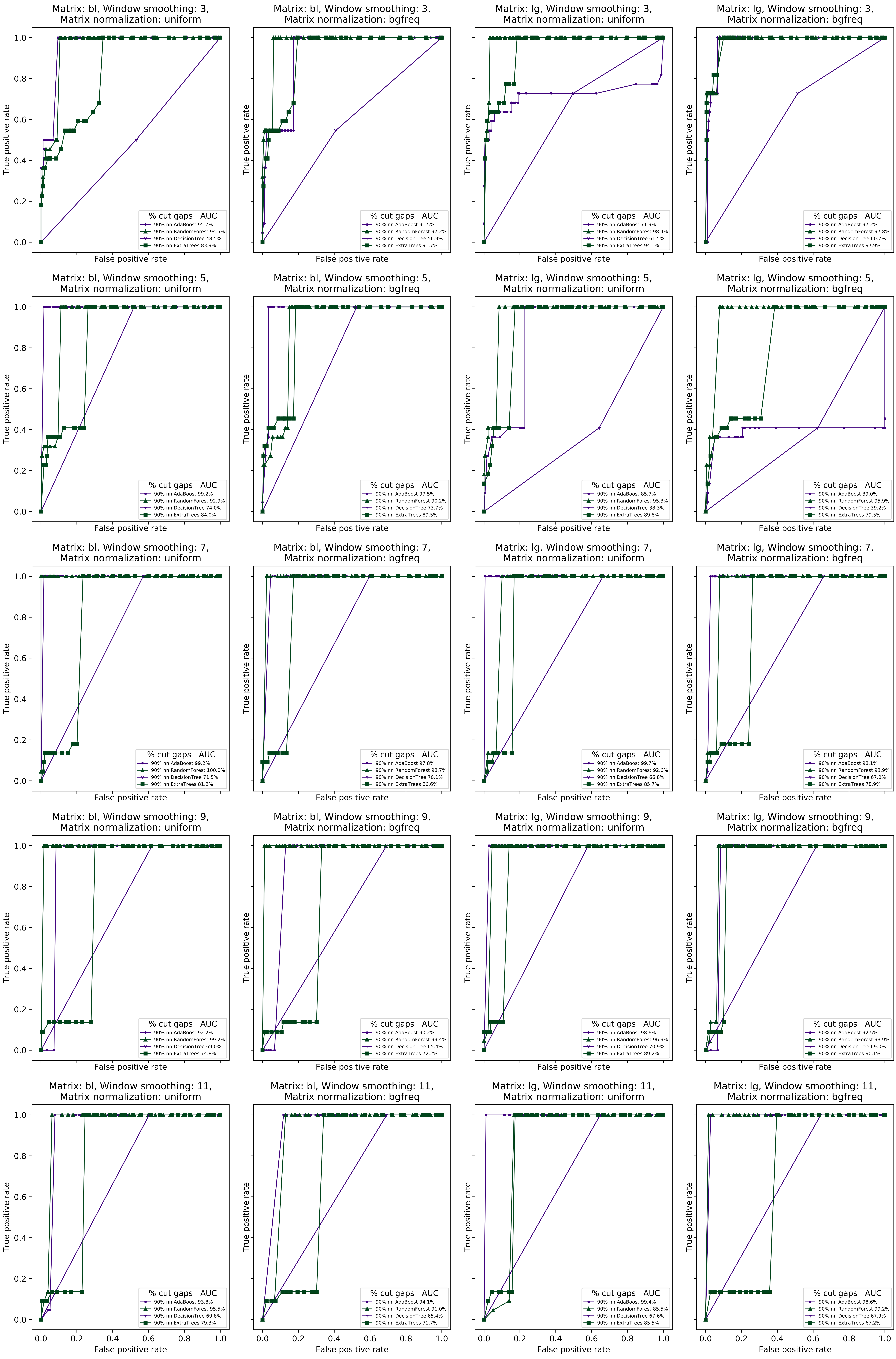

Matrix: bl, Window smoothing: 3, Matrix normalization: uniform
% cut gaps AUC
90% nn AdaBoost 95.7%
90% nn RandomForest 94.5%
90% nn DecisionTree 48.5%
90% nn ExtraTrees 83.9%
Matrix: bl, Window smoothing: 3, Matrix normalization: bgfreq
% cut gaps AUC
90% nn AdaBoost 91.5%
90% nn RandomForest 97.2%
90% nn DecisionTree 56.9%
90% nn ExtraTrees 91.7%
Matrix: lg, Window smoothing: 3, Matrix normalization: uniform
% cut gaps AUC
90% nn AdaBoost 71.9%
90% nn RandomForest 98.4%
90% nn DecisionTree 61.5%
90% nn ExtraTrees 94.1%
Matrix: lg, Window smoothing: 3, Matrix normalization: bgfreq
% cut gaps AUC
90% nn AdaBoost 97.2%
90% nn RandomForest 97.8%
90% nn DecisionTree 60.7%
90% nn ExtraTrees 97.9%
Matrix: bl, Window smoothing: 5, Matrix normalization: uniform
% cut gaps AUC
90% nn AdaBoost 99.2%
90% nn RandomForest 92.9%
90% nn DecisionTree 74.0%
90% nn ExtraTrees 84.0%
Matrix: bl, Window smoothing: 5, Matrix normalization: bgfreq
% cut gaps AUC
90% nn AdaBoost 97.5%
90% nn RandomForest 90.2%
90% nn DecisionTree 73.7%
90% nn ExtraTrees 89.5%
Matrix: lg, Window smoothing: 5, Matrix normalization: uniform
% cut gaps AUC
90% nn AdaBoost 85.7%
90% nn RandomForest 95.3%
90% nn DecisionTree 38.3%
90% nn ExtraTrees 89.8%
Matrix: lg, Window smoothing: 5, Matrix normalization: bgfreq
% cut gaps AUC
90% nn AdaBoost 39.0%
90% nn RandomForest 95.9%
90% nn DecisionTree 39.2%
90% nn ExtraTrees 79.5%
Matrix: bl, Window smoothing: 7, Matrix normalization: uniform
% cut gaps AUC
90% nn AdaBoost 99.2%
90% nn RandomForest 100.0%
90% nn DecisionTree 71.5%
90% nn ExtraTrees 81.2%
Matrix: bl, Window smoothing: 7, Matrix normalization: bgfreq
% cut gaps AUC
90% nn AdaBoost 97.8%
90% nn RandomForest 98.7%
90% nn DecisionTree 70.1%
90% nn ExtraTrees 86.6%
Matrix: lg, Window smoothing: 7, Matrix normalization: uniform
% cut gaps AUC
90% nn AdaBoost 99.7%
90% nn RandomForest 92.6%
90% nn DecisionTree 66.8%
90% nn ExtraTrees 85.7%
Matrix: lg, Window smoothing: 7, Matrix normalization: bgfreq
% cut gaps AUC
90% nn AdaBoost 98.1%
90% nn RandomForest 93.9%
90% nn DecisionTree 67.0%
90% nn ExtraTrees 78.9%
Matrix: bl, Window smoothing: 9, Matrix normalization: uniform
% cut gaps AUC
90% nn AdaBoost 92.2%
90% nn RandomForest 99.2%
90% nn DecisionTree 69.0%
90% nn ExtraTrees 74.8%
Matrix: bl, Window smoothing: 9, Matrix normalization: bgfreq
% cut gaps AUC
90% nn AdaBoost 90.2%
90% nn RandomForest 99.4%
90% nn DecisionTree 65.4%
90% nn ExtraTrees 72.2%
Matrix: lg, Window smoothing: 9, Matrix normalization: uniform
% cut gaps AUC
90% nn AdaBoost 98.6%
90% nn RandomForest 96.9%
90% nn DecisionTree 70.9%
90% nn ExtraTrees 89.2%
Matrix: lg, Window smoothing: 9, Matrix normalization: bgfreq
% cut gaps AUC
90% nn AdaBoost 92.5%
90% nn RandomForest 93.9%
90% nn DecisionTree 69.0%
90% nn ExtraTrees 90.1%
Matrix: bl, Window smoothing: 11, Matrix normalization: uniform
% cut gaps AUC
90% nn AdaBoost 93.8%
90% nn RandomForest 95.5%
90% nn DecisionTree 69.8%
90% nn ExtraTrees 79.3%
Matrix: bl, Window smoothing: 11, Matrix normalization: bgfreq
% cut gaps AUC
90% nn AdaBoost 94.1%
90% nn RandomForest 91.0%
90% nn DecisionTree 65.4%
90% nn ExtraTrees 71.7%
Matrix: lg, Window smoothing: 11, Matrix normalization: uniform
% cut gaps AUC
90% nn AdaBoost 99.4%
90% nn RandomForest 85.5%
90% nn DecisionTree 67.6%
90% nn ExtraTrees 85.5%
Matrix: lg, Window smoothing: 11, Matrix normalization: bgfreq
% cut gaps AUC
90% nn AdaBoost 98.6%
90% nn RandomForest 99.2%
90% nn DecisionTree 67.9%
90% nn ExtraTrees 67.2%
True positive rate
False positive rate

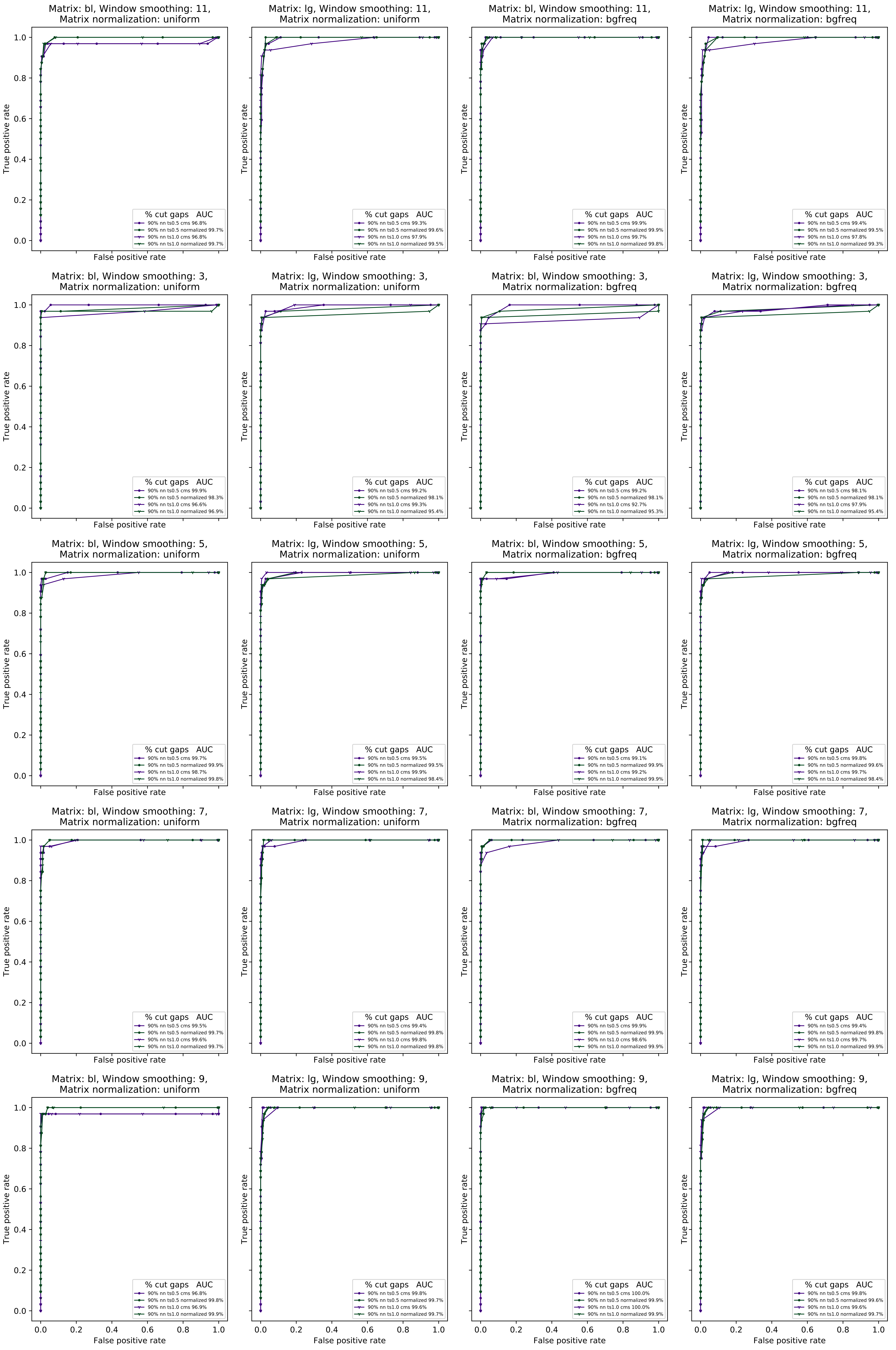
Matrix: bl, Window smoothing: 11, Matrix normalization: uniform
% cut gaps AUC
90% nn ts0.5 cms 96.8%
90% nn ts0.5 normalized 99.7%
90% nn ts1.0 cms 96.8%
90% nn ts1.0 normalized 99.7%
Matrix: lg, Window smoothing: 11, Matrix normalization: uniform
% cut gaps AUC
90% nn ts0.5 cms 99.3%
90% nn ts0.5 normalized 99.6%
90% nn ts1.0 cms 97.9%
90% nn ts1.0 normalized 99.5%
Matrix: bl, Window smoothing: 11, Matrix normalization: bgfreq
% cut gaps AUC
90% nn ts0.5 cms 99.9%
90% nn ts0.5 normalized 99.9%
90% nn ts1.0 cms 99.7%
90% nn ts1.0 normalized 99.8%
Matrix: lg, Window smoothing: 11, Matrix normalization: bgfreq
% cut gaps AUC
90% nn ts0.5 cms 99.4%
90% nn ts0.5 normalized 99.5%
90% nn ts1.0 cms 97.8%
90% nn ts1.0 normalized 99.3%
Matrix: bl, Window smoothing: 3, Matrix normalization: uniform
% cut gaps AUC
90% nn ts0.5 cms 99.9%
90% nn ts0.5 normalized 98.3%
90% nn ts1.0 cms 96.6%
90% nn ts1.0 normalized 96.9%
Matrix: lg, Window smoothing: 3, Matrix normalization: uniform
% cut gaps AUC
90% nn ts0.5 cms 99.2%
90% nn ts0.5 normalized 98.1%
90% nn ts1.0 cms 99.3%
90% nn ts1.0 normalized 95.4%
Matrix: bl, Window smoothing: 3, Matrix normalization: bgfreq
% cut gaps AUC
90% nn ts0.5 cms 99.2%
90% nn ts0.5 normalized 98.1%
90% nn ts1.0 cms 92.7%
90% nn ts1.0 normalized 95.3%
Matrix: lg, Window smoothing: 3, Matrix normalization: bgfreq
% cut gaps AUC
90% nn ts0.5 cms 98.1%
90% nn ts0.5 normalized 98.1%
90% nn ts1.0 cms 97.9%
90% nn ts1.0 normalized 95.4%
Matrix: bl, Window smoothing: 5, Matrix normalization: uniform
% cut gaps AUC
90% nn ts0.5 cms 99.7%
90% nn ts0.5 normalized 99.9%
90% nn ts1.0 cms 98.7%
90% nn ts1.0 normalized 99.8%
Matrix: lg, Window smoothing: 5, Matrix normalization: uniform
% cut gaps AUC
90% nn ts0.5 cms 99.5%
90% nn ts0.5 normalized 99.5%
90% nn ts1.0 cms 99.9%
90% nn ts1.0 normalized 98.4%
Matrix: bl, Window smoothing: 5, Matrix normalization: bgfreq
% cut gaps AUC
90% nn ts0.5 cms 99.1%
90% nn ts0.5 normalized 99.9%
90% nn ts1.0 cms 99.2%
90% nn ts1.0 normalized 99.9%
Matrix: lg, Window smoothing: 5, Matrix normalization: bgfreq
% cut gaps AUC
90% nn ts0.5 cms 99.8%
90% nn ts0.5 normalized 99.6%
90% nn ts1.0 cms 99.7%
90% nn ts1.0 normalized 98.4%
Matrix: bl, Window smoothing: 7, Matrix normalization: uniform
% cut gaps AUC
90% nn ts0.5 cms 99.5%
90% nn ts0.5 normalized 99.7%
90% nn ts1.0 cms 99.6%
90% nn ts1.0 normalized 99.7%
Matrix: lg, Window smoothing: 7, Matrix normalization: uniform
% cut gaps AUC
90% nn ts0.5 cms 99.4%
90% nn ts0.5 normalized 99.8%
90% nn ts1.0 cms 99.8%
90% nn ts1.0 normalized 99.8%
Matrix: bl, Window smoothing: 7, Matrix normalization: bgfreq
% cut gaps AUC
90% nn ts0.5 cms 99.9%
90% nn ts0.5 normalized 99.9%
90% nn ts1.0 cms 98.6%
90% nn ts1.0 normalized 99.9%
Matrix: lg, Window smoothing: 7, Matrix normalization: bgfreq
% cut gaps AUC
90% nn ts0.5 cms 99.4%
90% nn ts0.5 normalized 99.8%
90% nn ts1.0 cms 99.7%
90% nn ts1.0 normalized 99.9%
Matrix: bl, Window smoothing: 9, Matrix normalization: uniform
% cut gaps AUC
90% nn ts0.5 cms 96.8%
90% nn ts0.5 normalized 99.8%
90% nn ts1.0 cms 96.9%
90% nn ts1.0 normalized 99.9%
Matrix: lg, Window smoothing: 9, Matrix normalization: uniform
% cut gaps AUC
90% nn ts0.5 cms 99.8%
90% nn ts0.5 normalized 99.7%
90% nn ts1.0 cms 99.6%
90% nn ts1.0 normalized 99.7%
Matrix: bl, Window smoothing: 9, Matrix normalization: bgfreq
% cut gaps AUC
90% nn ts0.5 cms 100.0%
90% nn ts0.5 normalized 99.9%
90% nn ts1.0 cms 100.0%
90% nn ts1.0 normalized 99.9%
Matrix: lg, Window smoothing: 9, Matrix normalization: bgfreq
% cut gaps AUC
90% nn ts0.5 cms 99.8%
90% nn ts0.5 normalized 99.6%
90% nn ts1.0 cms 99.6%
90% nn ts1.0 normalized 99.7%
True positive rate
False positive rate

PRST vs rProt

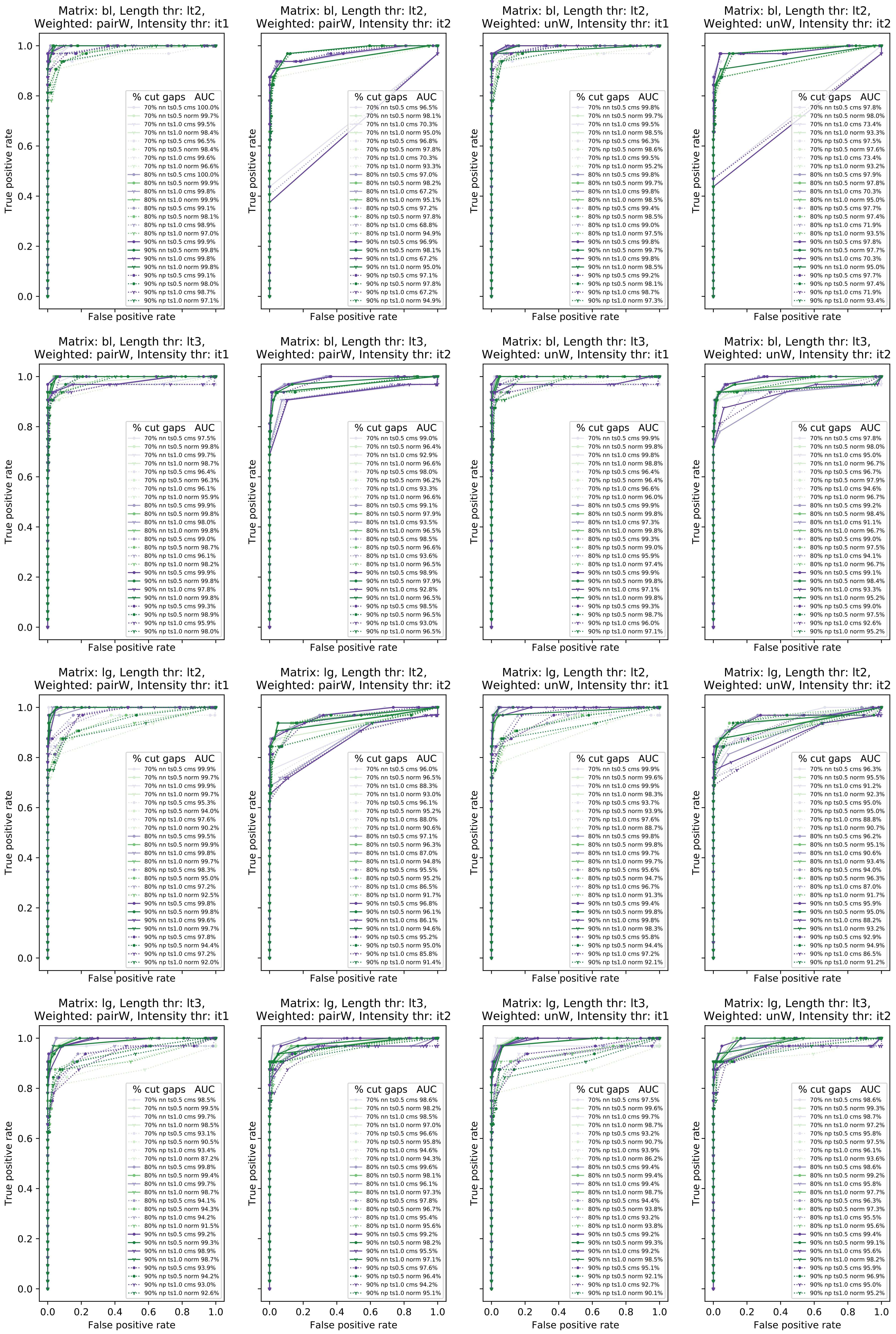

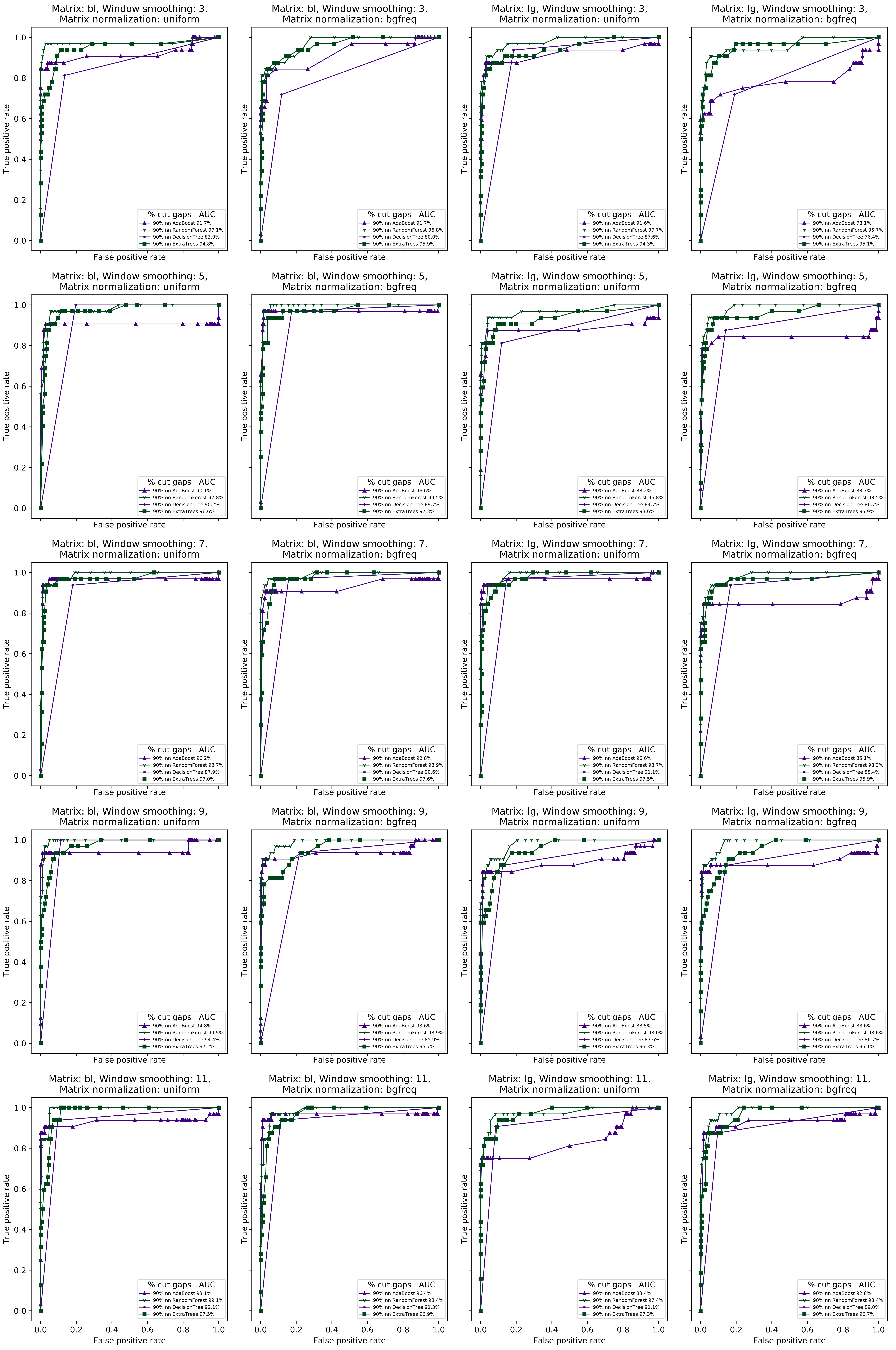
Matrix: bl, Window smoothing: 3, Matrix normalization: uniform
90% nn AdaBoost 91.7%
90% nn RandomForest 97.1%
90% nn DecisionTree 83.9%
90% nn ExtraTrees 94.8%
Matrix: bl, Window smoothing: 3, Matrix normalization: bgfreq
90% nn AdaBoost 91.7%
90% nn RandomForest 96.8%
90% nn DecisionTree 80.0%
90% nn ExtraTrees 95.9%
Matrix: lg, Window smoothing: 3, Matrix normalization: uniform
90% nn AdaBoost 91.6%
90% nn RandomForest 97.7%
90% nn DecisionTree 87.6%
90% nn ExtraTrees 94.3%
Matrix: lg, Window smoothing: 3, Matrix normalization: bgfreq
90% nn AdaBoost 78.1%
90% nn RandomForest 95.7%
90% nn DecisionTree 76.4%
90% nn ExtraTrees 95.1%
Matrix: bl, Window smoothing: 5, Matrix normalization: uniform
90% nn AdaBoost 90.1%
90% nn RandomForest 97.8%
90% nn DecisionTree 90.2%
90% nn ExtraTrees 96.6%
Matrix: bl, Window smoothing: 5, Matrix normalization: bgfreq
90% nn AdaBoost 96.6%
90% nn RandomForest 99.5%
90% nn DecisionTree 89.7%
90% nn ExtraTrees 97.3%
Matrix: lg, Window smoothing: 5, Matrix normalization: uniform
90% nn AdaBoost 88.2%
90% nn RandomForest 96.8%
90% nn DecisionTree 84.7%
90% nn ExtraTrees 93.6%
Matrix: lg, Window smoothing: 5, Matrix normalization: bgfreq
90% nn AdaBoost 83.7%
90% nn RandomForest 98.5%
90% nn DecisionTree 86.7%
90% nn ExtraTrees 95.9%
Matrix: bl, Window smoothing: 7, Matrix normalization: uniform
90% nn AdaBoost 96.2%
90% nn RandomForest 98.7%
90% nn DecisionTree 87.9%
90% nn ExtraTrees 97.0%
Matrix: bl, Window smoothing: 7, Matrix normalization: bgfreq
90% nn AdaBoost 92.8%
90% nn RandomForest 98.9%
90% nn DecisionTree 90.6%
90% nn ExtraTrees 97.6%
Matrix: lg, Window smoothing: 7, Matrix normalization: uniform
90% nn AdaBoost 96.6%
90% nn RandomForest 98.7%
90% nn DecisionTree 91.1%
90% nn ExtraTrees 97.5%
Matrix: lg, Window smoothing: 7, Matrix normalization: bgfreq
90% nn AdaBoost 85.1%
90% nn RandomForest 98.3%
90% nn DecisionTree 88.4%
90% nn ExtraTrees 95.9%
Matrix: bl, Window smoothing: 9, Matrix normalization: uniform
90% nn AdaBoost 94.8%
90% nn RandomForest 99.5%
90% nn DecisionTree 94.4%
90% nn ExtraTrees 97.2%
Matrix: bl, Window smoothing: 9, Matrix normalization: bgfreq
90% nn AdaBoost 93.6%
90% nn RandomForest 98.9%
90% nn DecisionTree 85.9%
90% nn ExtraTrees 95.7%
Matrix: lg, Window smoothing: 9, Matrix normalization: uniform
90% nn AdaBoost 88.5%
90% nn RandomForest 98.0%
90% nn DecisionTree 87.6%
90% nn ExtraTrees 95.3%
Matrix: lg, Window smoothing: 9, Matrix normalization: bgfreq
90% nn AdaBoost 88.6%
90% nn RandomForest 98.6%
90% nn DecisionTree 86.7%
90% nn ExtraTrees 95.1%
Matrix: bl, Window smoothing: 11, Matrix normalization: uniform
90% nn AdaBoost 93.1%
90% nn RandomForest 99.1%
90% nn DecisionTree 92.1%
90% nn ExtraTrees 97.5%
Matrix: bl, Window smoothing: 11, Matrix normalization: bgfreq
90% nn AdaBoost 96.4%
90% nn RandomForest 98.4%
90% nn DecisionTree 91.3%
90% nn ExtraTrees 96.9%
Matrix: lg, Window smoothing: 11, Matrix normalization: uniform
90% nn AdaBoost 83.4%
90% nn RandomForest 97.4%
90% nn DecisionTree 91.1%
90% nn ExtraTrees 97.3%
Matrix: lg, Window smoothing: 11, Matrix normalization: bgfreq
90% nn AdaBoost 92.8%
90% nn RandomForest 98.4%
90% nn DecisionTree 89.0%
90% nn ExtraTrees 96.7%
% cut gaps AUC
True positive rate
False positive rate
