# Supplementary material for: TwinCons: Conservation score for uncovering deep sequence similarity and divergence
Source: PLoS Comput Biol. 2021 Oct 29;17(10):e1009541. doi: 10.1371/journal.pcbi.1009541 (PMC8580257; doi:10.1371/journal.pcbi.1009541)
Supplement: Attachment — Submitted filename: PLOS_revision_responses_to_reviewers.docx [file pcbi.1009541.s012.docx]

**Comments to the Authors:**

**Please note here if the review is uploaded as an attachment.**

Reviewer #1: Manuscript: TwinCons: conservation score for uncovering deep sequence similarity and divergence

The manuscript presents TwinCons, a scoring function for evaluating the match between two amino acid (nucleotide) distributions observed at each position of two corresponding aligned sequence families (groups). Highly positive scores indicate the matching conservation across the families. Highly negative scores, called signatures in the manuscript, show conservation high within the families but divergent across them. While high conservation across the families indicates evolutionarily important sites, signatures may specify functionally important sites for one of the families. The TwinCons usefulness lies in the possibility to apply it to protein and nucleic acid multiple sequence alignments (MSAs). The manuscript presents results from the application of TwinCons to both types of MSAs.

We thank the reviewer for the positive summary and specific comments.

Comments:

1. TwinCons depends on a substitution matrix (SM) M in Eq. 2. Can TwinCons use multiple SMs? If so, how does it combine them?

Yes, TwinCons depends on the substitution matrix. TwinCons uses multiple structure-informed matrices, based on the LG matrix (section “Substitution matrices in TwinCons”; first 11 rows in Table S1) when running in structure-defined mode. When provided with a structure, TwinCons uses it to create an annotation of the alignment. This annotation is based on whether a specific position matches a buried or exposed residue in the structure and the type of the secondary structure there. Different structure-derived matrices (PMID: 20525635) are used, striving to match the structural annotation. We have adjusted the text in several sections to make clear when we are using structurally informed matrices (“Substitution matrices in TwinCons”, “Evaluating group similarity with TwinCons”, and “TwinCons provides convenient representation of signatures and conservation”). TwinCons uses a single substitution matrix when the only information comes from the sequence alignment.

2. The section "Generating random alignments" (line 273) lacks details of how random alignments were generated: how randomized sequence seeds were obtained and how a random tree was constructed, leading to a random alignment.

We thank the reviewer for highlighting this omission. We have included the INDELible control file, used for sequence generation, as a supplementary data (Supp. Dataset 8). INDELible generates trees and random sequence seeds within its execution and this is what we used. We have modified the methods section to make this clear (Supp. Text: section “Generating alignments from random sequences”). We moved this section to the Supplementary Material since we added a table summarizing all the datasets used in our analysis.

3. The manuscript should provide information on how the MSAs were obtained for all examples in the results section.

We thank the reviewer for highlighting this omission. We have added a section in our methods that provides more detail on the sources for the studied alignments (section “Query alignments”). Supp. Dataset 5 has been modified to provide further detail for any manual adjustments.

4. It would be useful to see both structures for the full context of the correlation between sequence-based scoring and structural observations in the section "TwinCons detects highly conserved and signature positions in composite alignments" (line 429).

We thank the reviewer for the great suggestion, we have revised Fig. 2. and added panels C and D, so that both E coli and P. furiosus structures are represented.

Also, as the manuscript benchmarks TwinCons against these methods for other use cases, a comparison with Zebra2 and HHalign conservation and signature scores (highly negative scores for HHalign), and similarly, a comparison with ConSurf and HHalign in the section "TwinCons provides convenient representation of signatures and conservation" (line 463), would be useful.

We included direct score comparisons for uL2 between Zebra and TwinCons, as well as between ConSurf and TwinCons (Supp. Dataset 10). We have further added a figure that shows the Zebra results for uL2, compared with TwinCons to the Supplementary (Fig. S4). As HHalign does not predict signatures we do not provide direct score comparison for uL2 or the caspase composite alignment.

5. Table 1 in the section "TwinCons: score that detects sequence similarity between pairs of proteins" (line 500) provides a comparison with HHalign for 8 out of 36 alignments (line 511). Would it be possible to include all alignments in the comparison?

We have added complete results of the TwinCons and HHalign analysis for all alignments to Supp. Dataset 9 and expanded our comparison section “TwinCons: score that detects sequence similarity between pairs of proteins” line 564.

6. Would it be possible to evaluate the accuracy of the tested methods, e.g., by quantifying the number of true and false positives and negatives for identified signatures?

To evaluate the number of true positives and negatives for identified signatures one would need to know the base truth for a given dataset. This is relatively straightforward when being applied for conservation since the base truth can be inferred from known ancestries of proteins and shared structures. We have applied this in our segment detection algorithm.

Benchmarking against a true signature position of alignment is more complicated as any comparison with results from other signature-identifying methods like Zebra or Diverge will be a comparison against their performance. With this in mind, we provide a more detailed comparison of TwinCons Zebra scores for identification of signature positions. The results are compiled in Supp. Dataset 10.

7. The section "Comparison of TwinCons signature positions with Zebra2" states that a "structure-informed substitution matrix" was used for one of the use cases (line 734). The optimal configuration, however, includes the BLOSUM62 matrix (line 346). Was the same SM used in the application throughout the results section? If not, what criteria were used for selecting a substitution matrix for application?

We thank the reviewer for highlighting this omission. We applied the structure-informed matrices (LG derived PMID: 20525635) when a structure is available (in the uL2 and caspase cases). However, this was not possible in the automated segment detection algorithm as this would have involved manual identification and pairing of hundreds of structures with alignments (in the case of the PROSITE dataset probably thousands) from our training datasets. So, for parametrization we compared LG and Blosum62 (Supp. Dataset 2), and we are now using the LG matrix. We have adjusted our methods to clarify our use of different matrices (section “Substitution matrices in TwinCons” and “Evaluating group similarity with TwinCons”). Furthermore, Fig. S8 shows the improvements of using structure-informed matrices over sequence-only matrix like Blosum.

Reviewer #2: TwinCons is a score for evaluating conservation patterns within and between two groups of sequences. The authors present numerous examples of the methods utility for distinguishing conserved, nonconserved, and "signature" residues. The ability to visualize conserved and unique resides on a PDB structure is particularly nice.

We thank the reviewer for the positive commentary. We would like to further thank the reviewer for useful suggestions that enabled us to justify selection of multiple options and parameters used in the current paper, which substantially improves the strength of the manuscript. We revised implemented the suggestions and obtained the optimized parameters, which we further used throughout the example studies. We summarized the default options of the parameters and described possible alternatives. We also note that the updated results do not differ substantially from those in the original submission, demonstrating a robustness of the TwinCons approach. Below we provide the detailed responses to the specific comments.

\## Major Critiques

1. Substitution matrices are typically in logorithmic units. Why is a linear multiplication with the frequency vector appropriate? More broadly, I think additional theory tying the TWC score to the statistics of substition processes is required. For instance, if the matrix M were a Markov chain transition matrix (such as PAM scores, when properly converted to linear space), then the quantity Mx represents the expected drift of the sequence over a fixed period of time. The product yMx is then the dot product of the two frequency vectors after accounting for a fixed period of drift. Of course, for substitution matrices which are not transition matrices this theory is not valid, but in that case I think a deeper discussion could motivate the choice of scoring matrix.

Indeed, this is a very important comment. We have revised the TwinCons calculation Methodology: Summary section of the main text by stating that “The TwinCons extends the formalism of a pairwise alignment to the composite alignment between two groups” and provided a rigorous mathematical justification of this statement in the Supplementary Text, which is complemented by a discussion provided in the SI section “Extending pairwise scoring to pairs of sets”. We have adjusted our methods to better motivate and clarify the choices we made for scoring matrices throughout the manuscript (section “Substitution matrices in TwinCons”, also see comment 7 of reviewer 1).

2. Uniform distributions are used for the null model in gaps and in the matrix scaling. However, uniform distributions are not very realistic for biological sequences. Would it be better to use background frequencies instead? In some cases these can be derived from the substitution matrix (e.g. the eigenvectors of a transition matrix give the stationary distribution).

We have implemented and tested the application of background frequencies for baselining (scaling) of matrices used in TwinCons. We agree with the reviewer that this is a better option, and we made this correction as the default behavior of the program, when background frequencies are available (for a selected scoring matrix).

The background frequencies for blosum matrices were retrieved from the original files (<ftp://ftp.ncbi.nih.gov/repository/blocks/unix/blosum/blosum.tar.Z> - .out files). Matrices stored in PAML format (like LG) already hold within themselves the stationary distributions.

Following the results of our theoretical justifications on TwinCons baseline correction (Eqs 11 and 12 in Supp. Methods), we have attempted to employ additional correction for compositional bias in the composite alignment. This has been formulated by Altschul and Yu for biases between queries and databases in BLAST (PMID: 14663142; 15509610). We contacted Dr. Altschul and Dr. Yu to request a piece of code which applies this compositional adjustment on BLOSUM substitution matrices, based on the frequencies of the query and database. This code is currently part of the BLAST algorithm. The provided code is written in C and needs to be compiled on the user machine, so for now we do not include it in the python package, but we have implemented its use within TwinCons. Thus, the compositional bias correction can be used in addition to the background frequency baselining given that Altschul’s code is installed on the user machine.

3. More details are needed about the matrix scaling procedure. "Uniform scaling" (line 207) would normally suggest multiplication by a scalar factor, but to produce the expected 0 value I suppose an additive component must be used. Is there any additional scaling done between matrices to assure that the range of TWC values are comperable, in addition to having a consistent zero point? This issue is particularly important when comparing RNA and protein TwinCons scores, and may account for the saturation of rRNA scores.

The reviewer is correct in that we use an additive factor and for that reason we refer to this as Baseline correction. We have reworked the text and restructured it into a separate section “Baseline correction” line 214). We have provided the theoretical justification of this procedure in the Supplementary Text: section “TwinCons offset from the mean of its expected distribution”.

In the Baseline correction section of the main text, we also emphasized that in order to be able to compare TwinCons scores from different matrices (retaining their meaning), no additional scaling, besides the baseline correction to 0 has been made. Our decision to ignore the amplitude scaling is based on the fact that the matrix scores are log probabilities of a sample; if the range of scores differs when using different matrices, those scale differences are meaningful, because they indicate the difference of sample probabilities in trees with different ages and different branching likelihoods. To correctly handle these differences, we believe one shouldn’t arbitrarily rescale the log probabilities (by shrinking or stretching the range). These values represent variance in the data sample, unexplained by different models which can be different for RNA or protein sequences. We preserve this information within TWC and have made sure the user is aware of this (line 235).

4. The segment creation rule of two negative scores seems arbitrary. Were any other rules considered? The zero point for a particular set of parameters is going to be an approximation, both due to the scaling issue in point 3 and to the generous rounding used in many substitution matrices. It seems like a rule which included the magnitude as well as the sign would be more robust and might decrease fragmentation (lin 523).

We thank the reviewer for the insightful suggestion. We agree that setting an intensity and length thresholds for the segment delineation was done in an arbitrary way. We have reworked our segment creation algorithm by implementing a cumulative TwinCons score as a criterion for detecting the segments. The inspiration for this came from the Smith-Waterman algorithm for alignments where the traceback process starting from the highest scoring position, can be thought of as a cumulative score over the alignment length in reverse. We decided to simply calculate the cumulative representation of TwinCons by summing the TwinCons of each position with the previous one (Fig. 1C). Segment boundaries are then defined by identifying local minima and maxima on the cumulative score. Additionally, we apply a smoothing operation using the Savitzky-Golay filter. This is necessary as even a single negative score would cause the segment boundaries to terminate when using local minima and maxima. By using the smoothing operation, we also gain the ability to vary the window size for the smoothing operation.

We performed optimization of the scaling window and justified that scaling window size 9 yields optimal results in detecting TP and TN results from the training data sets (section “Parameterization of TwinCons segment calculation” Supp. Dataset 2). Thus, we used this parameter in all of our calculations in the Results section “TwinCons: score that detects sequence similarity between pairs of proteins”.
Our results did not significantly change even after completely reworking the segment delineation algorithm. It does seem however that this cumulative segment delineation has helped with some cases of fragmentation (Fig. S10), for which we thank the reviewer for their suggestion again.

5. Was SVM with rbf kernel the only classifier considered? Since the data is highly non-seperable SVM does not seem an obvious choice. Distance from the boundary is also not a very good metric for the quality of a segment. A probabilistic classifier would give a better calibrated quality score.

Indeed, in the original submission, we only used the SVM classifier with ‘rbf’ kernel. Other kernels or linear classifiers explored during early stages of this work were consistently performing poorly.

In the current revision, we added the support for probabilistic classifiers. We used the python sklearn implementation of RandomForest, ExtraTrees, and AdaBoost (<https://scikit-learn.org/stable/modules/ensemble.html#forests-of-randomized-trees>. We benchmarked the performance of SVM against these probabilistic classifiers based on forests of random decision trees).

In our parametrization tests SVM consistently outperformed the forest classifiers across different datasets and TwinCons parameters (Supp. Dataset 2). We extended our SVM classifier tests to include the gamma parameter (section “Penalty and gamma selection for the SVM classifier”, Supp. Fig. S2), which defines how far the influence of a single training example reaches on the SVM coordinate. We further provide scripts that can generate a calibrated SVM classifier with statistics for the calibration, based on separating training datasets in 3 groups of data: training, validating, and testing. Given the results of the parametrization tests and implementation of the advanced options, we selected the SVM classifier to be the default in the current study. Yet, the probabilistic classifiers are also now supported, and the users may want to use them for their needs.

Finally, we note that while a distance from decision boundary is not a probabilistic measure and, in that sense, not the best metric for the quality of a segment, we use probability rates of performance against other datasets to determine the best threshold for significant segment (section “Choice of significance threshold” and Supp. Dataset. 4). Furthermore, a probability reaches its maximum confidence at 1. Therefore, two segments with big difference in their properties can have the same maximal probability of 1 yet be at a different distance from the decision boundary (Table 2). In that way a distance from the decision boundary is a valuable metric as it enables an additional comparison of “large” segments. We have included the calculated probability to the testing script outputs for the convenience of the user in having both measures.

6. The procedure for rRNA segment creation appears to be significantly different from the protein method. The Suppl Text is a bit unclear and should be revised, but I think the fact that different thresholds were used for RNA should be discussed in the main text. The suppl text refers to k-mers in the context of k-means clustering; probably "k clusters" was intended (also S2).

We thank the reviewer for highlighting our incorrect use of terminology, we have adjusted the section “Signature and conservation thresholds” to fix that. We believe the reviewer is referring to signatures and not segments as we do not attempt to identify segments within rRNA. We use the same methodology for signature identification between rRNA and protein (caspases). We have adjusted the text to indicate that only a single threshold was used for the RNA (Section “Signature and conservation thresholds” in Supplementary Text). We used several subsets of rRNA sequences only to identify the most conservative signature threshold for rRNA, then we used that one throughout the manuscript.

7. One major limitation of TwinCons is that it relies entirely on the input alignment and the division into two groups. This is discussed towards the end (735), but I think it should be emphasized more. In particular, the source of the alignment for all examples should be clearly stated. The non-permuted bL33/aL42 alignment should be pointed out to be deliberately wrong (e.g. to align non-homologous residues), as it is presumably a low-scoring artifact.

We have altered the Abstract, Methods and Discussion sections and explicitly stated that TwinCons is computed from a user-supplied alignment with pre-defined groups (section “TwinCons requires a pre-computed composite alignment.”). We provided the sources and methodological details for all alignments used in the current study (section “Query alignments”). Furthermore, we altered the text regarding non-permuted bL33/aL42 system in the Results section of the main text (section ”Sequence similarity between the circularly permuted bL33 and aL42”). We modified the section “TwinCons requires a pre-computed composite alignment” to explicitly state the dependence of TwinCons’ on the quality of the input alignment.

\## Minor Critiques

8. Several sentences could be naively interpretted that TwinCons allows one to compare two nonhomologous genes/proteins (e.g. 'inspecting a pair of polymers' (35), 'correlated changes in RNA and protein assemblies' (778), etc). Particularly in the abstract it should be clear that TwinCons applies to alignment rows and does not address coevolution.
We have removed these sentences. We have changed the language throughout the manuscript to highlight that TwinCons is applied to specific alignment positions (Abstract) and any observed corelated changes are a result of a combined structural analysis with TwinCons scores.

9. I suggest using a diverging color palette for decision boundary distance (e.g. Fig 1C) to emphasize the boundary. (<https://seaborn.pydata.org/tutorial/color_palettes.html#perceptually-uniform-divering-palettes>)
We have changed the color palette to the Green-White-Purple one. This indeed made the decision boundary stand out better (Figs. 1 and 4).

10. The gradients in Fig 1B make it difficult to read the bar plot.

We’ve fixed the gradients to make the plot clearer.

11. The 'TwinCons Score' Section (line 115) gets into some details that are not explained until the methods section. Please consider the organization.

We have removed this paragraph from the introduction as it was restated further down in the methods in a much more detailed way (section “The TwinCons Score”). We further removed all mention of vectors in this section for the same reason.

12. "random residue distribution" should be "uniform" (line 208) to prevent confusion with background frequencies.

Thank you for helping us clarify the language.

13. Coefficients & formula for the trained SVM should be included (may be supplemental).

We have added a section in the Supplementary Text (section “SVM classifier formula and features”), which lists the formula used for the ‘rbf’ kernel, the gamma parameter, and the normalization values, used on each testing segment.

14. A table comparing the different data sets used for SVM training might be helpful (number of positive & negative examples, whether negatives were filtered, etc).

We included this information as a Table in the main text (Table 1). After adding this table, we reorganized the section by moving the details for generating each dataset to the Supplementary Text (sections “Generating alignments from random sequences” and “Generating alignments from biological sequences”).

15.Why are some sidechains visible in Fig 3? If they are important residues, perhaps they should be shown consistently across all structures.

This was an omission on our side, and we have fixed the figure by removing the sidechains from the representation.

16. If the goal of Fig 2 & 3 is to compare TwinCons to ConSurf and Zebra2 respectively, I wonder if a dotplot showing the correlation between scores directly would be more pertinent. This could be quite interesting, as comparing the different color scales can be difficult by eye.

As we show in Fig. 3, Zebra2 outputs two continuous scores – one for conserved and one for signature positions. Furthermore, ConSurf only provides results for conservation, making its inclusion in the same plot also challenging This makes it impossible to make a direct comparison between the single (TwinCons), and the two scores outputted by Zebra or the conservation-only ConSurf in a single plot. Furthermore, as dotplots are generally useful when looking for self-similarity within a single sequence, and here we deal with a pair of sequences we decided that providing the direct numerical outputs of each program will be more useful. Despite these obstacles, we have included a direct numerical comparison between TwinCons, ConSurf, and Zebra2 results for uL2 in Supplementary Dataset 10. In our comparison we also included the signature score for Zebra2.

17. The application to rProtein and rRNA trees (lin 677) was not clear to me. How can TwinCons be applied to more than one gene at a time? Particularly cases with different alphabets?

. Our text was unclear and was creating the incorrect impression. We have substantially revised this section to underline that we are referring to structural colocalization of signatures and not correlation. We have moved this section to a more logical location, next to a relevant section about the universal core of life in the discussion (section “TwinCons detects shared segments within the universal core of life”). While the alphabets between RNA and protein are different, signature positions across the bacterial and archaeal branch have long been identified and used as markers studying phylogenetic domains (PMID: 18768810; 15522791). We hope TwinCons will provide the community with a convenient tool that allows users to easily calculate, visualize, and structurally map signatures of both protein and RNA.

18. I don't understand the analogy to dipole energy (line 706). Is this just the fact that both are vector quantities?

Yes, we used this analogy to underline they are vectors. We elaborated this analogy within the text (section “TwinCons compared to alternative scores” line 735)

\## Code

19. I was able to successfully install and run the tool. Code is sufficiently documented and available in the pypi repo. The URL needs to be updated to <https://github.com/LDWLab/AlignmentScore>. It might make sense to rename the project to LDWLab/TwinCons or similar to make it easier to find.

Thank you for the suggestion, we have changed the name of the repository to https://github.com/LDWLab/TwinCons.

20. It would be nice to include in the repository a complete example. For instance, there are no pdb files available for use with TwinCons.py

Thank you! We have added the alignment and structures, used for the caspase-metacaspase example, as well as a command that should generate pymol script that colors the structures with TwinCons
